# Synthesis and biological evaluation of echinomycin analogues as potential colon cancer agent

**DOI:** 10.1038/s41598-024-58196-3

**Published:** 2024-04-01

**Authors:** Keita Kojima, Hiroaki Konishi, Kyoka Momosaki, Yuya Komatani, Akira Katsuyama, Koji Nakagawa, Kayoko Kanamitsu, Fumika Yakushiji, Mikihiro Fujiya, Satoshi Ichikawa

**Affiliations:** 1 Faculty of Pharmaceutical Sciences, Hokkaido University, Kita-12, Nishi-6, Kita-ku, Sapporo, 060-0812 Japan; 2 Department of Gastroenterology and Advanced Medical Sciences, Asahikawa Medical University, 2-1-1-1, Midorigaoka, Asahikawa, Hokkaido 078-8510 Japan; 3 Center for Research and Education on Drug Discovery, Faculty of Pharmaceutical Sciences, Hokkaido University, Kita-12, Nishi-6, Kita-ku, Sapporo, 060-0812 Japan; 4 Division of Gastroenterology, Department of Internal Medicine, Asahikawa Medical University, Midorigaoka-Higashi 2-1-1-1, Asahikawa, Hokkaido 078-8510 Japan; 5 Global Station for Biosurfaces and Drug Discovery, Global Institution for Collaborative Research and Education (GI-CoRE), Hokkaido University, Kita-12, Nishi-6, Kita-ku, Sapporo, Sapporo 060-0812 Japan; 6 School of Pharmaceutical Sciences, Health Sciences University of Hokkaido, 1757 Kanazawa, Tobetsu-cho, Ishikari-gun, Hokkaido 061-0293 Japan; 7 Lead Exploration Unit, Drug Discovery Initiative, Graduate School of Pharmaceutical Sciences, The University of Tokyo, 7-3-1 Hongo, Bunkyo-ku, Tokyo 113-0033 Japan

**Keywords:** Drug discovery, Chemistry

## Abstract

Colorectal cancer is the third most commonly diagnosed cancer and the second leading cause of cancer-related death, thus a novel chemotherapeutic agent for colon cancer therapy is needed. In this study, analogues of echinomycin, a cyclic peptide natural product with potent toxicity to several human cancer cell lines, were synthesized, and their biological activities against human colon cancer cells were investigated. Analogue **3** as well as **1** inhibit HIF-1α-mediated transcription. Notably, transcriptome analysis indicated that the cell cycle and its regulation were involved in the effects on cells treated with **3**. Analogue **3** exhibited superior in vivo efficacy to echinomycin without significant toxicity in mouse xenograft model. The low dose of **3** needed to be efficacious in vivo is also noteworthy and our data suggest that **3** is an attractive and potentially novel agent for the treatment of colon cancer.

## Introduction

Cancer is a leading cause of death from disease in many countries, and 9.9 million people died of cancer in 2020 worldwide^1^. Colorectal cancer is the third most commonly diagnosed cancer and the second leading cause of cancer-related death among men and women. The distant metastasis of cancer cells is also an important issue, a colorectal cancer tends to metastasize to the liver and lungs. In particularly, the 5-year survival rate for stage IV colorectal cancer patients with distant metastases to the liver and lungs is still extremely low. Current treatments for colorectal cancer include surgical resection and chemotherapy. However, even after successful surgical resection followed by chemotherapy, the 5-year recurrence rate is still high. Therefore, a novel chemotherapeutic agent for colon cancer therapy is urgently needed.

**Figure 1:**
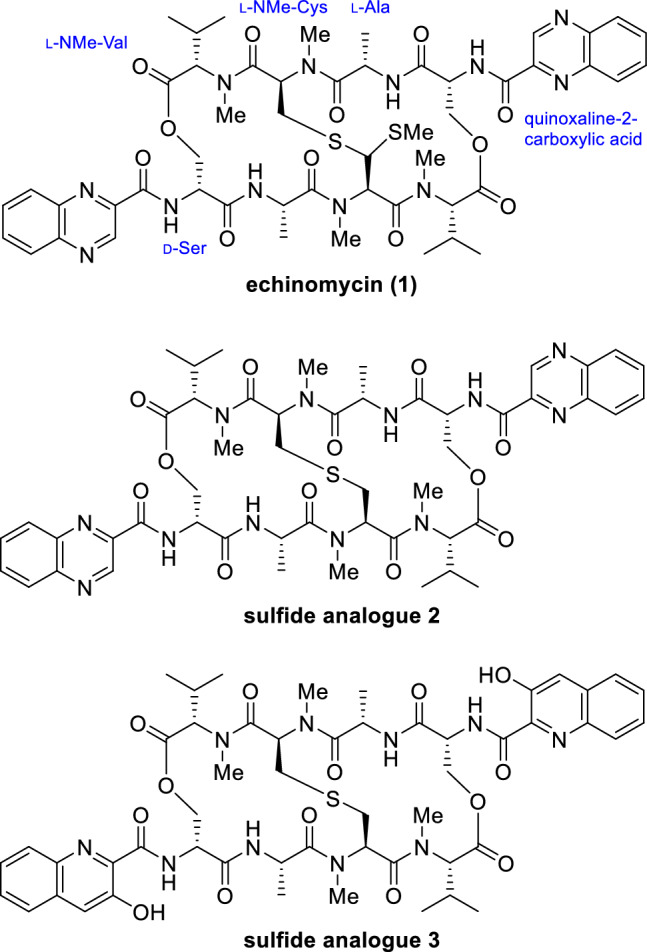
Chemical structures of echinomycin and its analogues.

Echinomycin^2^ (**1**, Fig. 1), a bicyclic octadepsipeptide possessing two quinoxaline chromophores, is in a class of natural products that can bind to double-stranded DNA due to bisintercalation by the chromophores^3–6^. Because of its potent toxicity against human cancer cells, several phase I and II clinical trials with this compound were conducted in the 1990s^3–14^. However, drug development was halted because inimal therapeutic effects were observed for terminal patients with solid tumors. Migration, angiogenesis, and invasion are important for tumor progression and metastasis and are regulated by the transcription factor, hypoxia-inducible factor-1 (HIF-1)^15,16^. In 2015, it was reported that echinomycin suppresses the HIF-1 pathway by inhibiting the binding of HIF-1α to the hypoxia-responsive element (HRE), which is the *cis*-element of HIF-1α^17^. In addition, it has been suggested that **1** suppresses the Notch signaling pathway, resulting in potent inhibitory activity against pancreatic cancer by targeting stem cells^18^. It has also been reported that **1** can selectively kill leukemia-initiating cells in patients with relapsed acute myeloid leukemia (AML) without causing normal stem cell toxicity^19^. In these studies, the dose of **1** demonstrating efficacy was quite low compared to those used in previous clinical trials. It has been suggested that **1** is still a promising anticancer drug lead compound because of its ability to alter the clinical protocol. We accomplished the first total synthesis of **1** by constructing the characteristic thioacetal moiety via a Pummerer rearrangement of **2** in the late stage of the synthesis^20^. A brief structure–activity relationship study revealed that **2** reduces but still retains the toxicity of **1** to human pancreatic cancer MIA PaCa-2 cells. We further found that the chromophore analogue **3**, which possesses 3-hydroxyquinoline-2-carbonyl groups, exhibited a similar cytotoxic potency to that of **1**. The fact that sulfide analogues **2** and **3** exhibit potencies similar that of **1** is advantageous because these analogues are more synthetically accessible than **1**. The toxicity of these analogues to human colorectal cells has not yet been tested, and the structural requirements of the chromophores remain to be investigated from our previous study. In this study, the synthesis of chromophore analogues of **2** and an in vitro and in vivo evaluation of their antiproliferative activity against SW620 cell lines, which are human colon cancer cells derived from highly invasive lymph node metastases, are described.

## Results

### Synthesis of echinomycin analogues

**Figure 2:**
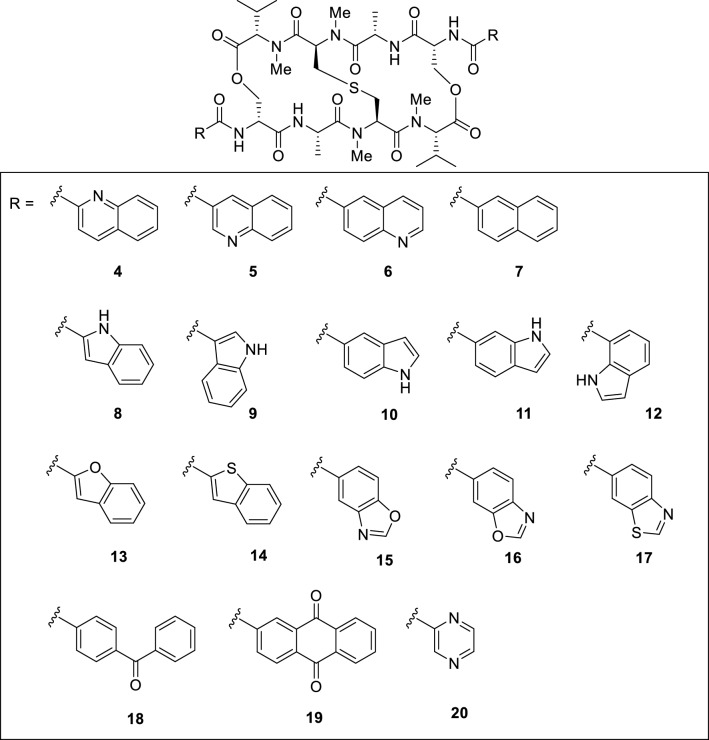
Chemical structures of echinomycin analogues.

**Figure 3:**
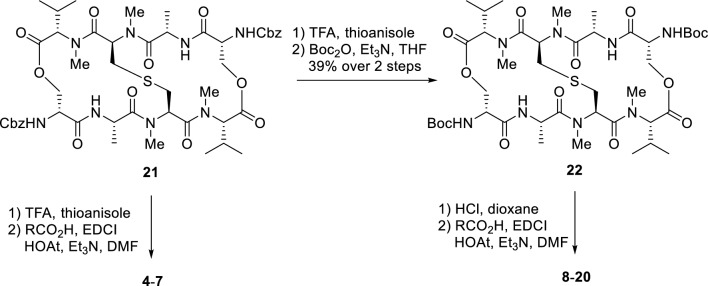
Synthesis of echinomycin analogues.

First, the impact of other pendant chromophores was investigated to expand our current knowledge of the structure–activity relationships for **2** (Fig. 2). Analogues **4**–**7** were designed to determine the impact of the number and position of each nitrogen in the quinoxalines of **2** on the cytotoxic activity. Quinoxaline is a fused aromatic group composed of two six-membered rings. Similarly, to determine the effect of chromophore shape, analogues **8**–**17**, which have five-membered aromatic rings fused to benzene as the chromophores, **18** and **19**, which have extended chromophores, and **20**, where the phenyl rings of **2** were truncated, were designed. One of the characteristics of our synthetic strategy toward **2** and **3** is the installation of chromophores at the late stage of synthesis, which allows us to access a range of chromophore analogues efficiently. Analogues **4**–**20** were synthesized as shown in Fig. 3. Analogues **4**–**7** were synthesized by deprotection of the Cbz groups on the amines of the D-Ser residues of **21** using TFA in the presence of thioanisole followed by acylation of the resulting amines with the corresponding carboxylic acids using EDCI, HOAt, and Et_3_N in DMF. As for the synthesis of **8**–**20**, the liberated diamine obtained from **21** was once protected with the Boc group to provide **22** in 39% yield over two steps. There are two advantages for the changing the protecting group from Cbz to Boc. The first is to reduce the time for the synthesis of multiple compounds, because the Boc group is more quickly removed by under an acidic conditions than the Cbz group. The second is to simplify the purification of the products. Specifically, the benzylmethylphenylsulfonium trifluoroacetate was generated as a byproduct during Cbz deprotection of **21** using TFA and thioanisole. The sulfonium salt is difficult to separate from the liberated amine, whereas the removal of the Boc group does not generate such problematic byproducts. This change largely improved the synthetic access to a range of analogues. Deprotection of the Boc groups of **22** by HCl in dioxane followed by condensation with the corresponding carboxylic acids afforded **8**–**20**.

### Toxicity of echinomycin analogues to human colon cancer cells

With a range of chromophore analogues in hand, their toxicity to SW620 cells was investigated by WST assays. Additionally, the 50% inhibitory concentration (IC_50_) values were determined and compared to those of **2**, which has 2-quinoxaline chromophores, as a positive control for **3**–**20** (Table 1). To compare the toxicities of the new analogues to pancreatic cancer cells with the toxicity of **1** side by side, their activity against SW620 cells and MIA PaCa-2 cells is also shown in Table 1. Similar to a previous investigation with MIA PaCa-2 cells^20^, **2** retained its potent toxicity to SW620 cells with an IC_50_ value of 26 nM although a tenfold reduction in the activity was observed. Analogue **3**, which has 3-hydroxyquinoline chromophores, exhibited cytotoxicity equipotent to that of **1** (2.5 nM for **1** vs. 0.85 nM for **3**). Under these conditions, the 2-quinoline analogue **4** showed slightly better cytotoxicity than **2** (IC_50_ 26 nM), indicating that the nitrogens at the 4-positions of the quinoxaline chromophores in **2** are not necessary for cytotoxicity. In contrast to **4**, analogues **5** and **6**, where the nitrogens at the 1-positions of the chromophores in **4** are transposed, displayed largely reduced activity, with IC_50_ values of 1210 and 1193 nM, respectively. Naphthalene analogue **7**, which lacks all the nitrogens in **2**, showed completely diminished activity. Analogues **8**–**17** possess 5-membered heterocycles fused to the benzene ring. This structural alteration has a great impact on cytotoxicity, and all of these analogues completely lost their activity (IC_50_ > 10,000 nM). Extended chromophore analogues **18** and **19** as well as truncated analogue **20** also lost their cytotoxicity.

**Table 1:** Cytotoxicity of **1** and its analogues.

Compounds	IC_50_ (nM)^a^	
	SW620^b^	MIA PaCa-2^c^
**1**	2.5 ± 0.8	0.75 ± 0.02
**2**	25.6 ± 6.0	8.1 ± 1.0
**3**	0.85 ± 0.13	1.4 ± 0.1
**4**	20.5 ± 3.1	5.8 ± 0.9
**5**	1210 ± 35	399 ± 76
**6**	1193 ± 187	327 ± 66
**7**	> 10,000	> 10,000
**8**	> 10,000	> 10,000
**9**	> 10,000	> 10,000
**10**	> 10,000	> 10,000
**11**	> 10,000	> 10,000
**12**	> 10,000	> 10,000
**13**	> 10,000	> 10,000
**14**	> 10,000	> 10,000
**15**	> 10,000	> 10,000
**16**	> 10,000	> 10,000
**17**	> 10,000	> 10,000
**18**	> 10,000	> 10,000
**19**	> 10,000	> 10,000
**20**	45% inhibition at 10 µM	55% inhibition at 10 mM

^a^50% inhibitory concentration. Each data is shown as mean ± SE (n = 3).

^b^Human colon cancer cell.

^c^Human pancreatic carcinoma cell.

### Induction of apoptosis by **3**

**Figure 4:**
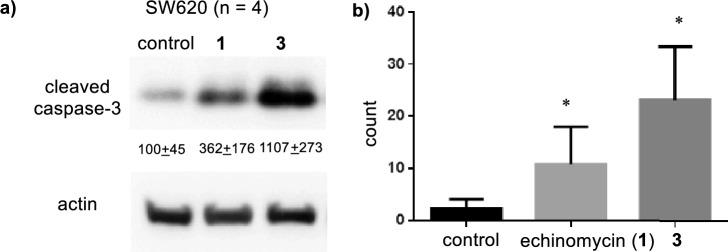
Apoptotic effect of echinomycin and **3** on SW620 cells evaluated by (**a**) Western blotting to measure cleaved caspase-3 and (**b**) TUNEL assay. *Indicates *P* < 0.005 by Student's *t* test. The error bars show the s.d. (n = 4).

Compound **3** exhibits very potent toxicity to SW620, as expected. Therefore, the expression of cleaved caspase-3 in SW620 cells was subsequently evaluated to clarify the effects of **3** on cancer cell apoptosis. Western blotting analysis of SW620 cells treated with **3** revealed that the expression of cleaved caspase-3 was significantly greater than that in untreated cells (Fig. 4). TUNEL staining was then performed. The number of apoptotic SW620 cells increased after treatment with **3** compared to the number of apoptotic control cells. These data indicated that **3** exhibited a tumor-suppressive effect by inducing colon cancer cell apoptosis. In both cases, **3** exhibited better activity than **1**.

### Inhibition of the HIF-1 pathway

**Figure 5:**
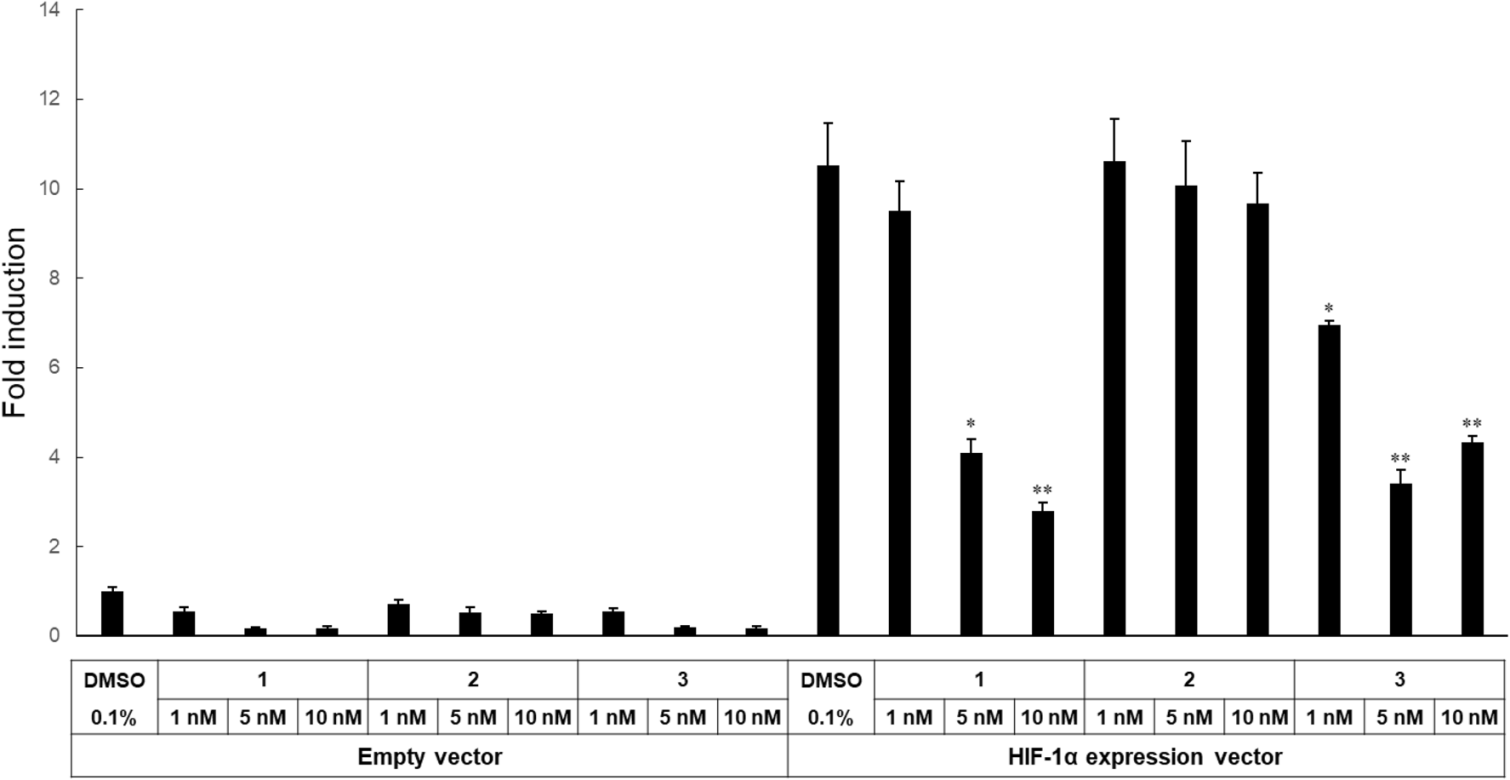
Effect of echinomycin (**1**), **2** and **3** on HIF-1α-dependent transcription in SW620 cells. SW620 cells were transiently transfected with empty vector pCI-neo-3 × FLAG or HIF-1α expression vector pCI-neo-3 × FLAG-HIF-1α, together with pGL3-5 × HRE-Luc and pGL4.75 [hRluc/CMV]. One day after transfection, echinomycin (**1**), **2**, or **3** was added to the cell culture media and incubated for an additional 16 h. The cells were lysed, and a dual luciferase assay was performed. Firefly luciferase activity was normalized to *Renilla* luciferase activity, and the data are presented as fold induction relative to the values obtained in cells DMSO-treated cells transfected with empty vector. The error bars represent standard deviations (n = 3). *P < 0.05 and **P < 0.01 compared to DMSO-treated cells transfected with HIF-1α (Student’s test).

Echinomycin is known to inhibit the HIF-1 pathway by inhibiting the binding of HIF-1α to HRE^15,21,22^. The ability of **3** to inhibit HIF-1α-mediated transcription was investigated by using an HRE reporter gene assay in SW620 cells transiently overexpressing HIF-1α. As shown in Figs. 3 and 5 markedly decreased HIF-1α-induced HRE reporter activity to a similar extent as echinomycin, indicating that **3** also has an inhibitory effect on HIF-1α-mediated transcription (Fig. 4).

### Transcriptome analysis

Although modulation of the HIF-1 pathway has been reported to be one of the modes of action of **1**, it has also been suggested that **1** inhibits other signaling pathways including the Notch pathway^19^. Therefore, transcriptome analysis was then performed to gain insight into the mode of action of **3**. Namely, to determine the changes in mRNA expression in SW620 cells treated with **3**, a high-throughput sequencing analysis was conducted in a manner similar to our previous study^23^.

Seven thousand and three hundred seventy mRNAs exhibited more than twofold changes in expression levels that were significant (*p* < 0.05) in comparison to their expression levels in control cells (Supplementary Table 1). Pathway analysis was performed using the software program MetaCore, and the results are shown in Table 2. These results indicated that the cell cycle and its regulation were involved in the changes in the expression of these mRNAs in the cells treated with **3**. Notably the HIF-1 pathway was not indicated by this analysis, suggesting that the HIF-1 pathway may not be the primary target of **3**.

**Table 2:** The pathway analysis performed using the MetaCore software program.

No.	Maps
1	Cell cycle_Initiation of mitosis
2	DNA damage_ATM/ATR regulation of G2/M checkpoint
3	Cell cycle_Chromosome condensation in prometaphase
4	Cell cycle_The metaphase checkpoint
5	Cell cycle_Role of 14–3–3 proteins in cell cycle regulation
6	DNA damage_ATM/ATR regulation of G1/S checkpoint
7	Cell cycle_Role of APC in cell cycle regulation
8	Cell cycle_Cell cycle (generic schema)
9	Cell cycle_Start of DNA replication in early S phase
10	Development_Positive regulation of STK3/4 (Hippo) pathway and negative regulation of YAP/TAZ function
11	Development_Negative regulation of STK3/4 (Hippo) pathway and positive regulation of YAP/TAZ function
12	Cell cycle_Role of SCF complex in cell cycle regulation
13	Cell cycle_Spindle assembly and chromosome separation
14	Cell cycle_Nucleocytoplasmic transport of CDK/Cyclins
15	Cell cycle_Influence of Ras and Rho proteins on G1/S Transition
16	Apoptosis and survival_Role of PKR in stress-induced apoptosis
17	Cell cycle_Role of Nek in cell cycle regulation
18	Signal transduction_mTORC1 downstream signaling
19	Cell cycle_Regulation of G1/S transition (part 1)
20	Cell cycle_Sister chromatid cohesion

The cell cycle and its regulation pathway were markedly altered in the cells treated with **3**.

**Figure 6:**
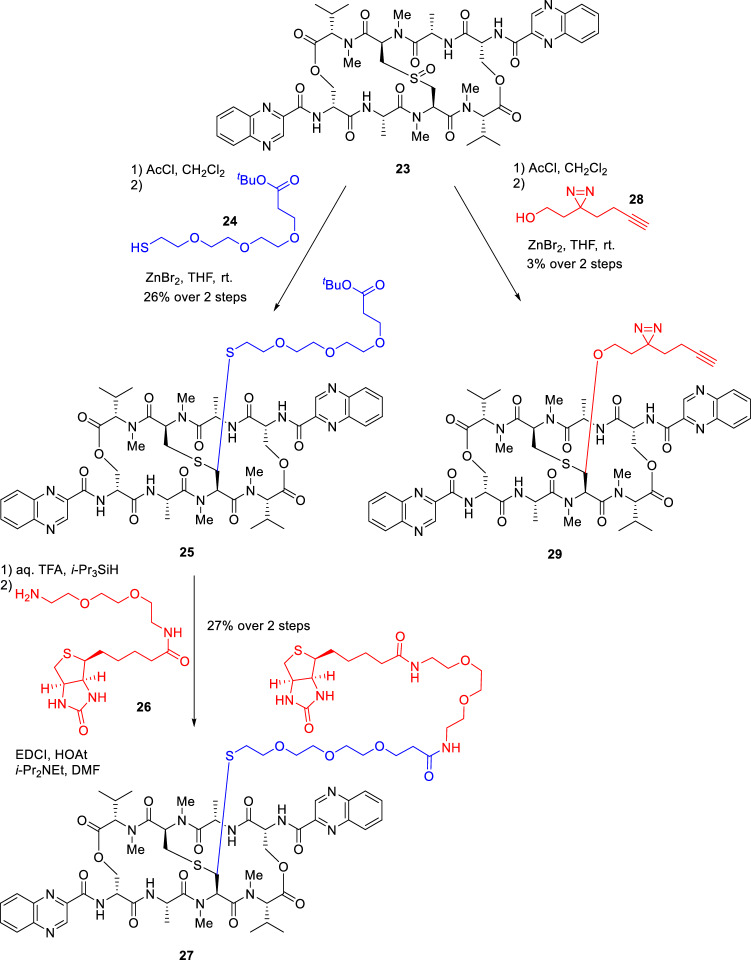
Synthesis of echinomycin probes.

To identify potential target molecules of echinomycin, two types of probe compounds were designed (Fig. 6). The first one was biotinylated probe **27** with an ethylene glycol linker. Target identification using this type of probe is one of the most frequently used techniques. The other one was photochemical probe **29** with a diazirine photo cross-linker containing an alkyne handle, which was developed by Yao et al.^24^ The diazirine moiety forms a covalent bond with the target molecule by the generation of a carbene upon UV irradiation, while the alkyne is used for subsequent immobilization on the beads via a click reaction. The most important feature of this linker is that it can minimize structural changes due to the introduction of the linker, and it has been reported to be used even in living cells. These compounds were synthesized from sulfoxide **23** in a manner similar to the total synthesis of **1**, via a scheme that was developed by our group^20^. Namely, Pummerer rearrangement of **23** by AcCl in CH_2_Cl_2_ gave the corresponding chloride, which was sequentially treated with **24** as a nucleophile and ZnCl_2_ as a promoter in THF at room temperature for 15 h to provide **25** in 26% yield over two steps from **23**.

After removal of the *t*-Bu group of **25** by *aq*. TFA, the resulting carboxylic acid was condensed with biotinylated amine **26** to afford the desired biotinylated probe **27**. Chemical probe **29** was synthesized using the minimalist linker **28**. Identification of the target molecule(s) of echinomycin using **27** and **29** is currently underway.

### Metabolic stability of 3

Before investigating the in vivo efficacy of **3**, its metabolic stability was evaluated by treatment with human or mouse liver microsomes at 37 °C for 30 min, and the remaining analogues were analyzed by LC–MS/MS (Table 3). The clearance vale of **3** were 83.7 and 386 mL/min/kg for human and mouse liver microsomes, respectively, in the presence of NADPH. This means that approximately 67% of **3** was unaffected by human liver microsomes. Thus, it was revealed that these analogues were metabolically stable, especially in human liver microsomes. Furthermore, the metabolic clearance values in the absence of NADPH were 45.1 and 118 mL/min/kg in human and mouse liver microsomes, respectively, suggesting that in liver microsomes in both species, approximately half of the metabolic reactions are oxidative reactions and the other half are hydrolytic reactions involving the peptide and/or ester bonds in **3**.

**Table 3:** Metabolic stability of echinomycin (**1**) and **3** (mL/min/kg).

	**1**		**3**		Midazolam	
	NADPH(+)	NADPH(−)	NADPH(+)	NADPH(−)	NADPH(+)	NADPH(−)
Human	< 22.0	< 22.0	83.7	45.1	435	< 22.0
Mouse	< 69.8	< 69.8	386	118	4006	< 69.8

### Investigation of the maximum tolerated dose of **3**

Toxicity Body weight change is an indicator of systemic toxicity. Accordingly, the body weights of mice intraperitoneally treated with different doses (0.04 and 0.4 µg/mouse) of either **1** or **3** were measured (Fig. S1). After treatment with dosages of 0.04 and 0.4 µg/mouse, the body weights did not differ greatly from those of the control, indicating that **1** and **3** did not exhibit severe systemic toxicity at these dosages administered over 14 days. A blood hematological examination (including nineteen parameters) was also conducted on these mice on Day 14 (Fig. S2). There were no significant changes in almost any of the parameters in the mice treated with either **1** or **3** at doses of 0.04 or 0.4 µg/mouse. At a dose of 0.4 µg/mouse, a decrease in total bilirubin (T-BIL) and an increase in aspartate aminotransferase (AST), alanine aminotransferase (ALT), and lactate dehydrogenase (LDH) were observed in both groups treated with **1** and **3** (indicated by arrows). These results indicate that **1** and **3** do not show significant toxicity at least up to a dose of 0.04 µg/mouse, which is expected to be the maximum tolerated dose of **3** for the subsequent in vivo evaluations.

### Tumor growth suppression by **3** in vivo

**Figure 7:**
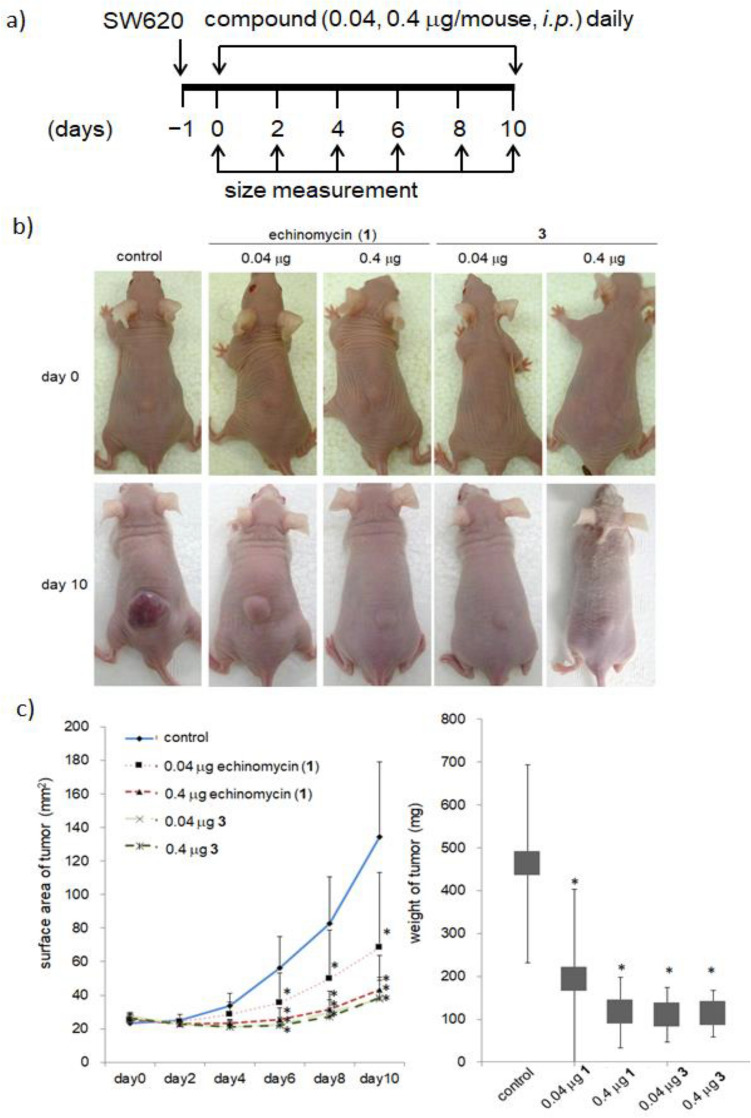
In vivo anticancer activity of echinomycin and **3** on the SW620 xenograft mice model. Male BALB/C nude mice were administered by intraperitoneal injection with the vehicle, echinomycin and **3**, at dosages of 0.04 and 0.4 µg/mice (n = 10 each group), every day for 10 days (**a**, **b**). Average tumor volume (**c**) was measured. Data are expressed as the mean ± standard error (n = 8). *Indicates *P* < 0.05 by Student's *t* test. The error bars show the s.d. (n = 10).

Since the metabolic stability and identification of the maximum tolerated dose of **3** were confirmed, the effect of **3** on colon cancer progression in vivo was then investigated (Fig. 7). A xenograft model was generated by injecting a suspension of 2 × 10^6^ SW620 cells into the backs of nude mice. The doses of the compounds were 0.04 and 0.4 µg/mouse, which correspond to approximately 1.6 and 16 μg/kg, respectively, according to the results obtained in Fig. S1. A solution of **1**, **3**, or PBS was injected intraperitoneally into each mouse, and tumor sizes were measured each day. Tumor growth in the groups treated with **1** and **3** was significantly suppressed compared to that in the PBS-injected group. Of note is the efficacy of tumor growth inhibition with **3**. The efficacy of **3** at a dose of 0.04 µg/mouse is equivalent to that of **1** at a dose of 0.4 µg/mouse, and it is revealed that **3** possesses superior tumor growth inhibition properties to **1**.

## Discussion

**Figure 8:**
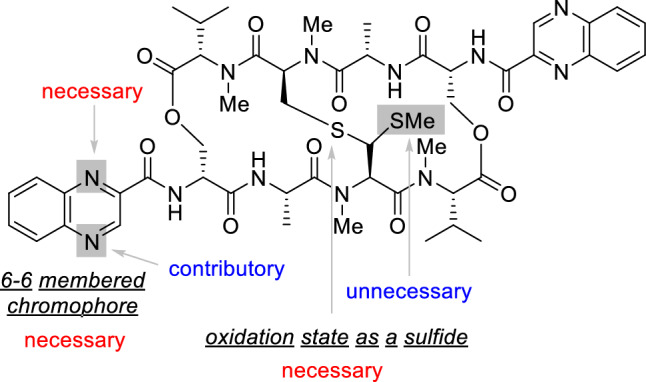
Summary of SAR of echinomycin.

Echinomycin analogues were synthesized and biologically evaluated in this study. Evaluating the impact of other pendant chromophores was considered the first goal of this study, with which we aimed to significantly expand our current knowledge on the structure–activity relationship of **2**. Analogue **3** was designed inspired by the fact that congener natural products of **1**, such as SW-163C^25^, thiocoraline^26^, sandramycin^27^, and quinaldopeptin^28^, share 3-hydroxyquinoline-2-carbonyl groups as chromophores and display strong toxicity to a range of human cancer cell lines. An investigation of the cytotoxicity of these analogues to SW620 cells revealed the following structure–activity relationship for the chromophore. (1) The nitrogens at the 4-positions of the quinoxaline chromophores in **2** are not necessary. (2) The nitrogens at the 1-position contribute significantly to the cytotoxicity. (3) Naphthalene analogue **7**, which lacks all the nitrogens of **2**, displayed completely diminished activity. (4) The shape of the 6-membered heterocycles fused to the benzene ring is important for cytotoxicity. The SARs of echinomycin elucidated in this study in conjunction with those previously reported^20^ are summarized in Fig. 8.

Analogue **3** as well as **1** inhibited HIF-1α-mediated transcription (Fig. 4). It has also been suggested that **1** inhibits other signaling pathways including the Notch pathway^19^. Our transcriptome analysis indicated that treatment with **3** altered the expression of 7370 mRNAs in SW620 cells. Pathway analysis revealed that the cell cycle and its regulation were involved in these changes in mRNA expression in the cells treated with **3** (Table 2). Interestingly, the HIF-1 and NOTCH signaling pathways were not identified as the main pathways in this analysis. Further studies will be necessary to elucidate the detailed modes of action of **3** as well as **1** by using chemical probes **27** and **29**.

Analogue **3** showed lower clearance values with mouse and human liver microsomes. Although the observed difference in metabolic stability could arise from differences in the chemical stability of a thioacetal and thioether moieties or from conformational changes throughout the entire cyclic peptide caused by changes in the thioacetal linkage, more detailed studies are needed to determine which contribution is greater.

Echinomycin analogue **3** exhibited superior in vivo efficacy to echinomycin without significant toxicity in mouse xenograft model. The low dose of **3** needed in vivo efficacy is also noteworthy. The chemical structures of echinomycin and **3** are not very different and their in vitro cytotoxic activities are similar. However, the in vivo activity of **3** was better than that of echinomycin, even though the clearance of **3** was worse. One possible reason is the difference in the mechanism of **1** and **3**, which was suggested by our transcriptome analysis. Although the details are not apparent, the HIF-1 pathway likely influences the difference between the in vivo efficacy of **1** and **3**. Another reason for this difference is the difference in protein binding rates. Namely, in vivo bioactivity tends to be worse for protein binding rates that are higher at the same plasma concentration. On the other hand, the higher the protein binding rate is, the slower the drug is lost from the body and the longer it stays in the body, so its contribution to the effect of the drug is greater. It is thought that the difference in in vivo activity may be due to a difference in the balance between the two. A dose of 0.04 µg/mouse corresponds to 1.6 μg/kg for mice, and a calculated human equivalent dose of 0.13 μg/kg (4.8 μg/m^2^)^29^. No obvious toxicity was observed at this dose in the mice judging from body weight loss and the blood hematological examination, while there was significant efficacy in the xenograft model. As previously noted, human clinical trials of **1** have suggested that echinomycin at doses ranging from 1200 to 2128 μg/m^2^ given intravenously is toxic. However, there was no observed toxicity at the lower doses of 60 and 120 μg/m^2^^30^. Sulfide **3** retains the cytotoxic activity of **1** both in vitro and in vivo and is more accessible and scalable than **1**. Our data suggest that echinomycin is an attractive and potentially novel agent for the treatment of colon cancer in addition to pancreatic cancer and AML.

## Experimental section

### General experimental methods

Compounds **4**–**29** were prepared using methods previously reported by our group^20^. Namely, all reactions except those carried out in the aqueous phase were performed under an argon atmosphere unless otherwise noted. Materials were purchased from commercial suppliers and used without further purification unless otherwise noted. Solvents were distilled according to the standard protocol. Isolated yields were calculated by weighing products. The weight of the starting materials and the products were not calibrated. Analytical thin layer chromatography (TLC) was performed on Merck silica gel 60F_254_ plates. Normal-phase column chromatography was performed on Merck silica gel 5715 or Wakogel 60N. Flash column chromatography was performed on Kanto Chemical Silica Gel 60N (spherical, neutral, 40–50 µm). Hi-flash column chromatography was performed on YAMAZEN Hi-Flash™ column silica gel (40 µm) or Fuji Silysia Chromatorex MB/PSQ (50–200 µm). ^1^H NMR spectra were measured in CDCl_3_, DMSO-*d*_6_ or CD_3_OD solution and reported in parts per million (δ) relative to tetramethylsilane (0.00 ppm) as an internal standard or referenced to residual solvent peak of DMSO-*d*_6_ (2.49 ppm) or CD_3_OD (3.31 ppm) using JEOL ECS400 (400 MHz) and ECX400P (400 MHz) spectrophotometers, unless otherwise noted. ^13^C NMR spectra were measured in CDCl_3_, DMSO-*d*_6_ or CD_3_OD solution and referenced to residual solvent peaks of CDCl_3_ (77.0 ppm), DMSO-*d*_6_ (39.52 ppm) or CD_3_OD (49.00 ppm) using JEOL ECS400 (100 MHz) and ECX400P (100 MHz) spectrophotometers. The coupling constant (*J*) was reported in hertz (Hz). Abbreviations of multiplicity were as follows; s: singlet, d; doublet, t: triplet, q: quartet, m: multiplet, br: broad. Data were presented as follows; chemical shift (multiplicity, integration, coupling constant). The assignment was based on ^1^H–^1^H COSY, HMBC and HMQC NMR spectra. Mass spectra were obtained on Waters MICRO MASS LCT–premier or Advion expression CMS and the mass analyzer type used for the HRMS measurements was TOF. Optical rotation was measured on a Rudolph Research Analytical Autopol IV automatic polarimeter. Liquid chromatography-mass spectrometry (LC–MS) was performed on the systems as follows: Shimadzu Prominence-i LC-2030C Plus as an HPLC system; FCV-20AH2 as a flow-line selection valve; LCMS-8040 as a Liquid chromatograph mass spectrometer, and LabSolutions as a system controller.

### General procedure for the synthesis of 4–7

Compounds were prepared using methods previously reported by our group^20^. Namely, compound **22** (25.3 mg, 25.0 µmol) in TFA (2.5 mL) was treated with thioanisole (117.2 µL, 1.00 mmol) at room temperature for 24 h. The reaction mixture was concentrated *in vacuo*, and then washed with Et_2_O (5 mL × 3) to afford a pale yellow solid. The residue, ^*i*^Pr_2_NEt (25.5 µL, 125 µmol), carboxylic acid (100 µmol) and HOAt (17.0 mg, 125 µmol) in DMF (1.0 mL) was treated with EDCI (24.0 mg, 100 µmol) at 0 °C, and the whole mixture was stirred at room temperature for 5 h. The reaction mixture was diluted with EtOAc (5 mL), and washed with H_2_O (5 mL), 1 M *aq*. HCl (5 mL), *sat*. *aq*. NaHCO_3_ (5 mL) and brine (5 mL). The organic layer was dried over Na_2_SO_4_, filtered, and concentrated *in vacuo*. The residue was purified to afford **4**–**7**.

### {N-(Quinoline-2-carbonyl)-D-Ser-L-Ala-N-Boc-N-Me-L-Cys-N-Me-L-Val}_2_ sulfide (4)

Compound **4** (5.3 mg, 5.0 µmol, 20%) was synthesized using methods previously reported by our group^20^, from quinaldic acid (100 µmol), and purified with flash silica gel column chromatography ($\Phi$ 0.7 × 5.2 cm, CHCl_3_/MeOH: 100/0 → 99.5/0.5 → 99/1 → 98.5/1.5) as a white amorphous solid. ^1^H NMR (CDCl_3_, 400 MHz) δ 8.95 (d, 2H, Ser-N*H*, *J*_Ser-N*H*, Ser-α-C*H*_ = 7.2 Hz), 8.30 (d, 2H, Ar–*H*, *J* = 8.2 Hz), 8.24 (d, 2H, Ar–*H*, *J* = 8.2 Hz), 7.89–7.84 (m, 4H, Ar–*H*), 7.70 (ddd, 2H, Ar–*H*, *J* = 1.3, 6.9, 8.2 Hz), 7.58 (ddd, 2H, Ar–*H*, *J* = 1.1, 6.9, 8.0 Hz), 6.84 (d, 2H, Ala-N*H*, *J*_Ala-N*H*, Ala-α-C*H*_ = 7.4 Hz), 6.26 (dd, 2H, Cys-α-C*H*, *J*_Cys-α-C*H*, Cys-β-C*H*_ = *J*_Cys-α-C*H*, Cys-β-C*H*_ = 6.6 Hz), 5.13 (d, 2H, Val-α-C*H*, *J*_Val-α-C*H*, Val-β-C*H*_ = 10.0 Hz), 4.89–4.78 (m, 6H, Ala-α-C*H*, Ser-α-C*H*, Ser-β-C*H*), 4.67 (dd, 2H, Ser-β-C*H*, *J*_Ser-β-C*H*, Ser-α-C*H*_ = 1.4,* J*_Ser-β-C*H*, Ser-β-C*H*_ = 11.4 Hz), 3.38 (dd, 2H, Cys-β-C*H*, *J*_Cys-β-C*H*, Cys-α-C*H*_ = 6.6, *J*_Cys-β-C*H*, Cys-β-C*H*_ = 14.8 Hz), 3.24 (s, 6H, NC*H*), 2.95 (s, 6H, NC*H*), 2.52–2.45 (m, 2H, Cys-β-C*H*), 2.36 (qqd, 2H, Val-β-C*H*,* J*_Val-β-C*H,* Val-γ-C*H*_ = *J*_Val-β-C*H,* Val-γ-C*H*_ = 6.9,* J*_Val-β-C*H,* Val-α-C*H*_ = 10.0 Hz), 1.34 (d, 6H, Ala-β-C*H*, *J*_Ala-β-C*H*, Ala-α-C*H*_ = 6.9 Hz), 1.10 (d, 6H, Val-γ-C*H*, *J*_Val-γ-C*H*, Val-β-C*H*_ = 6.9 Hz), 0.92 (d, 6H, Val-γ-C*H*, *J*_Val-γ-C*H*, Val-β-C*H*_ = 6.9 Hz); ^13^C NMR (CDCl_3_, 100 MHz) δ 173.2, 171.3, 170.3, 168.0, 165.6, 148.5, 146.6, 138.0, 130.5, 129.8, 129.5, 128.5, 128.1, 118.9, 64.2, 62.3, 55.0, 53.5, 46.3, 36.1, 31.7, 30.3, 28.1, 20.5, 19.4, 17.6; ESIMS-LR *m*/*z* 1075.2 [(M + Na)^+^]; ESIMS-HR calcd for C_52_H_65_N_10_O_12_S 1053.4499, found 1053.4515; [α]^22^_D_ −172.8 (*c* 0.14, CHCl_3_).

### {N-(Quinoline-3-carbonyl)-D-Ser-L-Ala-N-Boc-N-Me-L-Cys-N-Me-L-Val}_2_ sulfide (5)

Compound **5** (4.6 mg, 4.4 µmol, 17%) was synthesized using methods previously reported by our group^20^, from 3-quinolinecarboxylic acid (17.3 mg, 100 µmol), and purified with flash silica gel column chromatography ($\Phi$ 0.7 × 5.2 cm, CHCl_3_/MeOH: 100/0 → 99/1 → 98.5/1.5 → 98/2 → 97.5/2.5 → 97/3) as a white amorphous solid. ^1^H NMR (DMSO-*d*_6_, 400 MHz) δ 9.26 (d, 2H, Ar–*H*, *J* = 2.2 Hz), 8.83 (d, 2H, Ar–*H*, *J* = 2.2 Hz), 8.80 (d, 2H, Ser-N*H*, *J*_Ser-N*H*, Ser-α-C*H*_ = 7.9 Hz), 8.14 (d, 2H, Ar–*H*, *J* = 7.6 Hz), 8.10 (d, 2H, Ar–*H*, *J* = 8.8 Hz), 7.91–7.86 (m, 4H, Ala-N*H*, Ar–*H*), 7.72 (td, 2H, Ar–*H*, *J* = 1.4, 7.7 Hz), 6.29 (dd, 2H, Cys-α-C*H*, *J*_Cys-α-C*H*, Cys-β-C*H*_ = *J*_Cys-α-C*H*, Cys-β-C*H*_ = 6.6 Hz), 4.84 (d, 2H, Val-α-C*H*, *J*_Val-α-C*H*, Val-β-C*H*_ = 10.8 Hz), 4.71 (ddd, 2H, Ser-α-C*H*,* J*_Ser-α-C*H*, Ser-β-C*H*_ = 2.9, *J*_Ser-α-C*H*, Ser-β-C*H*_ = 7.7, *J*_Ser-α-C*H*, Ser-N*H*_ = 7.9 Hz), 4.58 (dd, 2H, Ala-α-C*H*, *J*_Ala-α-C*H*, Ala-β-C*H*_ = *J*_Ala-α-C*H*, Ala-N*H*_ = 6.6 Hz), 4.50–4.45 (m, 2H, Ser-β-C*H*), 4.37 (dd, 2H, Ser-β-C*H*, *J*_Ser-β-C*H*, Ser-α-C*H*_ = 2.9,* J*_Ser-β-C*H*, Ser-β-C*H*_ = 13.0 Hz), 3.17–3.07 (m, 2H, Cys-β-C*H*), 3.03 (s, 6H, NC*H*), 2.81 (s, 6H, NC*H*), 2.53–2.49 (m, 2H, Cys-β-C*H*), 2.23 (qqd, 2H, Val-β-C*H*,* J*_Val-β-C*H,* Val-γ-C*H*_ = 6.4,* J*_Val-β-C*H,* Val-γ-C*H*_ = 6.8,* J*_Val-β-C*H,* Val-α-C*H*_ = 10.8 Hz), 1.27 (d, 6H, Ala-β-C*H*, *J*_Ala-β-C*H*, Ala-α-C*H*_ = 6.6 Hz), 1.00 (d, 6H, Val-γ-C*H*, *J*_Val-γ-C*H*, Val-β-C*H*_ = 6.4 Hz), 0.76 (d, 6H, Val-γ-C*H*, *J*_Val-γ-C*H*, Val-β-C*H*_ = 6.8 Hz); ^13^C NMR (DMSO-*d*_6_, 100 MHz) δ172.3, 170.3, 169.8, 167.7, 165.3, 149.0, 148.5, 136.1, 131.4, 129.2, 128.8, 127.5, 126.7, 126.4, 64.7, 61.5, 54.1, 53.1, 46.1, 35.2, 30.5, 29.7, 26.5, 20.2, 18.8, 16.6; ESIMS-LR *m*/*z* 1075.4 [(M + Na)^+^]; ESIMS-HR calcd for C_52_H_65_N_10_O_12_S 1053.4499, found 1053.4506; [α]^23^_D_ −175.3 (*c* 0.20, DMSO).

### {N-(Quinoline-6-carbonyl)-D-Ser-L-Ala-N-Boc-N-Me-L-Cys-N-Me-L-Val}_2_ sulfide (6)

Compound **6** (5.0 mg, 4.8 µmol, 19%) was synthesized using methods previously reported by our group^20^, from 6-quinolinecarboxylic acid (17.3 mg, 100 µmol), and purified with flash silica gel column chromatography ($\Phi$ 0.7 × 6.3 cm, CHCl_3_/MeOH: 100/0 → 99/1 → 98.5/1.5 → 98/1 → 97.5/2.5 → 97/3 → 95/5) as a white amorphous solid. ^1^H NMR (DMSO-*d*_6_, 400 MHz) δ 9.00 (dd, 2H, Ar–*H*, *J* = 1.8, 4.2 Hz), 8.70 (d, 2H, Ser-N*H*, *J*_Ser-N*H*, Ser-α-C*H*_ = 7.8 Hz), 8.53–8.51 (m, 4H, Ar–*H*), 8.17–8.11 (m, 4H, Ar–*H*), 7.85 (d, 2H, Ala-N*H*, *J*_Ala-N*H*, Ala-α-C*H*_ = 5.2 Hz), 7.63 (dd, 2H, Ar–*H*, *J* = 4.2, 8.6 Hz), 6.28 (dd, 2H, Cys-α-C*H*, *J*_Cys-α-C*H*, Cys-β-C*H*_ = *J*_Cys-α-C*H*, Cys-β-C*H*_ = 6.8 Hz), 4.86 (d, 2H, Val-α-C*H*, *J*_Val-α-C*H*, Val-β-C*H*_ = 10.4 Hz), 4.71 (ddd, 2H, Ser-α-C*H*,* J*_Ser-α-C*H*, Ser-β-C*H*_ = 3.0, *J*_Ser-α-C*H*, Ser-N*H*_ = 7.8, *J*_Ser-α-C*H*, Ser-β-C*H*_ = 8.0 Hz), 4.59–4.47 (m, 4H, Ala-α-C*H*, Ser-β-C*H*), 4.33 (dd, 2H, Ser-β-C*H*, *J*_Ser-β-C*H*, Ser-α-C*H*_ = 3.0,* J*_Ser-β-C*H*, Ser-β-C*H*_ = 11.4 Hz), 3.09 (dd, 2H, Cys-β-C*H*, *J*_Cys-β-C*H*, Cys-α-C*H*_ = 6.8, *J*_Cys-β-C*H*, Cys-β-C*H*_ = 14.8 Hz), 3.01 (s, 6H, NC*H*), 2.81 (s, 6H, NC*H*), 2.54–2.49 (m, 2H, Cys-β-C*H*), 2.22 (qqd, 2H, Val-β-C*H*,* J*_Val-β-C*H,* Val-γ-C*H*_ = 6.4,* J*_Val-β-C*H,* Val-γ-C*H*_ = 7.2,* J*_Val-β-C*H,* Val-α-C*H*_ = 10.4 Hz), 1.27 (d, 6H, Ala-β-C*H*, *J*_Ala-β-C*H*, Ala-α-C*H*_ = 7.2 Hz), 0.99 (d, 6H, Val-γ-C*H*, *J*_Val-γ-C*H*, Val-β-C*H*_ = 6.4 Hz), 0.76 (d, 6H, Val-γ-C*H*, *J*_Val-γ-C*H*, Val-β-C*H*_ = 7.2 Hz); ^13^C NMR (DMSO-*d*_6_, 100 MHz) δ172.3, 170.2, 169.7, 167.8, 166.0, 152.3, 148.8, 137.1, 131.6, 129.1, 128.4, 127.8, 127.0, 122.3, 64.8, 61.4, 54.1, 53.2, 46.0, 35.3, 30.4, 29.8, 26.5, 20.2, 18.6, 16.6; ESIMS-LR *m*/*z* 1075.4 [(M + Na)^+^]; ESIMS-HR calcd for C_52_H_65_N_10_O_12_S 1053.4499, found 1053.4536; [a]^23^_D_ −208.5 (*c* 0.20, DMSO).

### {N-(Naphthalene-2-carbonyl)-D-Ser-L-Ala-N-Boc-N-Me-L-Cys-N-Me-L-Val}_2_ sulfide (7)

Compound **7** (8.8 mg, 8.4 µmol, 33%) was synthesized using methods previously reported by our group^20^, from 2-naphthoic acid (17.2 mg, 100 µmol) with flash silica gel column chromatography ($\Phi$ 0.7 × 8.4 cm, CHCl_3_/MeOH: 100/0 → 99/1 → 98.5/1.5) as a white amorphous solid. ^1^H NMR (DMSO-*d*_6_, 400 MHz) δ 8.60 (d, 2H, Ser-N*H*, *J*_Ser-N*H*, Ser-α-C*H*_ = 7.8 Hz), 8.44 (s, 2H, Ar–*H*), 8.07–7.98 (m, 6H, Ar–*H*), 7.91 (dd, 2H, Ar–*H*, *J* = 1.6, 8.6 Hz), 7.82 (d, 2H, Ala-N*H*, *J*_Ala-N*H*, Ala-α-C*H*_ = 5.6 Hz), 7.65–7.59 (m, 4H, Ar–*H*), 6.29 (dd, 2H, Cys-α-C*H*, *J*_Cys-α-C*H*, Cys-β-C*H*_ = 6.6,* J*_Cys-α-C*H*, Cys-β-C*H*_ = 6.8 Hz), 4.86 (d, 2H, Val-α-C*H*, *J*_Val-α-C*H*, Val-β-C*H*_ = 10.4 Hz), 4.71 (ddd, 2H, Ser-α-C*H*,* J*_Ser-α-C*H*, Ser-β-C*H*_ = 2.8,* J*_Ser-α-C*H*, Ser-N*H*_ = 7.8, *J*_Ser-α-C*H*, Ser-β-C*H*_ = 8.0 Hz), 4.57 (dd, 2H, Ala-α-C*H*, *J*_Ala-α-C*H*, Ala-β-C*H*_ = *J*_Ala-α-C*H*, Ala-N*H*_ = 6.4 Hz), 4.52–4.47 (m, 2H, Ser-β-C*H*), 4.32 (dd, 2H, Ser-β-C*H*, *J*_Ser-β-C*H*, Ser-α-C*H*_ = 2.8,* J*_Ser-β-C*H*, Ser-β-C*H*_ = 11.0 Hz), 3.10 (dd, 2H, Cys-β-C*H*, *J*_Cys-β-C*H*, Cys-α-C*H*_ = 6.6, *J*_Cys-β-C*H*, Cys-β-C*H*_ = 14.8 Hz), 3.01 (s, 6H, NC*H*), 2.81 (s, 6H, NC*H*), 2.53–2.49 (m, 2H, Cys-β-C*H*), 2.22 (qqd, 2H, Val-β-C*H*,* J*_Val-β-C*H,* Val-γ-C*H*_ = 6.4,* J*_Val-β-C*H,* Val-γ-C*H*_ = 6.8,* J*_Val-β-C*H,* Val-α-C*H*_ = 10.4 Hz), 1.27 (d, 6H, Ala-β-C*H*, *J*_Ala-β-C*H*, Ala-α-C*H*_ = 7.2 Hz), 0.99 (d, 6H, Val-γ-C*H*, *J*_Val-γ-C*H*, Val-β-C*H*_ = 6.8 Hz), 0.76 (d, 6H, Val-γ-C*H*, *J*_Val-γ-C*H*, Val-β-C*H*_ = 6.4 Hz); ^13^C NMR (CDCl_3_, 100 MHz) δ173.2, 173.1, 169.7, 168.3, 167.9, 135.2, 132.7, 129.3, 129.0, 128.5, 128.4, 128.3, 127.9, 127.1, 123.3, 65.7, 61.9, 55.1, 54.9, 46.6, 36.5, 30.7, 30.3, 27.0, 20.5, 18.3, 17.6; ESIMS-LR *m*/*z* 1073.2 [(M + Na)^+^]; ESIMS-HR calcd for C_54_H_67_N_8_O_12_S 1051.4594, found 1051.4565; [α]^24^_D_ −235.0 (*c* 0.20, CHCl_3_).

### (N-Boc-D-Ser-L-Ala-N-Boc-N-Me-L-Cys-N-Me-L-Val)_2_ sulfide (22)

Compounds were prepared using methods previously reported by our group^20^. Namely, compound **21** (20.0 mg, 19.8 µmol) was treated with thioanisole (185 µL, 1.58 mmol) and TFA (3.9 mL) at room temperature, and the whole mixture was stirred at 40 °C for 15 h. The resulting mixture was concentrated *in vacuo*, and washed with Et_2_O (15 mL × 3) to afford a pale yellow solid. A solution of the residue and Boc_2_O (18.2 µL, 79.2 µmol) in THF (0.4 mL) was treated with Et_3_N (21.9 µL, 158 µmol) at room temperature, and the whole mixture was stirred at 40 °C for 5 h. The resulting mixture was diluted with AcOEt (15 mL), and the solution was washed with 1 M *aq*. HCl (5 mL × 2), brine (5 mL), dried (Na_2_SO_4_), filtered, and concentrated *in vacuo*. The residue was purified by silica gel column chromatography ($\Phi$ 1 × 10 cm, CHCl_3_/MeOH: 100/0 → 99/1 → 98/2 → 97/3) to afford **22** (7.3 mg, 7.7 µmol, 39% over 2 steps) as a colorless solid. ^1^H NMR (CDCl_3_, 400 MHz) δ 6.81 (s, 2H, Ala-N*H*), 6.22 (br s, 2H, Cys-α-C*H*), 5.28 (d, 2H, Ser-N*H*, *J*_Ser-NH, Ser-α-CH_ = 6.4 Hz), 5.05 (d, 2H, Val-α-C*H*, *J*_Val-α-CH, Val-β-CH_ = 10.6 Hz), 4.81 (br s, 2H, Ala-α-C*H*), 4.65 (dd, 2H, Ser-β-C*H*, *J*_gem_ = 11.9, *J*_Ser-β-CH, Ser-α-CH_ = 4.1 Hz), 4.53 (d, 2H, Ser-β-C*H*, *J*_gem_ = 11.9 Hz), 4.38 (br s, 2H, Ser-α-C*H*), 3.30 (m, 2H, Cys-β-C*H*), 3.07 (s, 6H, *N*–C*H*), 2.92 (s, 6H, N–C*H*) 2.48 (br s, 2H, Cys-β-C*H*), 2.23 (m, 2H, Val-β-C*H*), 1.47 (s, 18H, Boc), 1.37 (d, 6H, Ala-β-C*H*, *J*_Ala-β-CH, Ala-α-CH_ = 7.3 Hz), 1.080 (d, 6H, Val-γ-C*H*, *J*_Val-γ-CH, Val-β-CH_ = 6.4 Hz), 0.82 (d, 6H, Val-γ-C*H*, *J*_Val-γ-CH, Val-β-CH_ = 6.9 Hz); ^13^C NMR (CDCl_3_, 100 MHz) δ 173.2, 171.6, 169.9, 168.4, 155.6, 81.5, 64.3, 62.1, 55.0, 54.8, 46.1, 36.2, 31.4, 30.3, 28.3, 27.5, 20.4, 18.9, 17.7; ESIMS-LR *m/z* 943.87 [(M + H)^+^]; ESIMS-HR calcd for C_42_H_71_N_8_O_14_S 943.4805; found 943.4833; [α]^17^_D_ − 187.33 (_*C*_ 0.73, CHCl_3_).

### General procedure for the synthesis of 8–20

Compounds were prepared using methods previously reported by our group^20^. Namely, compound **22** (100 mg, 106 µmol) was treated with 4 M HCl/1,4-dioxane (8 mL) at room temperature for 1 h. The resulting mixture was concentrated *in vacuo*, and the residue was washed with Et_2_O (15 mL × 3) to afford a white solid. The residue was divided into 5 portions. A suspension of each portion, carboxylic acid (63.6 µmol), HOAt (8.7 mg, 63.6 µmol) and Et_3_N (20.6 µL, 148 µmol) in DMF (0.3 mL) was treated with EDCI (12.2 mg, 63.6 µmol) at 0 °C, and the whole mixture was stirred at room temperature for several hours. The resulting mixture was diluted with AcOEt, and the solution was washed with H_2_O, 1 M *aq*. HCl, *sat. aq*. NaHCO_3_ and, brine, dried over Na_2_SO_4_, filtered, and concentrated *in vacuo*. The residue was purified to afford **8**–**20**.

### {N-(Indol-2-carbonyl)-D-Ser-L-Ala-N-Boc-N-Me-L-Cys-N-Me-L-Val}_2_ sulfide (8)

Compound **8** (6.5 mg, 6.3 µmol, 30% over 2 steps) was synthesized using methods previously reported by our group^20^, from indole-2-carboxylic acid (10.2 mg, 63.6 µmol), and purified with high flash silica gel column chromatography ($\Phi$ 2 × 6 cm, CHCl_3_/MeOH: 100/0 → 94/6) as a white solid. ^1^H NMR (DMSO-*d*_6_, 400 MHz) δ 11.7 (d, 2H, Ar–N*H*), 8.40 (d, 2H, Ser-N*H*, *J*_Ser-NH, Ser-α-CH_ = 7.3 Hz), 7.84 (d, 2H, Ala-N*H*, *J*_Ala-NH, Ala-α-CH_ = 4.1 Hz), 7.65 (d, 2H, Ar, *J* = 7.8 Hz), 7.44 (d, 2H, Ar, *J* = 7.8 Hz), 7.21–7.04 (m, 6H, Ar), 6.28 (t, 2H, Cys-α-C*H*, *J*_Cys-α-CH, Cys-β-CH_ = 6.0 Hz), 4.83 (d, 2H, Val-α-C*H*, *J*_Val-α-CH, Val-β-CH_ = 8.7 Hz), 4.65 (m, 2H, Ser-α-C*H*), 4.55 (qd, 2H, Ala-α-C*H*, *J*_Ala-α-CH, Ala-NH_ = 5.0, *J*_Ala-α-CH, Ala-β-CH_ = 4.1 Hz), 4.45 (m, 2H, Ser-β-C*H*), 4.31 (d, 2H, Ser-β-C*H*, *J*_gem_ = 11.0 Hz), 3.08 (dd, 2H, Cys-β-C*H*, *J*_gem_ = 14.6, *J*_Cys-β-CH, Cys-α-CH_ = 5.5 Hz), 3.03 (s, 6H, *N*–C*H*), 2.80 (s, 6H, *N*–C*H*), 2.50–2.45 (overlap, 2H, Cys-β-C*H*), 2.23 (m, 2H, Val-β-C*H*), 1.27 (d, 6H, Ala-β-C*H*, *J*_Ala-β-CH, Ala-α-CH_ = 5.0 Hz), 0.99 (d, 6H, Val-γ-C*H*, *J*_Val-γ-CH, Val-β-CH_ = 4.6 Hz), 0.76 (d, 6H, Val-γ-C*H*, *J*_Val-γ-CH, Val-β-CH_ = 5.0 Hz); ^13^C NMR (DMSO-*d*_6_, 100 MHz) δ 172.3, 170.3, 169.8, 167.8, 161.2, 136.6, 130.8, 126.9, 123.8, 121.7, 119.9, 112.4, 103.7, 64.8, 61.5, 54.2, 52.7, 46.0, 30.5, 29.8, 29.0, 26.6, 20.3, 18.8, 16.6; ESIMS-LR *m/z* 1029.53 [(M + H)^+^]; ESIMS-HR calcd for C_50_H_65_N_10_O_12_S 1029.4499, found 1029.4497; [α]^22^_D_ − 177.67 (_*C*_ 1.08, DMSO).

### {N-(Indol-3-carbonyl)-D-Ser-L-Ala-N-Boc-N-Me-L-Cys-N-Me-L-Val}_2_ sulfide (9)

Compound **9** (4.2 mg, 4.1 µmol, 19% over 3 steps) was synthesized using methods previously reported by our group^20^, from *N*-Boc-indole-3-carboxylic acid (16.6 mg, 63.6 µmol). After amide coupling, the residue was treated with 50% TFA/CH_2_Cl_2_ (1 mL) at room temperature, and the whole mixture was stirred at 40 °C for 5 h. The resulting mixture was concentrated *in vacuo*, and the residue was purified by high flash silica gel column chromatography ($\Phi$ 2 × 6 cm, CHCl_3_/MeOH: 100/0 → 94/6) to afford compound **9** as a white solid. ^1^H NMR (DMSO-*d*_6_, 400 MHz) δ 11.7 (d, 2H, Ar–N*H*), 8.06 (d, 2H, Ar, *J* = 8.2 Hz), 8.05 (s, 2H, Ar-C^2^*H*), 7.82 (d, 2H, Ser-N*H*, *J*_Ser-NH, Ser-α-CH_ = 7.3 Hz), 7.78 (d, 2H, Ala-N*H*, *J*_Ala-NH, Ala-α-CH_ = 5.0 Hz), 7.18–7.11 (m, 4H, Ar), 6.28 (dd, 2H, Cys-α-C*H*, *J*_Cys-α-CH, Cys-β-CH_ = 6.9, 5.9 Hz), 4.81 (d, 2H, Val-α-C*H*, *J*_Val-α-CH, Val-β-CH_ = 10.5 Hz), 4.69 (m, 2H, Ser-α-C*H*), 4.57 (qd, 2H, Ala-α-C*H*, *J*_Ala-α-CH, Ala-β-CH_ = 6.0, *J*_Ala-α-CH, Ala-NH_ = 5.0 Hz), 4.43 (m, 2H, Ser-β-C*H*), 4.30 (d, 2H, Ser-β-C*H*, *J*_gem_ = 10.5 Hz), 3.08 (dd, 2H, Cys-β-C*H*, *J*_gem_ = 14.6, *J*_Cys-β-CH, Cys-α-CH_ = 5.9 Hz), 3.02 (s, 6H, *N*–C*H*), 2.80 (s, 6H, *N*–C*H*), 2.50–2.45 (dd, 2H, Cys-β-C*H*, *J*_gem_ = 14.6, *J*_Cys-β-CH, Cys-α-CH_ = 6.9 Hz), 2.21 (m, 2H, Val-β-C*H*), 1.26 (d, 6H, Ala-β-C*H*, *J*_Ala-β-CH, Ala-α-CH_ = 6.4 Hz), 0.99 (d, 6H, Val-γ-C*H*, *J*_Val-γ-CH, Val-β-CH_ = 5.5 Hz), 0.76 (d, 6H, Val-γ-C*H*, *J*_Val-γ-CH, Val-β-CH_ = 6.0 Hz); ^13^C NMR (DMSO-*d*_6_, 100 MHz) δ 172.3, 170.3, 169.9, 168.5, 164.5, 136.2, 129.0, 125.8, 122.1, 120.6, 112.1, 109.8, 65.1, 61.6, 54.2, 52.4, 46.0, 35.3, 30.5, 29.7, 26.6, 20.2, 18.9, 16.7; ESIMS-LR *m/z* 1029.88 [(M + H)^+^]; ESIMS-HR calcd for C_50_H_65_N_10_O_12_S_1_ 1029.4499, found 1029.4497; [α]^23^_D_ − 113.26 (_*C*_ 0.19, DMSO).

### {N-(Indol-5-carbonyl)-D-Ser-L-Ala-N-Boc-N-Me-L-Cys-N-Me-L-Val}_2_ sulfide (10)

Compound **10** (12.4 mg, 12.1 µmol, 57% over 2 steps) was synthesized using methods previously reported by our group^20^, from indole-5-carboxylic acid (10.2 mg, 63.6 µmol), and purified with high flash silica gel column chromatography ($\Phi$ 2 × 6 cm, CHCl_3_/MeOH: 100/0 → 94/6) as a white solid. ^1^H NMR (DMSO-*d*_6_, 400 MHz) δ 11.4 (d, 2H, Ar–N*H*), 8.29 (d, 2H, Ser-N*H*, *J*_Ser-NH, Ser-α-CH_ = 7.3 Hz), 8.12 (s, 2H, Ar), 7.79 (d, 2H, Ala-N*H*, *J*_Ala-NH, Ala-α-CH_ = 5.0 Hz), 7.61 (d, 2H, Ar, *J* = 8.7 Hz), 7.46 (d, 2H, Ar, J = 8.7 Hz), 7.45 (s, 2H, Ar), 6.56 (s, 2H, Ar), 6.27 (dd, 2H, Cys-α-C*H*, *J*_Cys-α-CH, Cys-β-CH_ = 6.6, 6.2 Hz), 4.83 (d, 2H, Val-α-C*H*, *J*_Val-α-CH, Val-β-CH_ = 10.5 Hz), 4.66 (m, 2H, Ser-α-C*H*), 4.56 (qd, 2H, Ala-α-C*H*, *J*_Ala-α-CH, Ala-β-CH_ = 6.9, *J*_Ala-α-CH, Ala-NH_ = 5.0 Hz), 4.44 (m, 2H, Ser-β-C*H*), 4.28 (d, 2H, Ser-β-C*H*, *J*_gem_ = 10.6 Hz), 3.09 (dd, 2H, Cys-β-C*H*, *J*_gem_ = 15.1, *J*_Cys-β-CH, Cys-α-CH_ = 6.2 Hz), 3.01 (s, 6H, *N*–C*H*), 2.80 (s, 6H, *N*–C*H*), 2.50 (overlap, 2H, Cys-β-C*H*), 2.24–2.18 (m, 2H, Val-β-C*H*), 1.26 (d, 6H, Ala-β-C*H*, *J*_Ala-β-CH, Ala-α-CH_ = 6.9 Hz), 0.98 (d, 6H, Val-γ-C*H*, *J*_Val-γ-CH, Val-β-CH_ = 6.4 Hz), 0.76 (d, 6H, Val-γ-C*H*, *J*_Val-γ-CH, Val-β-CH_ = 6.4 Hz); ^13^C NMR (DMSO-*d*_6_, 100 MHz) δ 172.3, 170.3, 169.8, 168.3, 167.5, 136.7, 127.0, 124.6, 120.7, 120.3, 111.1, 102.2, 65.0, 61.5, 54.2, 53.1, 45.9, 35.3, 30.5, 29.8, 26.6, 20.2, 18.8, 16.7; ESIMS-LR *m/z* 1051.95 [(M + Na)^+^]; ESIMS-HR calcd for C_50_H_65_N_10_O_12_S 1029.4499, found 1029.4497; [α]^24^_D_ − 234.83 (_*C*_ 0.81, DMSO).

### {N-(Indol-6-carbonyl)-D-Ser-L-Ala-N-Boc-N-Me-L-Cys-N-Me-L-Val}_2_ sulfide (11)

Compound **11** (6.1 mg, 5.9 µmol, 28% over 2 steps) was synthesized using methods previously reported by our group^20^, from indole-6-carboxylic acid (10.2 mg, 63.6 µmol), and purified with high flash silica gel column chromatography ($\Phi$ 2 × 6 cm, CHCl_3_/MeOH: 100/0 → 94/6) as a white solid. ^1^H NMR (DMSO-*d*_6_, 400 MHz) δ 11.5 (d, 2H, Ar–N*H*), 8.30 (d, 2H, Ser-N*H*, *J*_Ser-NH, Ser-α-CH_ = 7.4 Hz), 7.92 (s, 2H, Ar), 7.78 (d, 2H, Ala-N*H*, *J*_Ala-NH, Ala-α-CH_ = 5.5 Hz), 7.62 (d, 2H, Ar *J* = 8.5 Hz), 7.54 (s, 2H, Ar), 7.49 (d, 2H, *J* = 8.5 Hz), 6.50 (s, 2H, Ar), 6.28 (t, 2H, Cys-α-C*H*, *J*_Cys-α-CH, Cys-β-CH_ = 6.4 Hz), 4.83 (d, 2H, Val-α-C*H*, *J*_Val-α-CH, Val-β-CH_ = 10.1 Hz), 4.66 (m, 2H, Ser-α-C*H*), 4.57 (qd, 2H, Ala-α-C*H*, *J*_Ala-α-CH, Ala-β-CH_ = 7.3, *J*_Ala-α-CH, Ala-NH_ = 5.5 Hz), 4.45 (m, 2H, Ser-β-C*H*), 4.28 (d, 2H, Ser-β-C*H*, *J*_gem_ = 10.1 Hz), 3.08 (dd, 2H, Cys-β-C*H*, *J*_gem_ = 15.1, *J*_Cys-β-CH, Cys-α-CH_ = 6.4 Hz), 3.01 (s, 6H, *N*–C*H*), 2.79 (s, 6H, *N*–C*H*), 2.50 (overlap, 2H, Cys-β-C*H*), 2.20 (m, 2H, Val-β-C*H*), 1.25 (d, 6H, Ala-β-C*H*,* J*_Ala-β-CH, Ala-α-CH_ = 7.3 Hz), 0.98 (d, 6H, Val-γ-C*H*, *J*_Val-γ-CH, Val-β-CH_ = 6.0 Hz), 0.75 (d, 6H, Val-γ-C*H*, *J*_Val-γ-CH, Val-β-CH_ = 6.4 Hz); ^13^C NMR (DMSO-*d*_6_, 100 MHz) δ 172.3, 170.3, 169.8, 168.2, 167.4, 135.1, 130.2, 128.4, 126.3, 119.6, 118.2, 111.6, 101.4, 64.9, 61.5, 54.2, 53.2, 45.9, 35.4, 30.5, 29.8, 26.6, 20.3, 18.8, 16.7; ESIMS-LR *m/z* 1051.91 [(M + Na)^+^]; ESIMS-HR calcd for C_50_H_65_N_10_O_12_S 1029.4499, found 1029.4497; [α]^24^_D_ − 216.52 (_*C*_ 0.26, DMSO).

### {N-(Indol-7-carbonyl)-D-Ser-L-Ala-N-Boc-N-Me-L-Cys-N-Me-L-Val}_2_ sulfide (**12**)

Compound **12** (6.1 mg, 5.9 µmol, 28% over 2 steps) was synthesized using methods previously reported by our group^20^, from indole-7-carboxylic acid (10.2 mg, 63.6 µmol), and purified with high flash silica gel column chromatography ($\Phi$ 2 × 6 cm, CHCl_3_/MeOH: 100/0 → 94/6) as a white solid. ^1^H NMR (DMSO-*d*_6_, 400 MHz) δ 11.2 (d, 2H, Ar–N*H*), 8.45 (d, 2H, Ser-N*H*, *J*_Ser-NH, Ser-α-CH_ = 7.3 Hz), 7.84 (d, 2H, Ala-N*H*, *J*_Ala-NH, Ala-α-CH_ = 5.9 Hz), 7.78 (d, 2H, Ar *J* = 8.2 Hz), 7.62 (d, 2H, Ar, *J* = 7.3 Hz), 7.36 (t, 2H, *J* = 2.7 Hz), 7.12 (t, 2H, *J* = 7.6 Hz), 6.51 (dd, 2H, Ar, J = 3.2, 2.3 Hz), 6.27 (dd, 2H, Cys-α-C*H*, *J*_Cys-α-CH, Cys-β-CH_ = 6.9, 6.4 Hz), 4.84 (d, 2H, Val-α-C*H*, *J*_Val-α-CH, Val-β-CH_ = 10.5 Hz), 4.72 (ddd, 2H, Ser-α-C*H, J*_Ser-α-CH, Ser-β-CH_ = 8.2, *J*_Ser-α-CH, Ser-NH_ = 7.3, *J*_Ser-α-CH, Ser-β-CH_ = 2.6 Hz), 4.56 (qd, 2H, Ala-α-C*H*, *J*_Ala-α-CH, Ala-β-CH_ = 6.8, *J*_Ala-α-CH, Ala-NH_ = 5.9 Hz), 4.47 (dd, 2H, Ser-β-C*H, J*_gem_ = 11.2, *J*_Ser-β-CH, Ser-α-CH_ = 8.2 Hz), 4.34 (dd, 2H, Ser-β-C*H, J*_gem_ = 11.2, *J*_Ser-β-CH, Ser-α-CH_ = 2.6 Hz), 3.09 (dd, 2H, Cys-β-C*H*, *J*_gem_ = 14.6, *J*_Cys-β-CH, Cys-α-CH_ = 6.4 Hz), 3.01 (s, 6H, *N*–C*H*), 2.79 (s, 6H, *N*–C*H*), 2.50 (overlap, 2H, Cys-β-C*H*), 2.21 (m, 2H, Val-β-C*H*), 1.25 (d, 6H, Ala-β-C*H*, *J*_Ala-β-CH, Ala-α-CH_ = 6.8 Hz), 0.98 (d, 6H, Val-γ-C*H*, *J*_Val-γ-CH, Val-β-CH_ = 6.4 Hz), 0.75 (d, 6H, Val-γ-C*H*, *J*_Val-γ-CH, Val-β-CH_ = 6.9 Hz); ^13^C NMR (DMSO-*d*_6_, 100 MHz) δ 172.3, 170.4, 169.8, 168.1, 167.0, 134.1, 129.3, 127.0, 124.4, 120.4, 118.1, 116.2, 101.2, 64.9, 61.5, 54.2, 52.9, 46.0, 35.4, 30.5, 29.8, 26.6, 20.3, 18.7, 16.7; ESIMS-LR *m/z* 1051.71 [(M + Na)^+^]; ESIMS-HR calcd for C_50_H_65_N_10_O_12_S 1029.4499, found 1029.4497; [α]^25^_D_ − 207.55 (_*C*_ 0.61, DMSO).

### {N-(Benzofurane-2-carbonyl)-D-Ser-L-Ala-N-Boc-N-Me-L-Cys-N-Me-L-Val}_2_ sulfide (**13**)

Compound **13** (18.5 mg, 18.0 µmol, 85% over 2 steps) was synthesized using methods previously reported by our group^20^, from benzofuran-2-carboxylic acid (10.3 mg, 63.6 µmol), and purified with silica gel column chromatography ($\Phi$ 1 × 10 cm, CHCl_3_/MeOH: 100/0 → 99/1 → 98/2 → 97/3) as a white solid. ^1^H NMR (DMSO-*d*_6_, 400 MHz) δ 8.38 (d, 2H, Ser-N*H*, *J*_Ser-NH, Ser-α-CH_ = 8.7 Hz), 7.98 (d, 2H, Ala-N*H*, *J*_Ala-NH, Ala-α-CH_ = 6.0 Hz), 7.80 (d, 2H, Ar, *J* = 8.2 Hz), 7.67 (d, 2H, Ar, *J* = 8.2 Hz), 7.64 (s, 2H, Ar), 7.49 (m, 2H,r Ar), 7.35 (m, 2H, Ar), 6.28 (dd, 2H, Cys-α-C*H*, *J*_Cys-α-CH, Cys-β-CH_ = 6.9, 6.4 Hz), 4.84 (d, 2H, Val-α-C*H*, *J*_Val-α-CH, Val-β-CH_ = 10.5 Hz), 4.69 (m, 2H, Ser-α-C*H*), 4.57 (qd, 2H, Ala-α-C*H*, *J*_Ala-α-CH, Ala-β-CH_ = 6.9, *J*_Ala-α-CH, Ala-NH_ = 6.0 Hz), 4.46 (dd, 2H, Ser-β-C*H*, *J*_gem_ = 11.4, *J*_Cys-β-CH, Cys-α-CH_ = 7.8 Hz), 4.31 (d, 2H, Ser-β-C*H, J*_gem_ = 11.4 Hz), 3.09 (dd, 2H, Cys-β-C*H*, *J*_gem_ = 15.1, *J*_Cys-β-CH, Cys-α-CH_ = 6.4 Hz), 3.03 (s, 6H, *N*–C*H*), 2.80 (s, 6H, *N*–C*H*), 2.46 (overlap, 2H, Cys-β-C*H*), 2.20 (m, 2H, Val-β-C*H*), 1.28 (d, 6H, Ala-β-C*H*, *J*_Ala-β-CH, Ala-α-CH_ = 6.9 Hz), 0.98 (d, 6H, Val-γ-C*H*, *J*_Val-γ-CH, Val-β-CH_ = 6.9 Hz), 0.77 (d, 6H, Val-γ-C*H*, *J*_Val-γ-CH, Val-β-CH_ = 6.9 Hz); ^13^C NMR (DMSO-*d*_6_, 100 MHz) δ 172.3, 170.3, 169.8, 168.1, 167.0, 134.1, 129.3, 126.9, 124.4, 120.4, 118.1, 116.2, 101.2, 64.9, 61.5, 54.2, 52.9, 46.0, 35.4, 30.5, 29.8, 26.6, 20.2, 18.7, 16.7; ESIMS-LR *m/z* 1053.41 [(M + Na)^+^]; ESIMS-HR calcd for C_50_H_63_N_8_O_14_S 1031.4179, found 1031.4124; [α]^25^_D_ − 238.12 (_*C*_ 0.97, DMSO).

### {N-(Benzothiophene-2-carbonyl)-D-Ser-L-Ala-N-Boc-N-Me-L-Cys-N-Me-L-Val}_2_ sulfide (**14**)

Compound **14** (19.6 mg, 18.5 µmol, 87% over 2 steps) was synthesized using methods previously reported by our group^20^, from benzothiophene-2-carboxylic acid (11.3 mg, 63.6 µmol), and purified with silica gel column chromatography ($\Phi$ 1 × 10 cm, CHCl_3_/MeOH: 100/0 → 99/1 → 98/2 → 97/3) as a white solid. ^1^H NMR (DMSO-*d*_6_, 400 MHz) δ 8.71 (d, 2H, Ser-N*H*, *J*_Ser-NH, Ser-α-CH_ = 7.8 Hz), 8.14 (s, 2H, Ar), 8.06–8.00 (m, 4H, Ar), 7.85 (d, 2H, Ala-N*H*, *J*_Ala-NH, Ala-α-CH_ = 5.5 Hz), 7.51–7.44 (m, 4H, Ar), 6.29 (t, 2H, Cys-α-C*H*, *J*_Cys-α-CH, Cys-β-CH_ = 6.4 Hz), 4.84 (d, 2H, Val-α-C*H*, *J*_Val-α-CH, Val-β-CH_ = 10.5 Hz), 4.64 (m, 2H, Ser-α-C*H*), 4.56 (dq, 2H, Ala-α-C*H*, *J*_Ala-α-CH, Ala-β-CH_ = 6.9, *J*_Ala-α-CH, Ala-NH_ = 5.5 Hz), 4.50 (m, 2H, Ser-β-C*H*), 4.31 (m, 2H, Ser-β-C*H*), 3.08 (dd, 2H, Cys-β-C*H*, *J*_gem_ = 15.1, *J*_Cys-β-CH, Cys-α-CH_ = 6.4 Hz), 3.02 (s, 6H, *N*–C*H*), 2.80 (s, 6H, *N*–C*H*), 2.50 (overlap, 2H, Cys-β-C*H*), 2.22 (m, 2H, Val-β-C*H*), 1.28 (d, 6H, Ala-β-C*H*, *J*_Ala-β-CH, Ala-α-CH_ = 6.9 Hz), 0.99 (d, 6H, Val-γ-C*H*, *J*_Val-γ-CH, Val-β-CH_ = 6.4 Hz), 0.75 (d, 6H, Val-γ-C*H*, *J*_Val-γ-CH, Val-β-CH_ = 6.4 Hz); ^13^C NMR (DMSO-*d*_6_, 100 MHz) δ 172.3, 170.2, 169.8, 161.7, 140.3, 138.9, 138.6, 128.4, 126.5, 126.0, 125.4, 125.1, 122.9, 64.6, 61.5, 54.2, 53.1, 46.1, 35.4, 30.5, 29.8, 26.6, 20.3, 18.8, 16.6; ESIMS-LR *m/z* 1085.44 [(M + Na)^+^]; ESIMS-HR calcd for C_50_H_63_N_8_O_12_S_3_ 1063.3722, found 1063.3690; [α]^26^_D_ − 246.77 (_*C*_ 0.58, DMSO).

### {N-(Benzoxazole-5-carbonyl)-D-Ser-L-Ala-N-Boc-N-Me-L-Cys-N-Me-L-Val}_2_ sulfide (**15**)

Compound **15** (7.2 mg, 7.0 µmol, 33% over 2 steps) was synthesized using methods previously reported by our group^20^, from benzoxazole-5-carboxylic acid (10.4 mg, 63.6 µmol), and purified with high flash silica gel column chromatography ($\Phi$ 2 × 6 cm, CHCl_3_/MeOH: 100/0 → 99/1 → 98/2 → 97/3) as a white solid. ^1^H NMR (DMSO-*d*_6_, 400 MHz) δ 8.88 (d, 2H, Ar, *J* = 2.1 Hz), 8.57 (d, 2H, Ser-N*H*, *J*_Ser-NH, Ser-α-CH_ = 6.9 Hz), 8.33 (s, 2H, Ar), 7.96 (d, 2H, Ar, *J* = 8.7 Hz), 7.92 (d, 2H, Ar, *J* = 8.7, 2.1 Hz), 7.81 (d, 2H, Ala-N*H*, *J*_Ala-NH, Ala-α-CH_ = 4.6 Hz), 6.27 (dd, 2H, Cys-α-C*H*, *J*_Cys-α-CH, Cys-β’-CH_ = 6.0, *J*_Cys-α-CH, Cys-β-CH_ = 5.7 Hz), 4.82 (d, 2H, Val-α-C*H*, *J*_Val-α-CH, Val-β-CH_ = 9.2 Hz), 4.65 (m, 2H, Ser-α-C*H*), 4.56 (qd, 2H, Ala-α-C*H*, *J*_Ala-α-CH, Ala-β-CH_ = 5.5, *J*_Ala-α-CH, Ala-NH_ = 4.6 Hz), 4.44 (m, 2H, Ser-β-C*H*), 4.32 (d, 2H, Ser-β-C*H*, *J*_gem_ = 11.0 Hz), 3.08 (dd, 2H, Cys-β-C*H*, *J*_gem_ = 15.1, *J*_Cys-β-CH, Cys-α-CH_ = 5.7 Hz), 3.02 (s, 6H, *N*–C*H*), 2.80 (s, 6H, *N*–C*H*), 2.50 (overlap, 2H, Cys-β-C*H*), 2.19 (m, 2H, Val-β-C*H*), 1.26 (d, 6H, Ala-β-C*H*, *J*_Ala-β-CH, Ala-α-CH_ = 5.5 Hz), 0.99 (d, 6H, Val-γ-C*H*, *J*_Val-γ-CH, Val-β-CH_ = 5.0 Hz), 0.75 (d, 6H, Val-γ-C*H*, *J*_Val-γ-CH, Val-β-CH_ = 5.5 Hz); ^13^C NMR (DMSO-*d*_6_, 100 MHz) δ 172.3, 170.3, 169.8, 167.9, 166.1, 155.6, 151.3, 139.5, 130.8, 125.8, 119.7, 111.2, 64.8, 61.5, 54.1, 53.3, 46.0, 35.3, 30.5, 29.8, 26.5, 20.3, 18.7, 16.6; ESIMS-LR *m/z* 1033.57 [(M + H)^+^]; ESIMS-HR calcd for C_48_H_61_N_10_O_14_S 1033.4084, found 1033.4098; [α]^26^_D_ − 412.43 (_*C*_ 0.21, DMSO).

### {N-(Benzoxazole-6-carbonyl)-D-Ser-L-Ala-N-Boc-N-Me-L-Cys-N-Me-L-Val}_2_ sulfide (**16**)

Compound **16** (11.9 mg, 11.5 µmol, 54% over 2 steps) was synthesized using methods previously reported by our group^20^, from benzoxazole-6-carboxylic acid (10.4 mg, 63.6 µmol), and purified with high flash silica gel column chromatography ($\Phi$ 2 × 6 cm, CHCl_3_/MeOH: 100/0 → 99/1 → 98/2 → 97/3) as a white solid. ^1^H NMR (CD_3_OD, 400 MHz) δ 8.63 (s, 2H, Ar), 8.25 (s, 2H, Ar), 7.98 (dd, 2H, Ar, *J* = 8.2, 1.8 Hz), 7.87 (d, 2H, Ar, *J* = 8.2 Hz), 6.24 (dd, 2H, Cys-α-C*H*, *J*_Cys-α-CH, Cys-β-CH_ = 7.3, 6.4 Hz), 4.93–4.89 (overlap, 4H, Val-α-C*H*, Ser-α-C*H*), 4.70 (q, 2H, Ala-α-C*H*, *J*_Ala-α-CH, Ala-β-CH_ = 7.3 Hz), 4.63 (dd, 2H, Ser-β-C*H*, *J*_gem_ = 11.9, *J*_Ser-β-CH, Ser-α-CH_ = 5.4 Hz), 4.54 (dd, 2H, Ser-β-C*H*, *J*_gem_ = 11.9, *J*_Ser-β-CH, Ser-α-CH_ = 2.3 Hz), 3.34 (dd, 2H, Cys-β-C*H*, *J*_gem_ = 15.1, *J*_Cys-β-CH, Cys-α-CH_ = 6.4 Hz), 3.16 (s, 6H, *N*–C*H*), 3.01 (s, 6H, *N*–C*H*), 2.65 (dd, 2H, Cys-β-C*H*, *J*_gem_ = 15.1, *J*_Cys-β-CH, Cys-α-CH_ = 7.3 Hz), 2.25 (m, 2H, Val-β-C*H*), 1.39 (d, 6H, Ala-β-C*H*, *J*_Ala-β-CH, Ala-α-CH_ = 7.3 Hz), 1.04 (d, 6H, Val-γ-C*H*, *J*_Val-γ-CH, Val-β-CH_ = 6.4 Hz), 0.84 (d, 6H, Val-γ-C*H*, *J*_Val-γ-CH, Val-β-CH_ = 6.9 Hz); ^13^C NMR (CD_3_OD, 100 MHz) δ 175.3, 172.2, 171.9, 170.2, 169.5, 157.4, 151.1, 143.9, 133.1, 125.7, 121.0, 112.0, 65.4, 63.9, 56.1, 54.8, 35.6, 32.2, 30.8, 28.4, 20.5, 19.6, 16.6, 7.9; ESIMS-LR *m/z* 1033.63 [(M + H)^+^]; ESIMS-HR calcd for C_48_H_61_N_10_O_14_S 1033.4084, found 1033.4098; [α]^24^_D_ − 257.59 (_*C*_ 0.45, DMSO).

### {N-(Benzthiazole-6-carbonyl)-D-Ser-L-Ala-N-Boc-N-Me-L-Cys-N-Me-L-Val}_2_ sulfide (**17**)

Compound **17** (11.5 mg, 10.8 µmol, 51% over 2 steps) was synthesized using methods previously reported by our group^20^, from benzothiazole-6-carboxylic acid (11.4 mg, 63.6 µmol), and purified with high flash silica gel column chromatography ($\Phi$ 2 × 6 cm, CHCl_3_/MeOH: 100/0 → 99/1 → 98/2 → 97/3) as a white solid. ^1^H NMR (DMSO-*d*_6_, 400 MHz) δ 9.56 (s, 2H, Ar), 8.69 (d, 2H, Ar, *J* = 1.4 Hz), 8.62 (d, 2H, Ser-N*H*, *J*_Ser-NH, Ser-α-CH_ = 8.2 Hz), 8.20 (d, 2H, Ar, *J* = 8.7 Hz), 8.00 (d, 2H, Ar, *J* = 8.7, 1.4 Hz), 7.84 (d, 2H, Ala-N*H*, *J*_Ala-NH, Ala-α-CH_ = 5.5 Hz), 6.27 (dd, 2H, Cys-α-C*H*, *J*_Cys-α-CH, Cys-β-CH_ = 6.9, 6.4 Hz), 4.84 (d, 2H, Val-α-C*H*, *J*_Val-α-CH, Val-β-CH_ = 10.6 Hz), 4.66 (ddd, 2H, Ser-α-C*H*, *J*_Ser-α-CH, Ser-β-CH_ = 9.2, *J*_Ser-α-CH, Ser-NH_ = 8.2, *J*_Ser-α-CH, Ser-β-CH_ = 2.8 Hz), 4.55 (qd, 2H, Ala-α-C*H*, *J*_Ala-α-CH, Ala-β-CH_ = 6.9, *J*_Ala-α-CH, Ala-NH_ = 5.5 Hz), 4.45 (dd, 2H, Ser-β-C*H*, *J*_gem_ = 11.4, *J*_Ser-β-CH, Ser-α-CH,_ = 9.2 Hz), 4.31 (dd, 2H, Ser-β-C*H*, *J*_gem_ = 11.4, *J*_Ser-β-CH, Ser-α-CH,_ = 2.8 Hz), 3.08 (dd, 2H, Cys-β-C*H*, *J*_gem_ = 15.1, *J*_Cys-β-CH, Cys-α-CH_ = 6.4 Hz), 3.00 (s, 6H, *N*–C*H*), 2.80 (s, 6H, *N*–C*H*), 2.54–48(overlap, 2H, Cys-β-C*H*), 2.24–2.17 (m, 2H, Val-β-C*H*), 1.26 (d, 6H, Ala-β-C*H*, *J*_Ala-β-CH, Ala-α-CH_ = 6.9 Hz), 0.99 (d, 6H, Val-γ-C*H*, *J*_Val-γ-CH, Val-β-CH_ = 6.4 Hz), 0.75 (d, 6H, Val-γ-C*H*, *J*_Val-γ-CH, Val-β-CH_ = 6.4 Hz); ^13^C NMR (DMSO-*d*_6_, 100 MHz) δ 172.3, 170.3, 169.8, 167.9, 166.1, 159.3, 154.9, 133.7, 131.0, 125.6, 122.8, 122.6, 64.8, 61.4, 54.1, 53.2, 46.1, 35.3, 30.5, 29.8, 26.5, 20.3, 18.7, 16.6; ESIMS-LR *m/z* 1065.53 [(M + H)^+^]; ESIMS-HR calcd for C_48_H_61_N_10_O_12_S_3_ 1065.3627, found 1065.3638; [α]^25^_D_ − 224.92 (_*C*_ 0.95, DMSO).

### {N-(4-Benzoylphenylcarbonyl)-D-Ser-L-Ala-N-Boc-N-Me-L-Cys-N-Me-L-Val}_2_ sulfide (**18**)

Compound **18** (17.4 mg, 15.0 µmol, 71% over 2 steps) was synthesized using methods previously reported by our group^20^, from *p*-benzoylbenzoic acid (14.4 mg, 63.6 µmol), and purified with high flash silica gel column chromatography ($\Phi$ 2 × 6 cm, CHCl_3_/MeOH: 100/0 → 99/1 → 98/2) as a white solid. ^1^H NMR (DMSO-*d*_6_, 400 MHz) δ 8.62 (d, 2H, Ser-N*H*, *J*_Ser-NH, Ser-α-CH_ = 7.8 Hz), 7.99 (d, 4H, Ar, *J* = 8.2 Hz), 7.84 (d, 4H, Ar, *J* = 8.2 Hz), 7.82 (m, 2H, Ala-N*H*), 7.77–7.69 (m, 6H, Ar), 7.60–7.57 (m, 4H, Ar), 6.29 (t, 2H, Cys-α-C*H*, *J*_Cys-α-CH, Cys-β-CH_ = 6.4 Hz), 4.83 (d, 2H, Val-α-C*H*, *J*_Val-α-CH, Val-β-CH_ = 10.6 Hz), 4.66 (m, 2H, Ser-α-C*H*), 4.56 (m, 2H, Ala-α-C*H*), 4.45 (m, 2H, Ser-β-C*H*), 4.31 (d, 2H, Ser-β-C*H*, *J*_gem_ = 11.0 Hz), 3.08 (dd, 2H, Cys-β-C*H*, *J*_gem_ = 14.6, *J*_Cys-β-CH, Cys-α-CH_ = 6.4 Hz), 3.00 (s, 6H, *N*–C*H*), 2.79 (s, 6H, *N*–C*H*), 2.50 (overlap, 2H, Cys-β-C*H*), 2.22 (m, 2H, Val-β-C*H*), 1.25 (d, 6H, Ala-β-C*H*, *J*_Ala-β-CH, Ala-α-CH_ = 6.9 Hz), 0.99 (d, 6H, Val-γ-C*H*, *J*_Val-γ-CH, Val-β-CH_ = 6.4 Hz), 0.75 (d, 6H, Val-γ-C*H*, *J*_Val-γ-CH, Val-β-CH_ = 6.4 Hz); ^13^C NMR (DMSO-*d*_6_, 100 MHz) δ 195.4, 172.3, 170.3, 169.8, 167.7, 165.9, 139.6, 137.1, 136.6, 133.1, 129.7, 129.6, 128.7, 127.8, 64.7, 61.4, 54.1, 53.2, 46.0, 35.3, 30.5, 29.8, 26.5, 20.3, 18.7, 16.6; ESIMS-LR *m/z* 1181.50 [(M + Na)^+^]; ESIMS-HR calcd for C_60_H_71_N_8_O_14_S 1159.4805, found 1159.4802; [α]^24^_D_ − 189.17 (_*C*_ 0.23, DMSO).

### {N-(Anthraquinone-2carbonyl)-D-Ser-L-Ala-N-Boc-N-Me-L-Cys-N-Me-L-Val}_2_ sulfide (**19**)

Compound **19** (16.0 mg, 13.2 µmol, 62% over 2 steps) was synthesized using methods previously reported by our group^20^, from anthoraquinone-2-carboxylic acid (16.0 mg, 63.6 µmol), and purified with high flash silica gel column chromatography ($\Phi$ 2 × 6 cm, CHCl_3_/MeOH: 100/0 → 99/1 → 98/2) as a pale yellow solid. ^1^H NMR (DMSO-*d*_6_, 400 MHz) δ 8.87 (d, 2H, Ser-N*H*, *J*_Ser-NH, Ser-α-CH_ = 7.8 Hz), 8.60 (s, 2H, Ar), 8.32 (s, 4H, Ar), 8.23 (dd, 4H, Ar, *J* = 4.6, 3.2 Hz), 7.96–7.94 (m, 4H, Ar), 7.91 (d, 2H, Ala-N*H*,* J*_Ala-NH, Ala-α-CH_ = 5.5 Hz), 6.30 (t, 2H, Cys-α-C*H*, *J*_Cys-α-CH, Cys-β-CH_ = 6.9 Hz), 4.85 (d, 2H, Val-α-C*H*, *J*_Val-α-CH, Val-β-CH_ = 10.5 Hz), 4.68 (m, 2H, Ser-α-C*H*), 4.59 (m, 2H, Ala-α-C*H*), 4.49 (m, 2H, Ser-β-C*H*), 4.35 (d, 2H, Ser-β-C*H*, *J*_gem_ = 9.6 Hz), 3.10 (dd, 2H, Cys-β-C*H*, *J*_gem_ = 14.2, *J*_Cys-β-CH, Cys-α-CH_ = 6.9 Hz), 3.05 (s, 6H, *N*–C*H*), 2.81 (s, 6H, *N*–C*H*), 2.54 (overlap, 2H, Cys-β-C*H*), 2.26 (m, 2H, Val-β-C*H*), 1.29 (d, 6H, Ala-β-C*H*, *J*_Ala-β-CH, Ala-α-CH_ = 6.9 Hz), 0.99 (d, 6H, Val-γ-C*H*, *J*_Val-γ-CH, Val-β-CH_ = 6.4 Hz), 0.77 (d, 6H, Val-γ-C*H*, *J*_Val-γ-CH, Val-β-CH_ = 6.9 Hz); ^13^C NMR (DMSO-*d*_6_, 100 MHz) δ 182.1, 172.3, 170.3, 169.8, 167.6, 165.2, 138.7, 134.9, 134.8, 133.3, 133.1, 133.0, 127.2, 126.9, 125.8, 64.6, 61.5, 54.2, 53.3, 46.1, 35.3, 30.5, 29.8, 26.5, 20.3, 18.8, 16.6; ESIMS-LR *m/z* 1233.60 [(M + Na)^+^]; ESIMS-HR calcd for C_62_H_67_N_8_O_16_S 1211.4390, found 1211.4396; [α]^24^_D_ − 192.98 (_*C*_ 0.79, DMSO).

### {N-(Pyradine-2carbonyl)-D-Ser-L-Ala-N-Boc-N-Me-L-Cys-N-Me-L-Val}_2_ sulfide (**20**)

Compound **20** (8.3 mg, 8.7 µmol, 49% over 2 steps) was synthesized using methods previously reported by our group^20^, from pyrazine-2-carboxylic acid (7.4 mg, 63.6 µmol), and purified with high flash silica gel column chromatography ($\Phi$ 1 × 10 cm, CHCl_3_/MeOH: 100/0 → 98/2 → 96/4 → 94/6) as a white solid. ^1^H NMR (CDCl_3_, 400 MHz) δ 9.41 (d, 2H, Ar,* J* = 1.4 Hz), 8.80 (d, 2H, Ar,* J* = 2.3 Hz), 8.50 (dd, 2H, Ar,* J* = 2.3, 1.4 Hz), 8.45 (d, 2H, Ser-N*H*, *J*_Ser-NH, Ser-α-CH_ = 7.3 Hz), 6.72 (d, 2H, Ala-N*H*, *J*_Ala-NH, Ala-α-CH_ = 7.3 Hz), 6.23 (dd, 2H, Cys-α-C*H*, *J*_Cys-α-CH, Cys-β-CH_ = 6.9, 6.4 Hz), 5.10 (d, 2H, Val-α-C*H*, *J*_Val-α-CH, Val-β-CH_ = 10.1 Hz), 4.88–4.85 (m, 2H, Ser-α-C*H*), 4.80 (dq, 2H, Ala-α-C*H*, *J*_Ala-NH, Ala-α-CH_ = 7.3, *J*_Ala-α-CH, Ala-β-CH_ = 7.3 Hz), 4.75 (dd, 2H, Ser-β-C*H*, *J*_gem_ = 11.4, *J*_Ser-β-CH, Ser-α-CH_ = 4.6 Hz), 4.63 (dd, 2H, Ser-β-C*H*, *J*_gem_ = 11.4, *J*_Ser-β-CH, Ser-α-CH_ = 1.4 Hz), 3.36 (dd, 2H, Cys-β-C*H*, *J*_gem_ = 15.1, *J*_Cys-β-CH, Cys-α-CH_ = 6.4 Hz), 3.11 (s, 6H, *N*–C*H*), 2.93 (s, 6H, *N*–C*H*), 2.47 (dd, 2H, Cys-β-C*H*, *J*_gem_ = 15.1, *J*_Cys-β-CH, Cys-α-CH_ = 6.9 Hz), 2.24 (m, 2H, Val-β-C*H*), 1.35 (d, 6H, Ala-β-C*H*, *J*_Ala-β-CH, Ala-α-CH_ = 7.3 Hz), 1.06 (d, 6H, Val-γ-C*H*, *J*_Val-γ-CH, Val-β-CH_ = 6.9 Hz), 0.84 (d, 6H, Val-γ-C*H*, *J*_Val-γ-CH, Val-β-CH_ = 6.9 Hz); ^13^C NMR (CDCl_3_, 100 MHz) δ 173.2, 171.4, 170.0, 167.5, 164.1, 148.2, 144.8, 143.4, 142.9, 64.0, 32.0, 54.9, 53.3, 46.4, 36.1, 31.4, 30.3, 27.7, 20.5, 19.0, 17.5; ESIMS-LR m/z 955.48 [(M + H)^+^]; ESIMS-HR not obtained; [α]^19^_D_ − 201.55 (_*C*_ 0.83, CHCl_3_).

#### Echinomycin probe **27**

Compound **27** was prepared using methods previously reported by our group^20^. Namely, **25** (5.9 mg, 4.4 µmol) was treated with triisopropylsilane (0.96 µL, 4.96 µmol) and 80% *aq*. TFA (400 µL) at room temperature for 2.5 h. The reaction mixture was concentrated *in vacuo*, and then azeotroped with toluene to afford a crude carboxylic acid. A mixture of the residue and biotin-PEG2-amine** 26** (2.2 mg, 5.9 µmol) in 50% CHCl_3_/MeOH (100 µL) was treated with DMT-MM (1.8 mg, 6.5 µmol) at room temperature for 3.5 h. The reaction mixture was concentrated *in vacuo*, and the crude was purified by preparative TLC (20 × 20 cm, 10% MeOH in CHCl_3_) to afford an inseparable mixture containing unreacted carboxylic acid (3.6 mg). Then the crude carboxylic acid (3.6 mg), ^*i*^Pr_2_NEt (1.90 µL, 11.2 µmol), EDCI (0.8 mg, 4.2 µmol) and HOAt (0.6 mg, 4.2 µmol) in DMF (100 µL) was treated with biotin-PEG2-amine (**26**) (2.1 mg, 5.6 µmol) at room temperature for 14 h. The reaction mixture was concentrated *in vacuo*, and then azeotroped with toluene to afford a white solid. The crude was purified by preparative TLC (20 × 20 cm, 10% MeOH in CHCl_3_) to afford compound **27** (1.94 mg, 1.18 µmol, 27% over 3 steps) as a white amorphous solid. ^1^H NMR (CDCl_3_, 400 MHz) δ 9.64 (s, 1H, Ar–*H*), 9.64 (s, 1H, Ar–*H*), 8.83 (d, 1H, Ser-N*H*, *J*_Ser-N*H*, Ser-α-C*H*_ = 6.4 Hz), 8.69 (d, 1H, Ser-N*H*, *J*_Ser-N*H*, Ser-α-C*H*_ = 7.6 Hz), 8.20 (d, 1H, Ar–*H*, *J* = 4.8 Hz), 8.18 (d, 1H, Ar–*H*, *J* = 5.2 Hz), 8.00 (d, 1H, Ar–*H*, *J* = 7.6 Hz), 7.96 (d, 1H, Ar–*H*, *J* = 8.0 Hz), 7.90–7.80 (m, 4H, Ar–*H*), 7.01–6.97 (m, 3H, Ala-N*H*, biotin-N*H*), 6.56 (s, 1H, biotin-N*H*), 6.43 (d, 1H, Cys-α-C*H*, *J*_Cys-α-C*H*, Cys-β-C*H*_ = 8.4 Hz), 6.19 (d, 1H, Cys-α-C*H*, *J*_Cys-α-C*H*, Cys-β-C*H*_ = 10.4 Hz), 5.59 (s, 1H, CON*H*), 5.59 (s, 1H, CON*H*), 5.16–5.11 (m, 3H, Val-α-C*H*, Cys-β-C*H*), 4.98–4.91 (m, 2H, Ser-α-C*H*, Ala-α-C*H*), 4.85–4.79 (m, 2H, Ser-α-C*H*, Ala-α-C*H*), 4.72–4.59 (m, 4H, Ser-β-C*H*), 4.51–4.48 (m, 1H, biotin-NHC*H*), 4.31–4.29 (m, 1H, biotin-NHC*H*), 3.78–3.40 (m, 25H, Cys-β-C*H*, H_*b*_, H_*c*_, H_*d*_, H_*e*_, H_*f*_, H_*g*_, H_*i*_, H_*j*_, H_*k*_, H_*l*_, H_*m*_, H_*n*_), 3.16–3.11 (m, 7H, NC*H*, biotin-SC*H*), 3.00 (s, 3H, NC*H*), 2.99 (s, 3H, NC*H*), 2.94–2.80 (m, 3H, Cys-β-C*H*, H_*h*_), 2.74–2.68 (m, 2H, biotin-SC*H*_2_), 2.51 (t, 2H, H_*a*_, *J*_H*a*, Hb_ = 6.0 Hz), 2.38–2.28 (m, 2H, Val-β-C*H*), 2.23 (t, 2H, H_*o*_, *J*_H*o*, H*p*_ = 7.2 Hz), 1.79–1.63 (m, 2H, H_*p*_), 1.49–1.43 (m, 2H, H_*r*_), 1.40 (d, 3H, Ala-β-C*H*, *J*_Ala-β-C*H*, Ala-α-C*H*_ = 6.4 Hz), 1.39 (d, 3H, Ala-β-C*H*, *J*_Ala-β-C*H*, Ala-α-C*H*_ = 6.0 Hz), 1.32–1.25 (m, 2H, H_*q*_), 1.09 (d, 3H, Val-γ-C*H*, *J*_Val-γ-C*H*, Val-β-C*H*_ = 6.4 Hz), 1.07 (d, 3H, Val-γ-C*H*, *J*_Val-γ-C*H*, Val-β-C*H*_ = 6.0 Hz), 0.92 (d, 3H, Val-γ-C*H*, *J*_Val-γ-C*H*, Val-β-C*H*_ = 6.8 Hz), 0.87 (d, 3H, Val-γ-C*H*, *J*_Val-γ-C*H*, Val-β-C*H*_ = 6.4 Hz); ^13^C NMR (CDCl_3_, 100 MHz) δ173.8, 173.5, 173.3, 172.0, 171.2, 170.9, 170.4, 169.0, 167.8, 167.5, 164.3, 164.1, 163.2, 144.4, 144.3, 143.8, 143.8, 142.5, 142.5, 140.3, 140.3, 132.3, 132.2, 131.3, 131.2, 129.9, 129.8, 129.6, 129.5, 77.4, 70.6, 70.5, 70.4, 70.3, 70.0, 67.5, 65.2, 65.1, 62.9, 62.1, 61.9, 60.2, 60.0, 55.4, 53.7, 53.5, 52.4, 46.8, 46.4, 40.7, 39.3, 37.1, 35.9, 32.5, 31.5, 31.0, 30.4, 30.0, 29.8, 28.2, 27.9, 27.8, 25.5, 20.6, 20.5, 19.3, 18.9, 18.3, 17.4; ESIMS-LR *m*/*z* 1669.6 [(M + Na)^+^]; ESIMS-HR calcd for C_75_H_106_N_16_NaO_20_S_3_ 1669.6824, found 1669.6798; [α]^23^_D_ − 184.42 (*c* 0.19, CHCl_3_).

#### Echinomycin probe **29**

Compound **29** was prepared using methods previously reported by our group^20^. Namely, **23** (27.8 mg, 26.0 µmol) in CH_2_Cl_2_ (519 µL) was treated with acetyl chloride (5.6 µL, 77.9 µmol) at room temperature for 1.5 h. Acetyl chloride (5.6 µL, 77.9 µmol) was added to the mixture, and the whole mixture was further stirred for 1 h. The reaction mixture was concentrated *in vauo*, and then azeotroped with toluene to afford a pale yellow solid. A mixture of the residue, 2-(3-but-3-ynyl-3*H*-diazirin-3-yl)-ethanol (**28**) (35.9 mg, 259.0 µmol) in THF (200 µL) was treated with zinc bromide (29.2 mg, 130 µmol) at room temperature for 20 h. The reaction mixture was diluted with EtOAc (50 mL) and CHCl_3_ (10 mL), and washed with H_2_O (20 mL × 2) and brine (20 mL). The organic layer was dried over Na_2_SO_4_, filtered, concentrated *in vacuo* and washed with Et_2_O. The crude was purified by preparative TLC (20 × 20 cm, 5% MeOH in CHCl_3_) to afford compound **29** (0.9 mg, 0.76 µmol, 3%) as a white amorphous solid. ^1^H NMR (CDCl_3_, 400 MHz) δ 9.64 (s, 1H, Ar–*H*), 9.64 (s, 1H, Ar–*H*), 8.82 (d, 1H, Ser-N*H*, *J*_Ser-N*H*, Ser-α-C*H*_ = 6.0 Hz), 8.74 (d, 1H, Ser-N*H*, *J*_Ser-N*H*, Ser-α-C*H*_ = 6.9 Hz), 8.20 (dd, 1H, Ar–*H*, *J* = 3.2, 1.6 Hz), 8.18 (dd, 1H, Ar–*H*, *J* = 3.4, 1.1 Hz), 8.04 (d, 1H, Ar–*H*, *J* = 6.9 Hz), 8.02 (d, 1H, Ar–*H*, *J* = 7.8 Hz), 7.90–7.81 (m, 4H, Ar–*H*), 7.07–7.04 (m, 2H, Ala-N*H*), 6.46 (d, 2H, Cys-α-C*H*, *J*_Cys-α-C*H*, Cys-β-C*H*_ = 7.3 Hz), 5.18–5.13 (m, 3H, Val-α-C*H*, Cys-β-C*H*), 4.96–4.82 (m, 4H, Ser-α-C*H*, Ala-α-C*H*), 4.71–4.56 (m, 4H, Ser-β-C*H*), 3.76 (td, 1H, H-1, *J*_gem_ = 9.7, *J*_H-1, H-2_ = 6.6 Hz), 3.24–3.19 (m, 1H, H-1), 3.12 (s, 3H, NC*H*), 3.08 (s, 3H, NC*H*), 3.04–3.02 (m, 1H, Cys-β-C*H*), 2.97 (s, 3H, NC*H*), 2.96 (s, 3H, NC*H*), 2.78–2.71 (m, 1H, Cys-β-C*H*), 2.40–2.27 (m, 2H, Val-β-C*H*), 2.03–2.00 (m, 2H, H-3, H-5), 1.93–1.86 (1H, m, H-2), 1.78–1.71 (m, 1H, H-2), 1.66–1.62 (m, 1H, H-3), 1.44–1.41 (m, 1H, H-4), 1.43 (d, 3H, Ala-β-C*H*, *J*_Ala-β-C*H*, Ala-α-C*H*_ = 6.9 Hz), 1.42 (d, 3H, Ala-β-C*H*, *J*_Ala-β-C*H*, Ala-α-C*H*_ = 6.9 Hz), 1.36–1.25 (m, 1H, H-4), 1.08 (d, 6H, Val-γ-C*H*, *J*_Val-γ-C*H*, Val-β-C*H*_ = 6.0 Hz), 0.90 (d, 3H, Val-γ-C*H*, *J*_Val-γ-C*H*, Val-β-C*H*_ = 6.9 Hz), 0.86 (d, 3H, Val-γ-C*H*, *J*_Val-γ-C*H*, Val-β-C*H*_ = 6.9 Hz); ^13^C NMR (CDCl_3_, 100 MHz) δ174.3, 173.6, 171.2, 170.2, 168.8, 164.3, 164.1, 144.3, 143.8, 143.8, 142.6, 140.4, 140.3, 132.2, 131.2, 131.2, 129.8, 129.7, 129.7, 83.0, 77.4, 69.7, 65.3, 65.1, 64.8, 62.4, 62.0, 59.5, 53.6, 53.3, 52.5, 46.9, 46.7, 32.8, 32.1, 32.0, 31.2, 30.8, 29.9, 29.8, 27.6, 27.5, 26.9, 20.5, 20.5, 18.9, 18.6, 18.2, 18.0, 13.3; ESIMS-LR *m*/*z* 1213.6 [(M + Na)^+^]; ESIMS-HR calcd. for C_57_H_70_N_14_NaO_13_S 1213.4860, found 1213.4882; [α]^24^_D_ − 239.91 (*c* 0.42, CHCl_3_).

### Evaluation of cytotoxicity

Basically, the evaluation was performed using methods previously reported by our group^20,23^. Cytotoxic activities of compounds against MIA PaCa-2 (American Type Culture Collection), SW620 (European Collection of Authenticated Cell Cultures) were measured by WST-8 assay according to manufacturer’s protocol. Briefly, cells (1 × 10^4^ cells/well) in a 96-well plate were cultured in medium (for MIA PaCa-2, D-MEM (Wako, Japan) containing 10% fetal bovine serum (Funakoshi, Japan), 100 units/mL penicillin G and 100 µg/mL streptomycin (Wako, Japan), for SW620, RPMI-1640 (Wako, Japan) containing 10% fetal bovine serum, 100 units/mL penicillin G and 100 µg/mL streptomycin) were cultured at 37 °C for 24 h under 5% CO_2_ atmosphere. Then, cells were treated with test compounds (final DMSO concentration was 1%) at 37 °C for 72 h under 5% CO_2_ atmosphere. Then, cells were treated with a solution of Cell Counting Kit-8® (Dojindo, Japan) for 3 h. After that, cell viabilities were determined based on the measurement of absorption at 450 nm of the wells by using TECAN Infinite® M200 microplate reader. The results were expressed as a percentage of the negative control (cells treated with DMSO only), and that value was fixed at 100%. IC_50_ values were calculated by using the Microsoft Excel 15.24 software and the GraphPad Prism 8.3.0/9.0 software and each data is shown as mean ± SE (n = 3) in Table 3.

#### Western blotting

Basically, the analysis was performed using methods previously reported by our group^23^. Proteins were extracted from samples using NP-40 Cell Lysis Buffer (Thermo Fisher Scientific, MA, USA) supplemented with Complete protease inhibitor (Sigma Aldrich) and Halt Phosphatase Inhibitor Cocktail (Thermo Fisher Scientific). Equal amounts of protein were separated by SDS-PAGE (12.5%), transferred onto a nitrocellulose membrane, and blocked with SuperBlock T-20 (PBS; ThermoFisher Scientific). Primary antibody of cleaved caspase-3 (Cell Signaling Technology, Inc, MA, USA, #9661) were diluted to 1:1000 in SuperBlock T-20 (PBS) and incubated with blots overnight at 4 ℃. After washing in T-PBS, blots were probed with HRP-conjugated secondary antibodies (R&D Systems, Inc., MN, USA), washed in T-PBS, and then developed using the Super-Signal West Pico enhanced chemiluminescence system (ThermoFisher Scientific). Protein expression levels were normalized to the actin expression (BD Transduction Laboratories, KY, USA).

### Terminal deoxynucleotidyl transferase dUTP nick-end labeling (TUNEL) staining

Basically, the analysis was performed using methods previously reported by our group^23^. SW-620 cells were plated on a Lab-Tek^®^ Chamber Slide™ (ThermoFisher Scientific) 24 h prior to stimulation. After fixing the slides in 4% paraformaldehyde and through PBS washing, an In Situ Cell Death Detection Kit and TMR red (Roche Diagnostic, Indianapolis, IN, USA) were used for staining according to the manufacturer’s instructions. The cells were mounted with an anti-fade mounting medium, and fluorescence microscopy (KEYENCE Corporation, Osaka, Japan) was employed for visualizing TUNEL-positive cells.

### Reporter gene assay

Basically, the assay was performed using methods previously reported by our group^21^. SW620 cells were transfected with either HIF-1α expression vector pCI-neo-3 × FLAG-HIF-1α or empty vector pCI-neo-3 × FLAG, a derivative of pCI-neo (Promega, Madison, WI, USA), together with pGL3-5xHRE-Luc and pGL4.75 [hRluc/CMV] (Promega), by using PEI MAX (Polysciences Inc., Warrington, PA, USA). At one day after transfection, the cells were exposed to the test compounds for 16 h, and then cell lysates were prepared. A dual-luciferase assay was performed with a dual-luciferase reporter assay system (Promega) and GloMax20/20 luminometer (Promega).

### Transcriptome analysis

Basically, the analysis was performed using methods previously reported by our group^23^. RNA libraries were generated using an Ion Total RNA-Seq Kit v2 (Thermo Fisher Scientific) according to the manufacturer’s instructions. Emulsion PCR was carried out with an Ion OneTouchTM system and an Ion OneTouch 200 Template Kit v3 (Thermo Fisher Scientific). Template-positive Ion Sphere™ particles were enriched and purified for the sequencing reaction with an Ion OneTouch™ ES system (Thermo Fisher Scientific). The template-positive Ion Sphere™ Particles were loaded onto Ion PI™ Chips (Thermo Fisher Scientific) and for high throughput sequencing with an Ion Proton™ Semiconductor sequencer (Thermo Fisher Scientific). Sequencing data were mapped on a human reference genome sequence (GRCh38/hg38) using the Torrent Suite software program (Life Technologies). The expression analysis was performed in the CLC Genomics Workbench software program (CLC bio, Aarhus, Denmark), and differences among the samples were determined using an unpaired Student’s t-test. The gene list describing fold change and p-value was uploaded to the MetaCore software (Clarivate Analytics, PA, USA, URL; https://portal.genego.com/, version 6.33.69110.), and then pathway analysis was performed.

### Hepatic microsomal stability assay

Basically, the analysis was performed using methods previously reported^31^. Disappearance of the parent compound over time was measured by using the amount of drug at time zero as a reference. After 5 min of preincubation, 1 mM NADPH (final concentration, the same applies to the following, NADPH( +)) or 0.1 M phosphate buffer (pH 7.4, NADPH(−))) was added to a mixture containing 1 μM of the test compound, 0.2 mg/mL of human or mouse liver microsomes (Sekisui XenoTech LLC (Kansas City, KS)), 1 mM EDTA and 0.1 M phosphate buffer (pH 7.4) and incubated at 37 °C for 30 min by rotation at 60 rpm. An aliquot of 50 μL of the incubation mixture was sampled and added to 250 μL of chilled acetonitrile/internal standard (IS). After centrifuging for 15 min at 3150×*g* (4 °C), the supernatants were analyzed by LC–MS/MS. Hepatic microsomal stability (mL/min/kg, CLint) was calculated according to the previous report^32^, using 48.8 and 45.4 mg MS protein/g liver and 25.7 and 87.5 g liver/kg body weight as scaling factors for human and mouse, respectively.

### LC–MS/MS quantification method

Basically, the analysis was performed using methods previously reported^31^. An LC-MS8060 instrument equipped with a Shimadzu Nexera series LC system (Shimadzu, Kyoto, Japan) was used. All compounds were analyzed in multi-reaction monitoring mode under electron spray ionization conditions. The analytical column used was a CAPCELLPAK C18 MGIII (3 μm × 2.0 mmID × 35 mm; OSAKA SODA, Osaka, Japan) at 50 °C. The gradient mobile phase consisted of 0.1% formic acid in water (mobile phase A,) and 0.1% formic acid in acetonitrile (mobile phase B) at a total flow rate of 1 mL/min. The initial mobile phase composition was 10% B, which was held constant for 0.2 min, increased in a linear fashion to 90% B over 1 min, then held constant for 0.8 min, and finally brought back to the initial condition of 10% B over 0.01 min and re-equilibrated for 1 min. The transitions (precursor ion > product ion) of echinomycin, **3** and IS (methyl testosterone) are 1101.45 > 1053.5 (positive), 1083.5 > 523.95 (negative), and 303.1 > 109.1 (positive), respectively.

#### Xenografts

Basically, the experiments were performed using methods previously reported^33^. Male BALB/c nude mice (6 weeks old) were purchased from Charles River Laboratories Japan, Inc. SW-620 cells (2 × 10^6^ cells/35 μL PBS/tumor) were mixed with matrigel (15 μL/tumor) (BD Transduction Laboratories). Subsequently, 50 μL of cell suspension was subcutaneously injected into the back of BALB/c nude mice. Daily intraperitoneal injections of each compound began on the day following the SW-620 cell injection. The tumor size was calculated by the following formula: Tumor size (mm^2^) = (major diameter) × (minor diameter).

### Serum biochemistry

Male BALB/c mice (7 weeks old) were purchased from Charles River Laboratories Japan, Inc. Each compound was intraperitoneally injected daily for 14 days. The whole blood of mice was obtained from the inferior vena cava under anesthesia with isoflurane by inhalation. Serum was collected by centrifuging blood at 5000 rpm for 10 min. Biochemistry was performed in Oriental Yeast Co., Ltd, Japan.

### Ethics declaration

The study received ethical approval for the use of an opt-out methodology from the Medical Ethics Committee of Asahikawa Medical University (Approval No. 16069, 20057). All methods were carried out in accordance with relevant guidelines and regulations, and they are reported in compliance with the ARRIVE guidelines.

### Supplementary Information

Supplementary Information 1.

Supplementary Information 2.

Supplementary Information 3.

## Data Availability

Data supporting the findings of this manuscript are available from the corresponding author upon request.
