# Supplementary material for: Synthesis and biological evaluation of echinomycin analogues as potential colon cancer agent
Source: Sci Rep. 2024 Apr 1;14:7628. doi: 10.1038/s41598-024-58196-3 (PMC10985088; doi:10.1038/s41598-024-58196-3)
Supplement: Supplementary file 1 — Supplementary Information 1. [file 41598_2024_58196_MOESM1_ESM.docx]

Supporting Information

Synthesis and Biological Evaluation of Echinomycin Analogues as Potential Colon Cancer Agent

Keita Kojima, Hiroaki Konishi, Kyoka Momosaki, Yuya Komatani, Akira Katsuyama, Koji Nakagawa, Kayoko Kanamitsu, Fumika Yakushiji, Mikihiro Fujiya, and Satoshi Ichikawa^*^

Table of Contents

1. **HPLC charts of compounds S2-S9**
2. **NMR spectrum of synthesized compounds S10-S32**
3. **HRMS spectrum of compounds S31-S52**
4. **Investigation of maximum tolerated doses S53**
5. **Blood hematological examination of mice treated with 1 and 3 S54**
6. **Full-length gels and blots for Figure 4 S55**
7. **Concentration-response curves of *in vitro* cytotoxic activity assay S56-S57**
8. **HPLC charts of compounds** (asterisks indicate impurities contained in solvents)

LC equipment: Shimadzu, Prominence-i LC-2030C Plus, LCMS-8040; column: J’sphere ODS-M80, 150 × 4.6 nml.D., S-4 μm, 8 nm; column oven: 30 °C; eluent: isocratic elution of 20% B over 1 min; linear gradient elution of 20% B-100% B over 15 min; isocratic elution of 100% B over 6 min; linear gradient elution of 100% B-20% B over 1 min; isocratic elution of 20% B over 8 min (where solvent A was 0.1% HCO_2_H in H_2_O and solvent B was MeCN); flow rate: 0.40 mL/min; detection: UV (254 nm).

DMSO (background)

*

*

*

*

Echinomycin (**1**)

*

*

*

*

**1**

Compound **2**

*

*

*

*

**2**

Compound **3**

*

*

*

**3**

Compound **4**

*

**4**

Compound **5**

*

*

**5**

Compound **6**

*

*

**6**

Compound **7**

*

*

**7**

Compound **8**

*

*

*

*

**8**

Compound **9**

*

*

*

*

**9**

Compound **10**

*

*

*

*

**10**

Compound **11**

*

*

*

*

**11**

Compound **12**

*

*

*

*

**12**

Compound **13**

*

*

*

*

**13**

Compound **14**

*

*

*

*

**14**

Compound **15**

*

**15**

Compound **16**

*

*

*

*

**16**

Compound **17**

*

*

*

**17**

Compound **18**

*

*

*

*

**18**

Compound **19**

*

**19**

Compound **20**

*

*

*

*

**20**

1. **NMR spectrum of synthesized compounds**

Compound **22**

Compound **4**


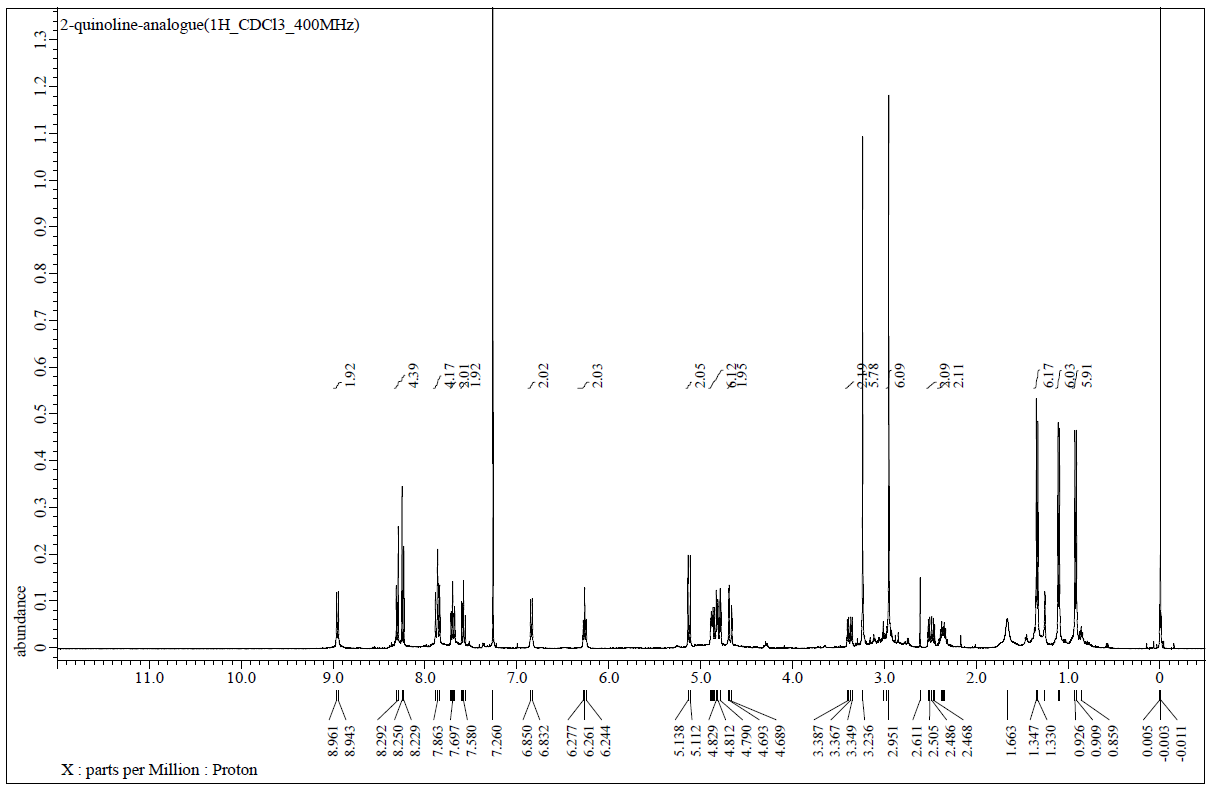


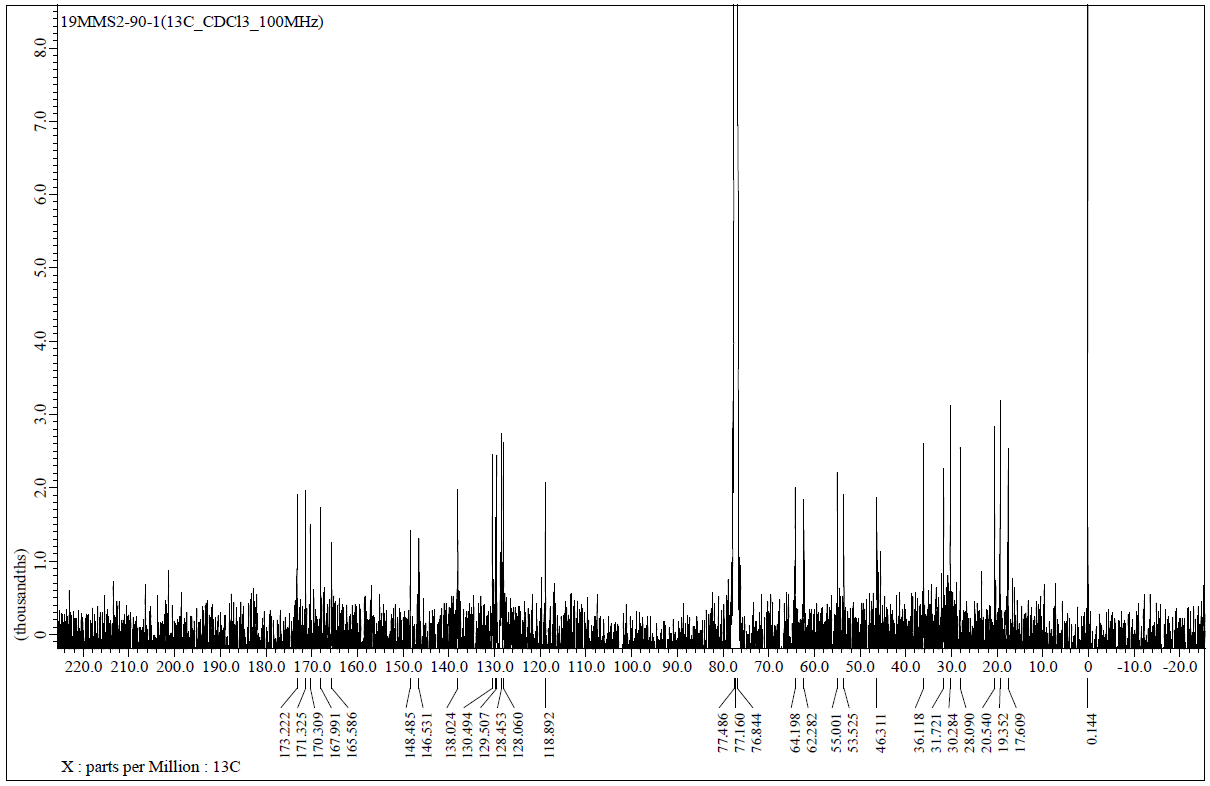


Compound **5**


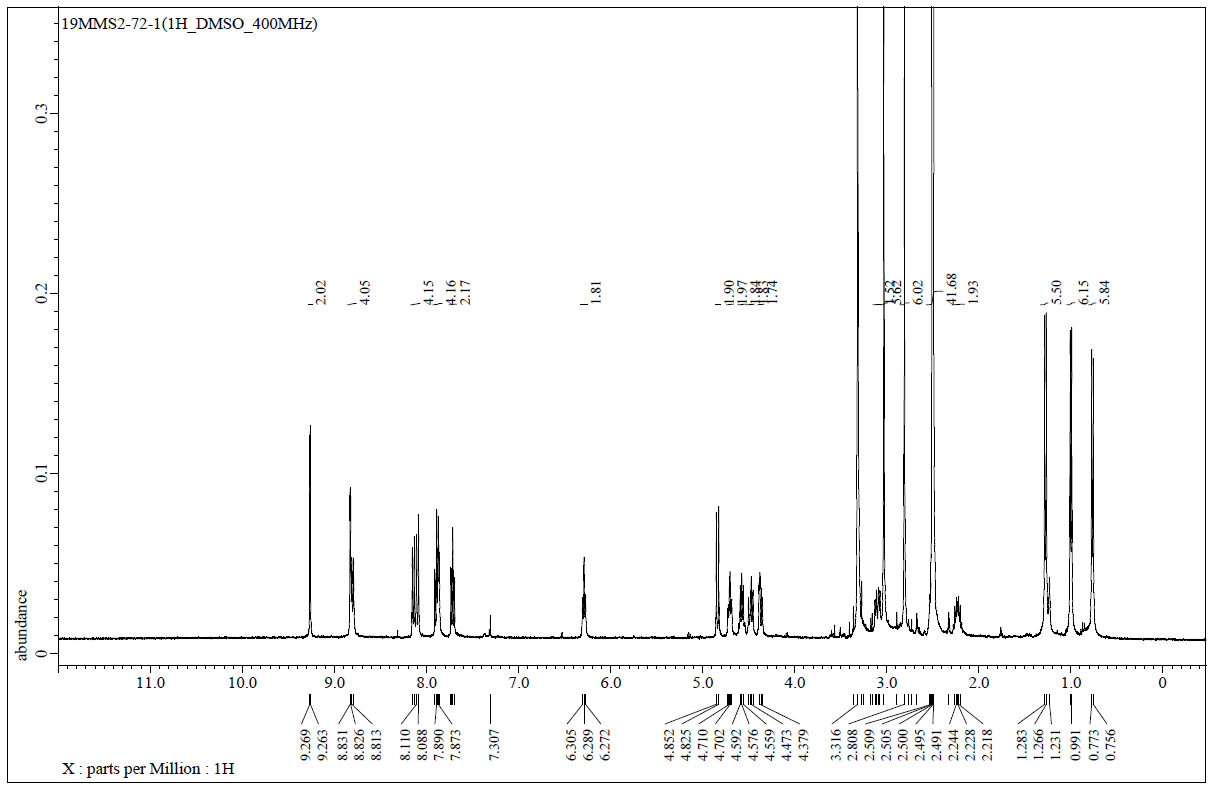


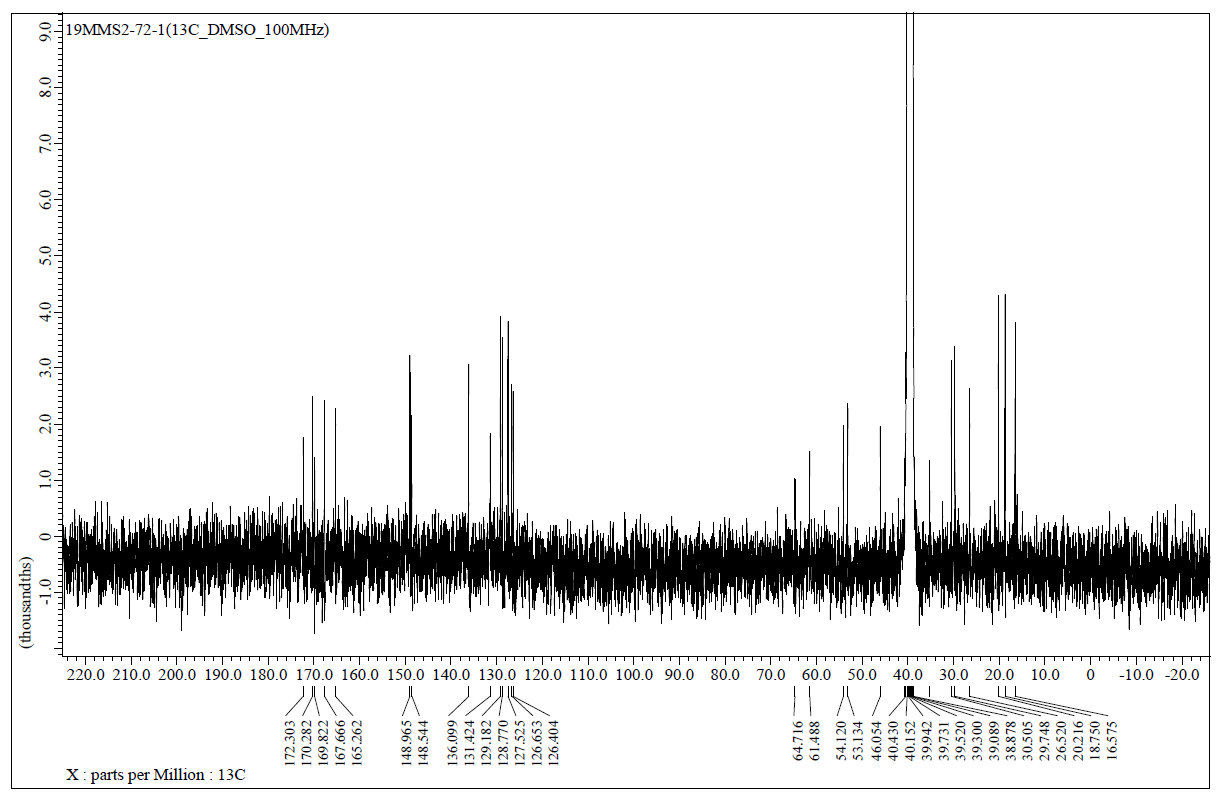


Compound **6**


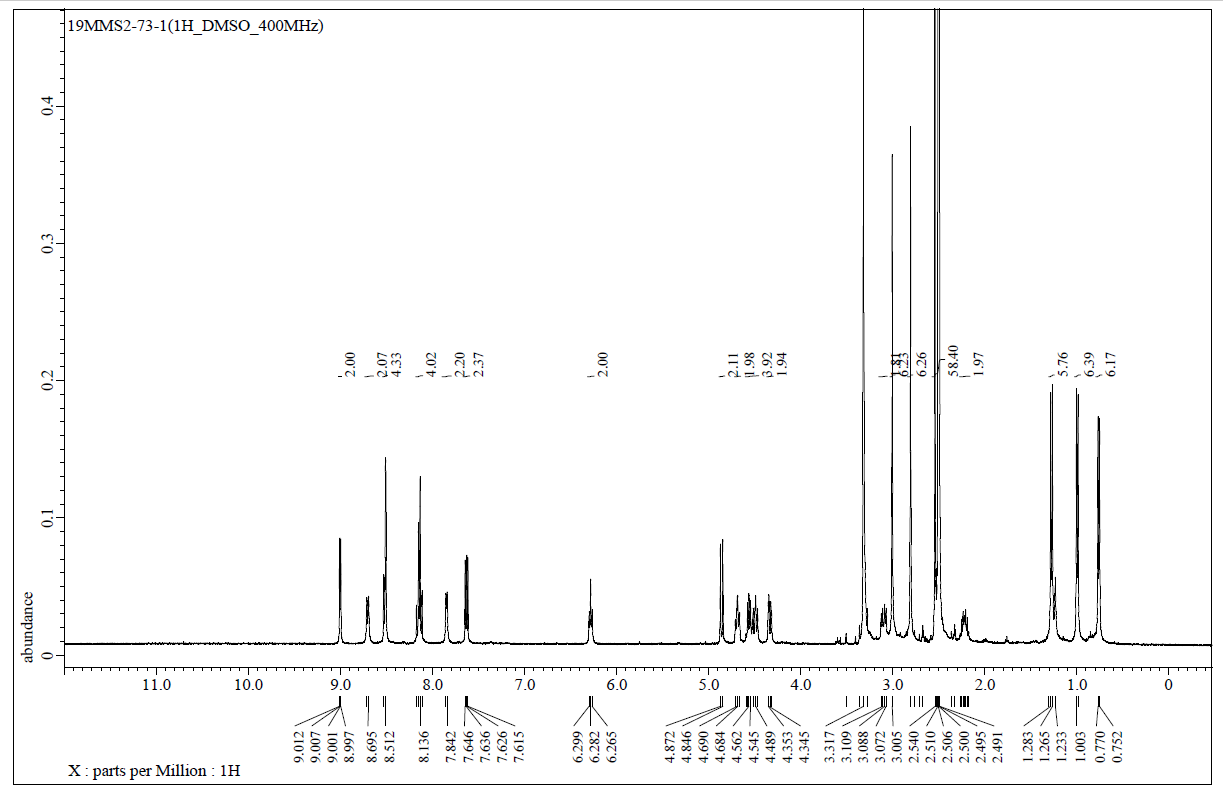


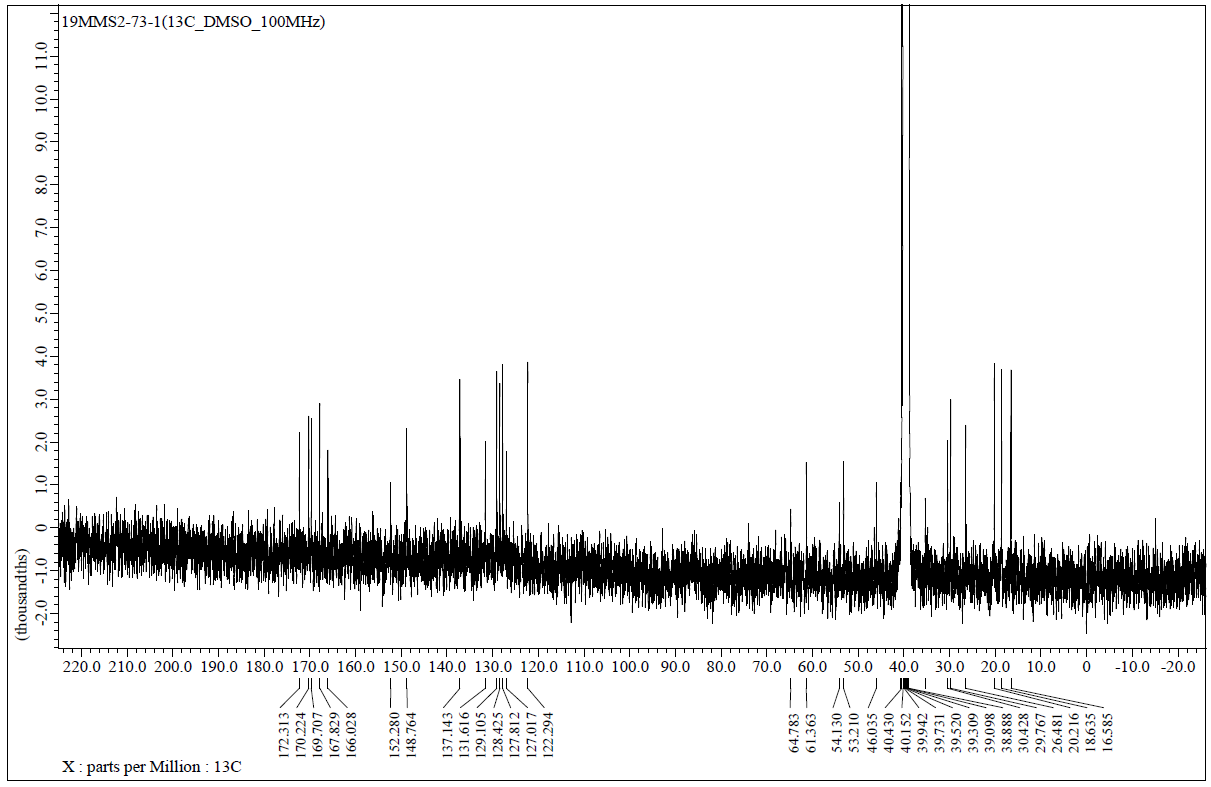


Compound **7**


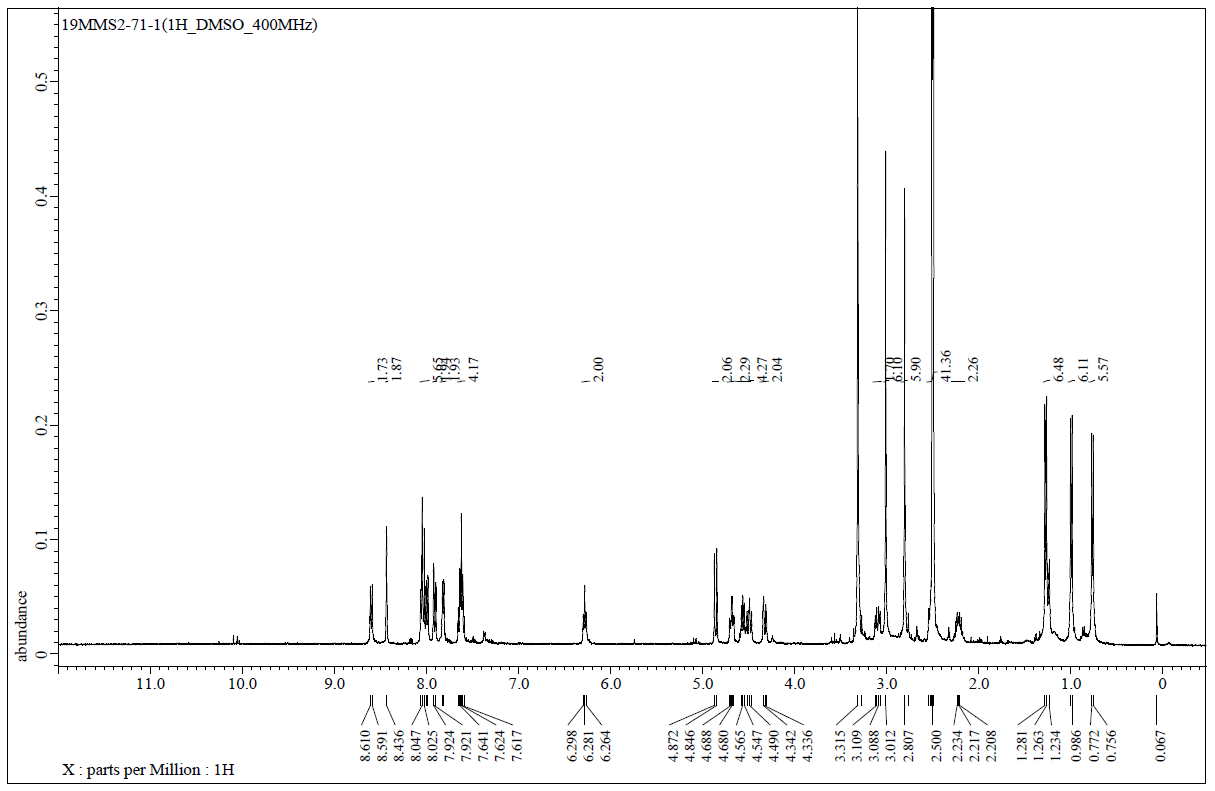


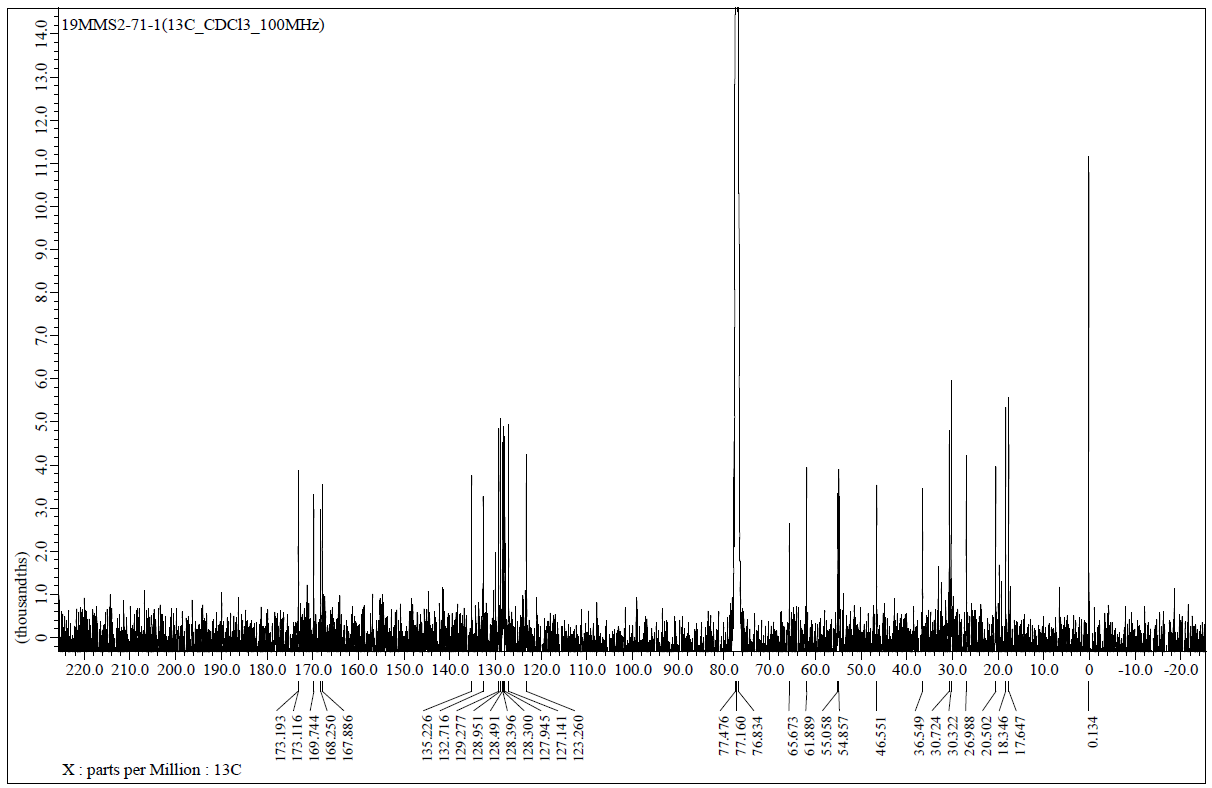


Compound **8**

Compound **9**


Compound **10**

Compound **11**

Compound **12**

Compound **13**

Compound **14**

Compound **15**

Compound **16**

Compound **17**

Compound **18**

Compound **19**

Compound **20**

Compound **25**


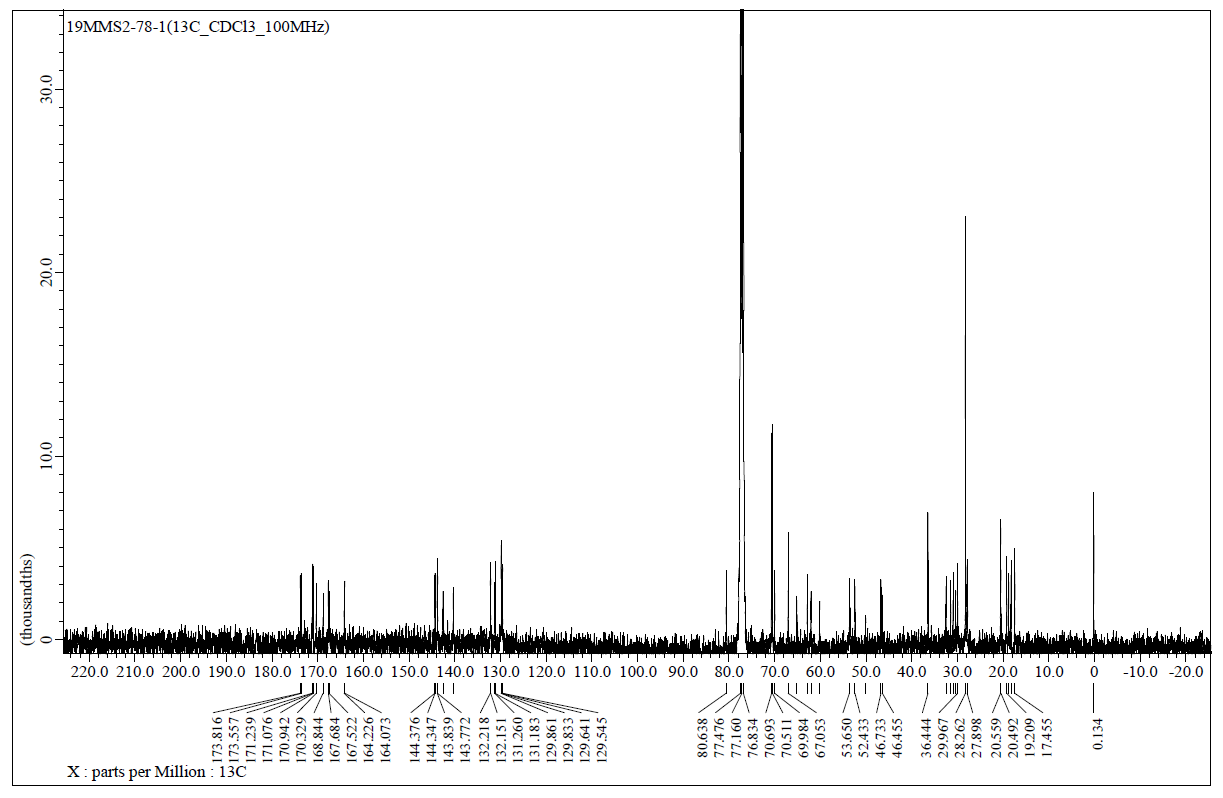

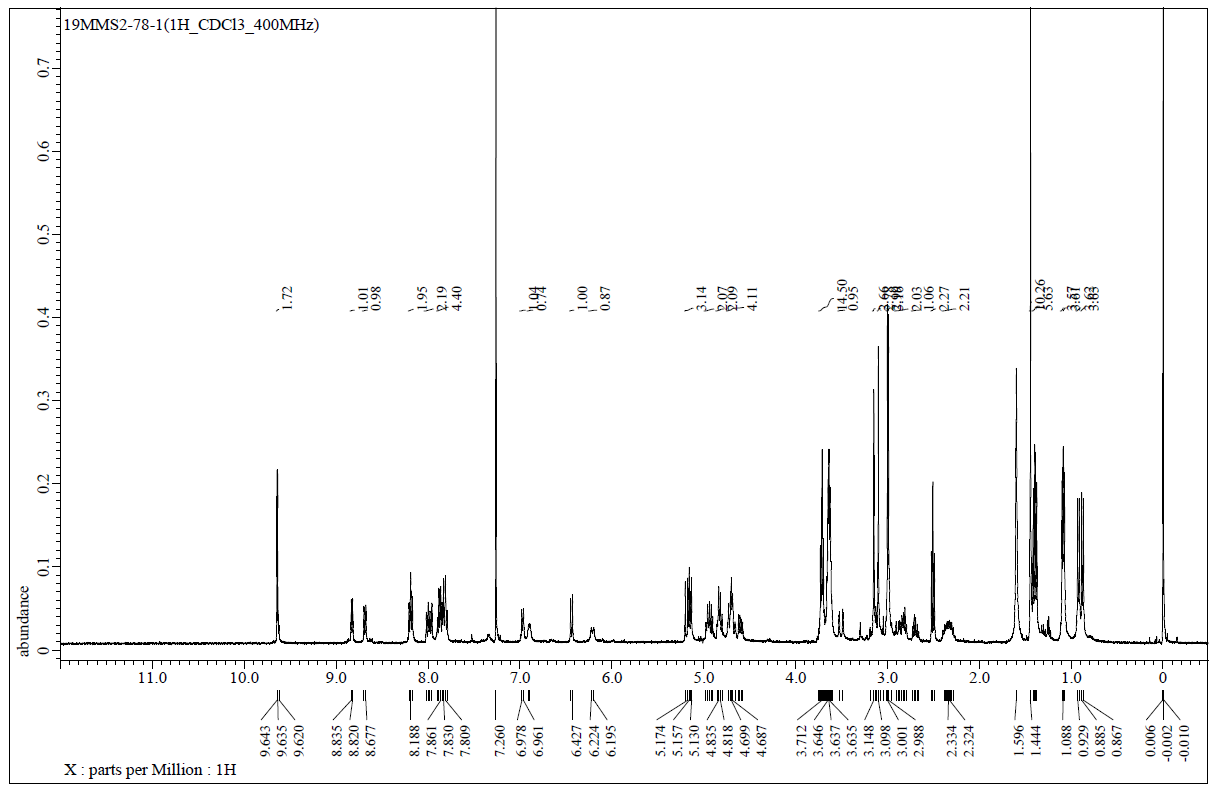


**27**

(CDCl_3_, 400 MHz)

**27**

(CDCl_3_, 100 MHz)


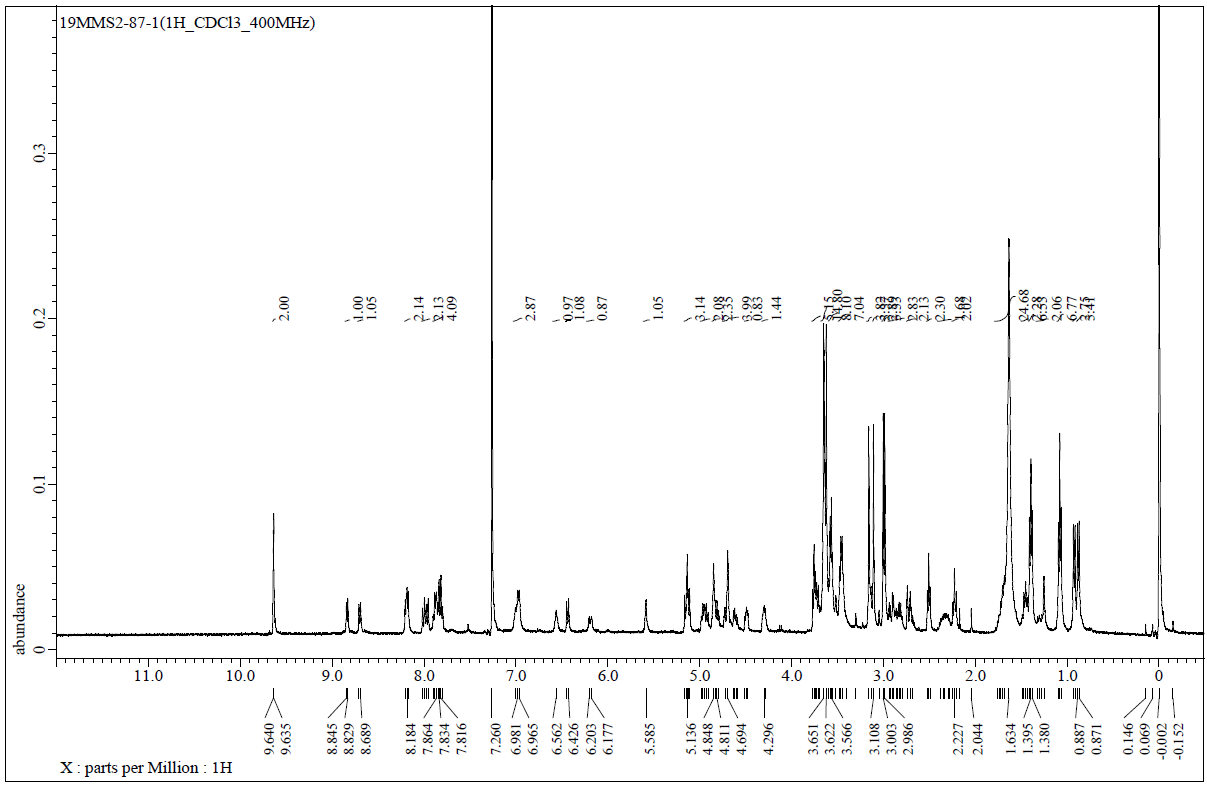
Compound **27**

**29**

(CDCl_3_, 400 MHz)


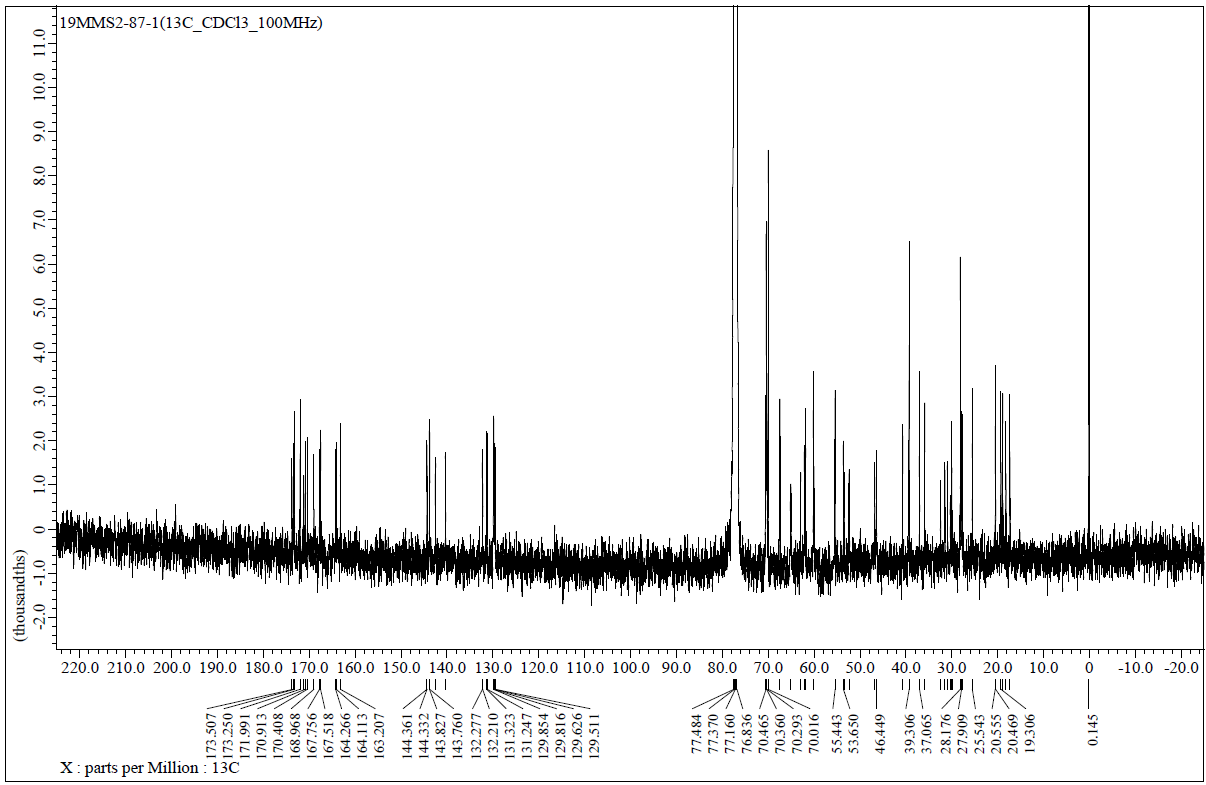


**29**

(CDCl_3_, 100 MHz)


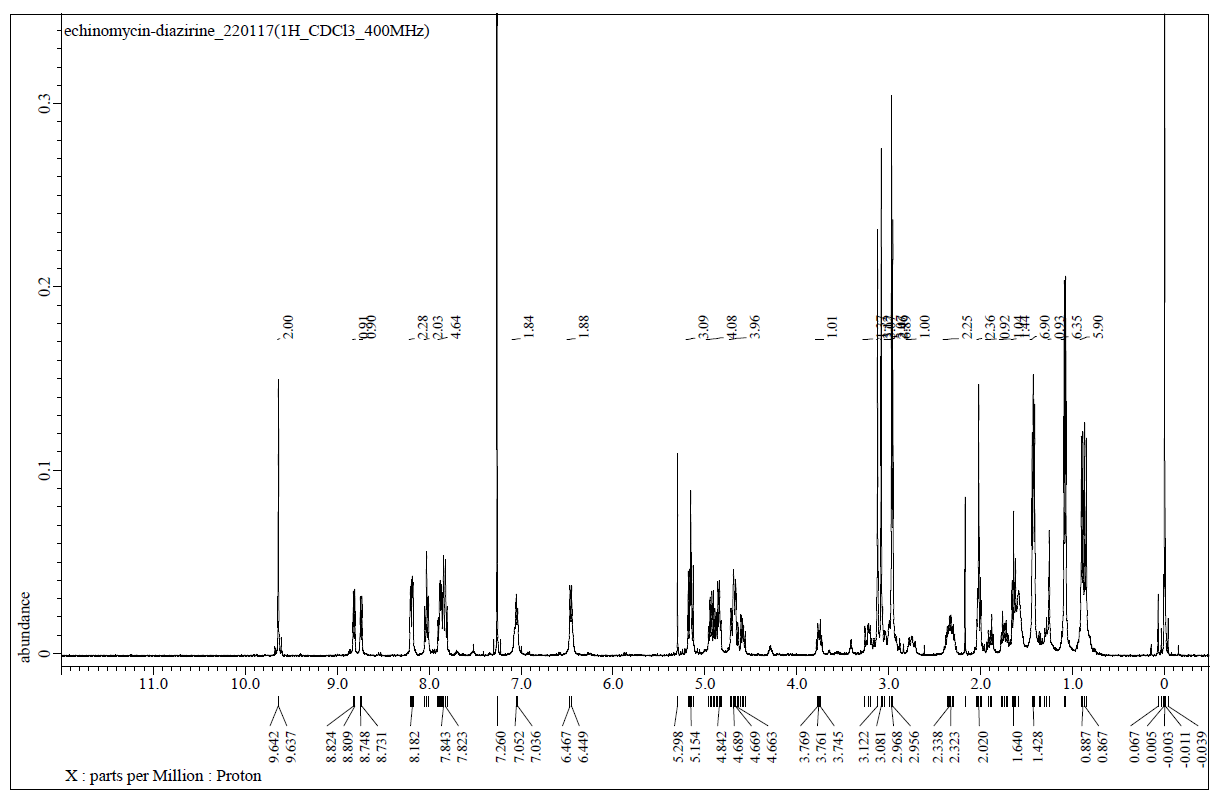
Compound **29**


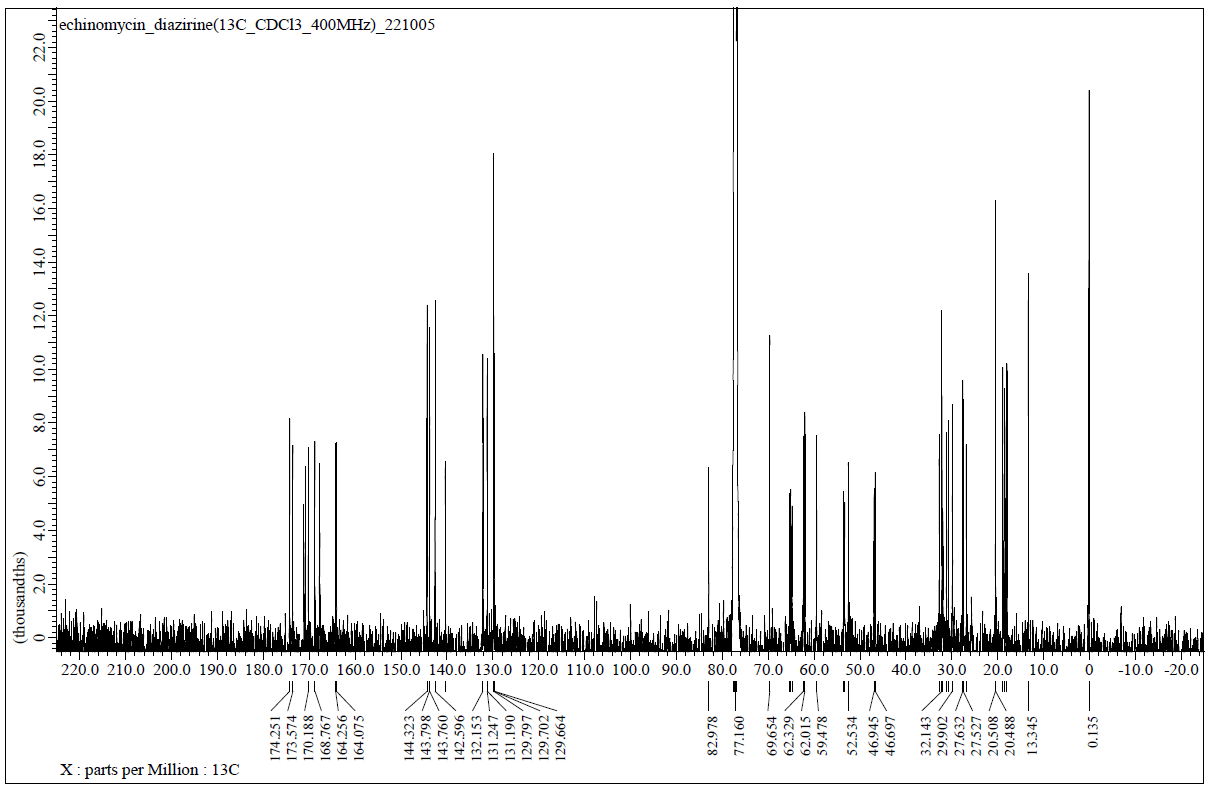


**31**

(CDCl_3_, 100 MHz)

**31**

(CDCl_3_, 400 MHz)

1. **HRMS spectrum of compounds**

Echinomycin (**1**)
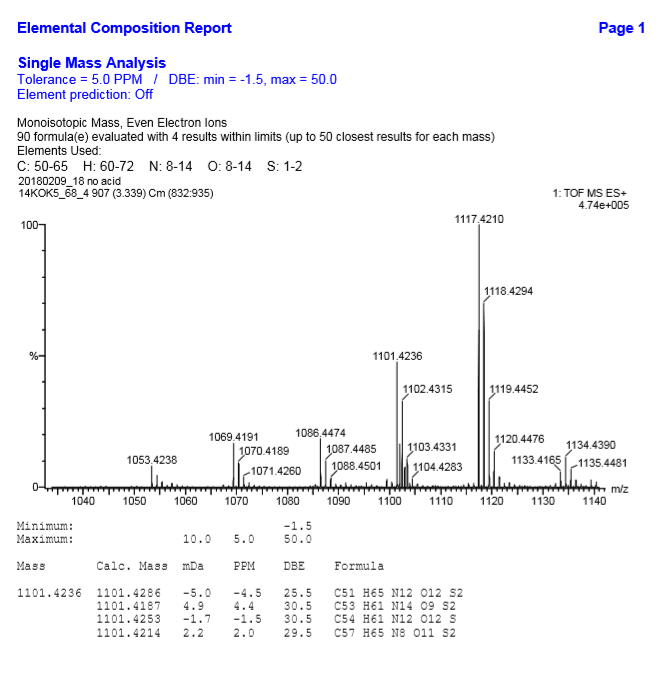


Compound **2**


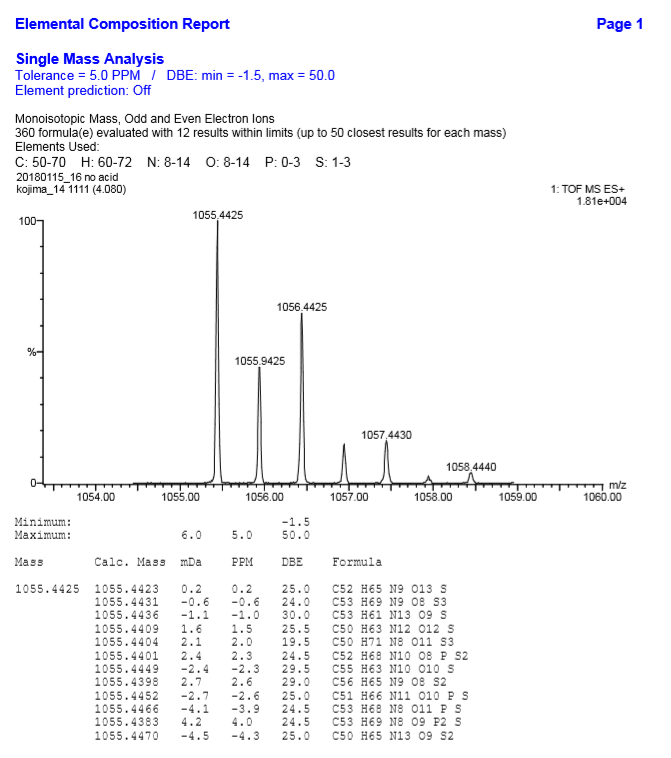


Compound **3**


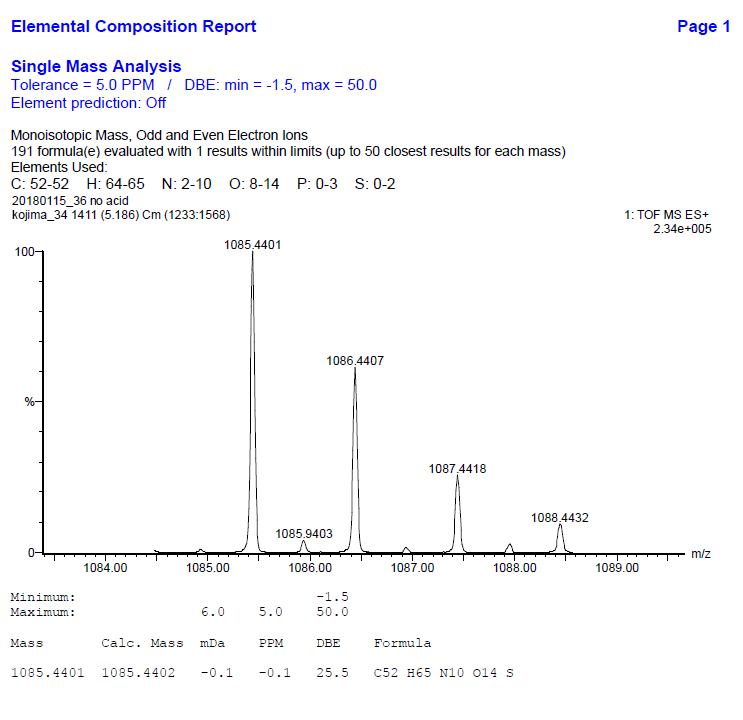


Compound **4**


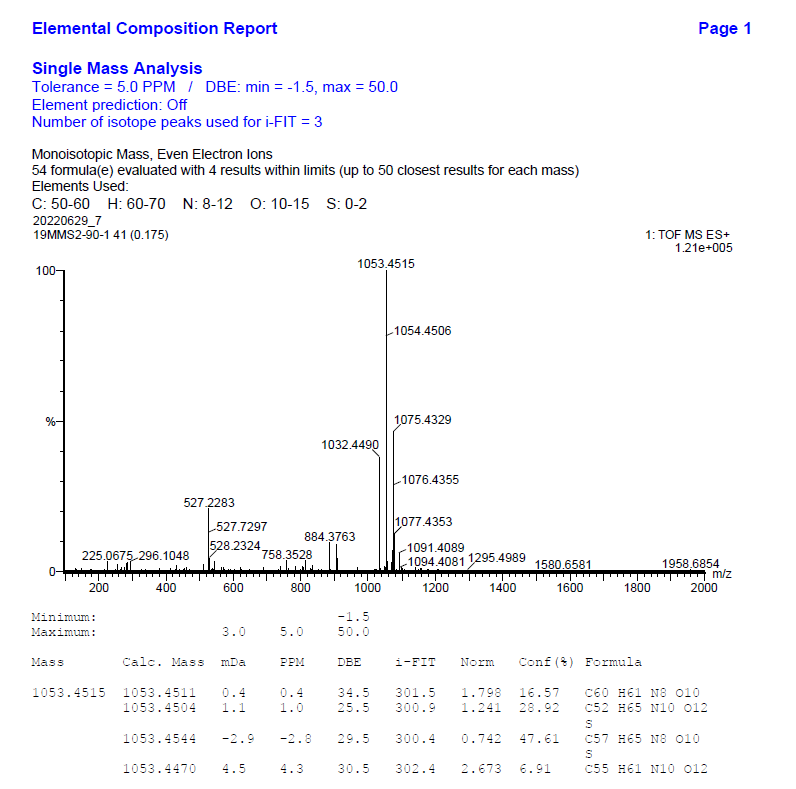


Compound **5**


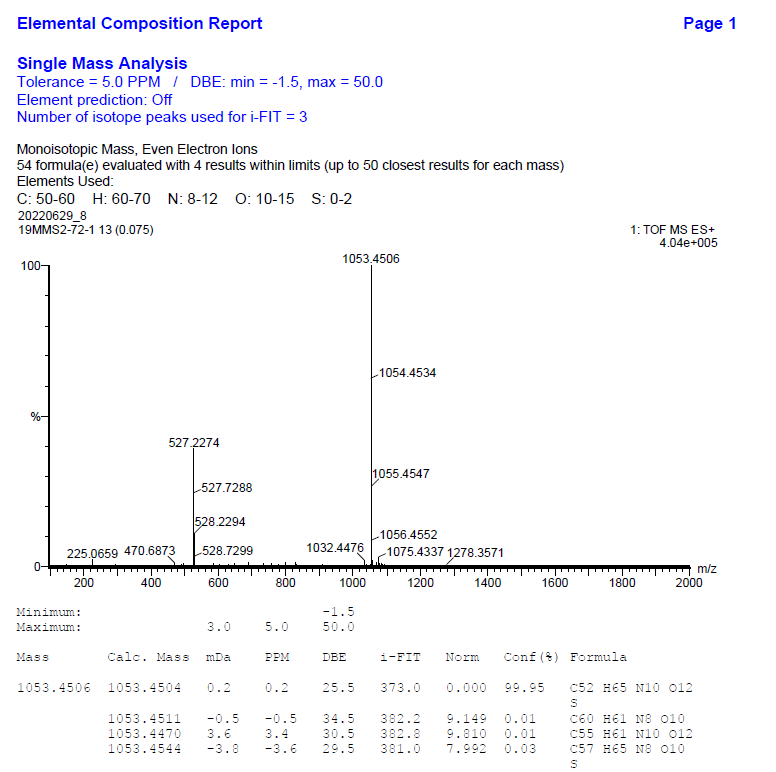


Compound **6**


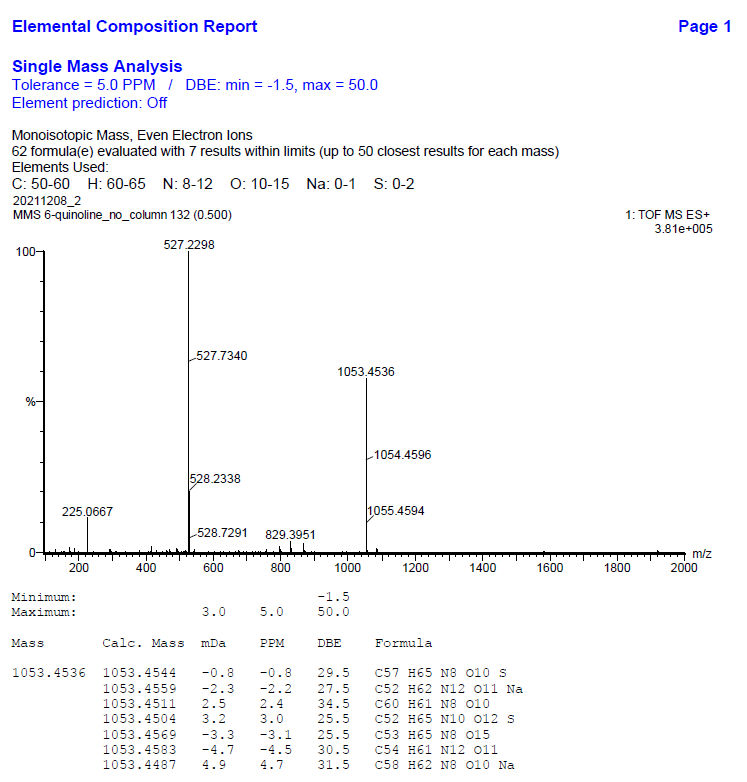


Compound **7**


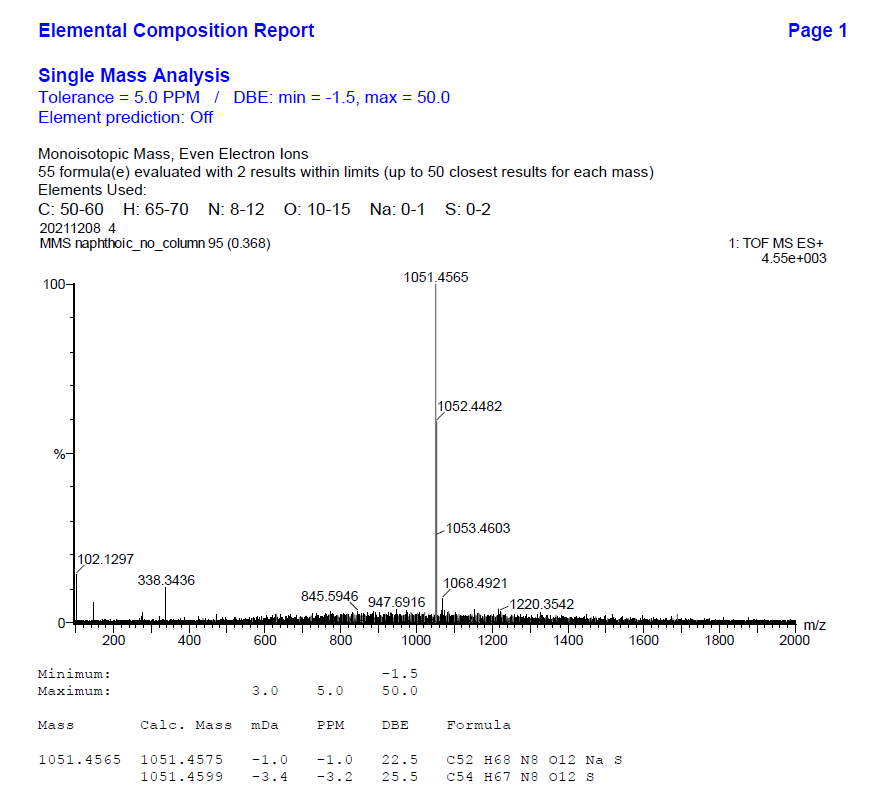


Compound **8**


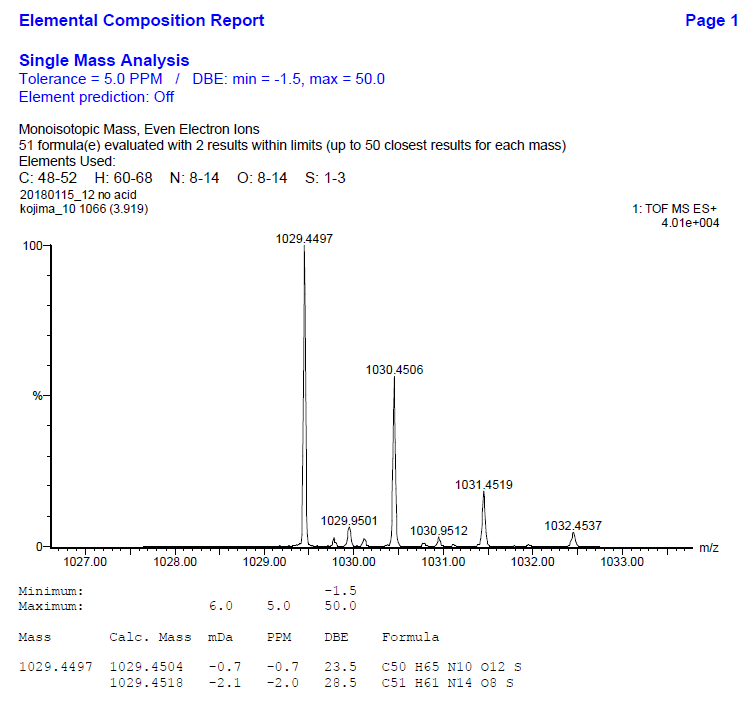


Compound **9**


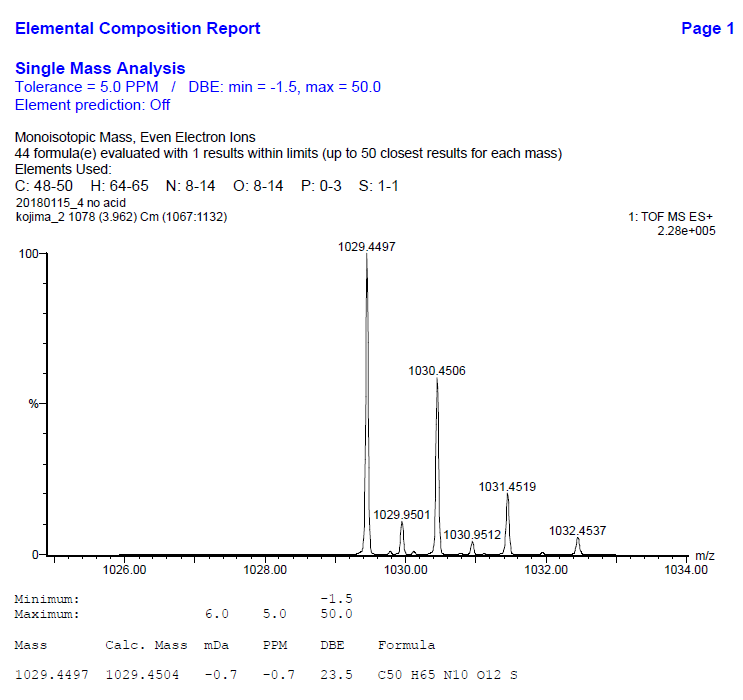


Compound **10**


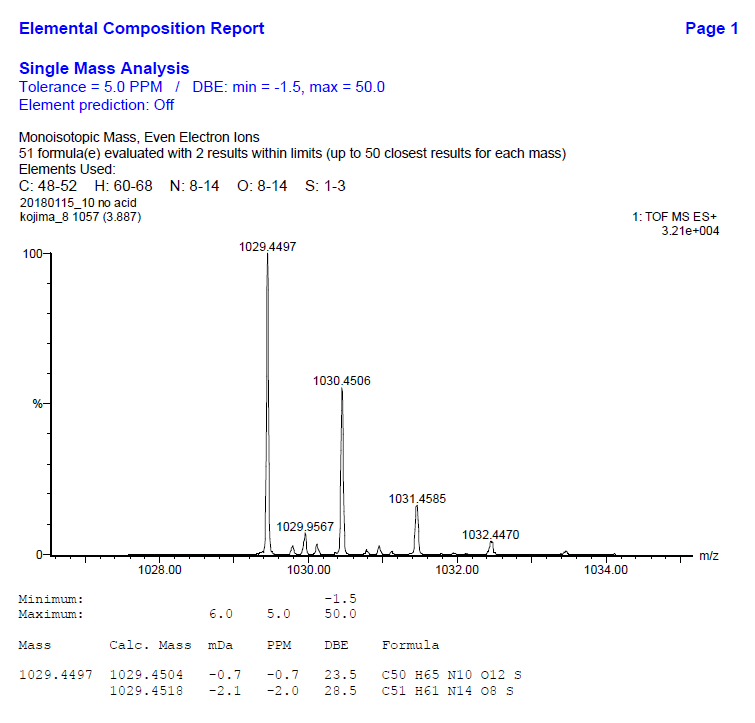


Compound **11**


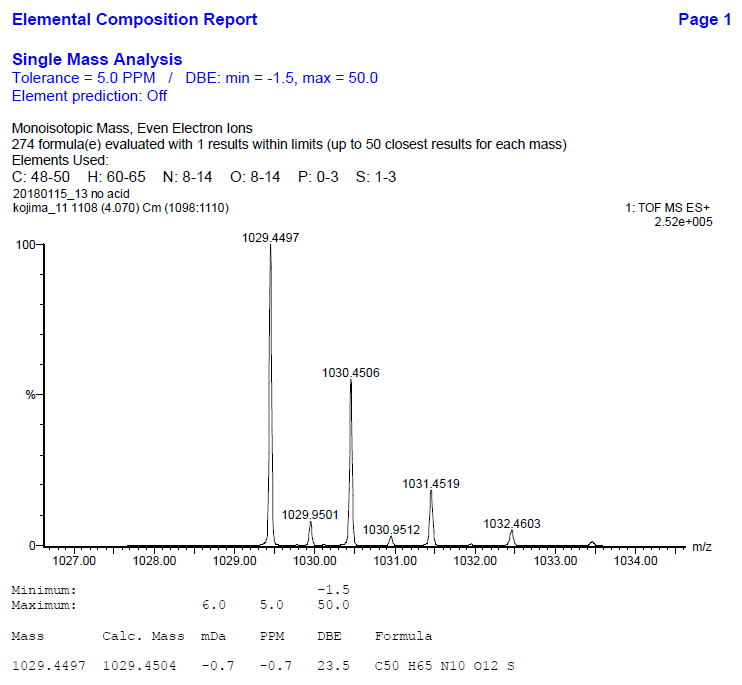


Compound **12**


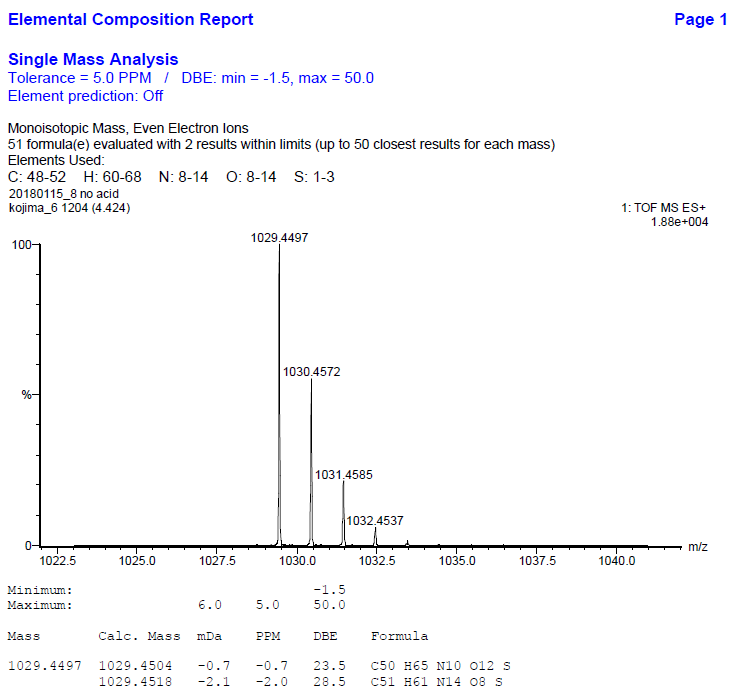


Compound **13**


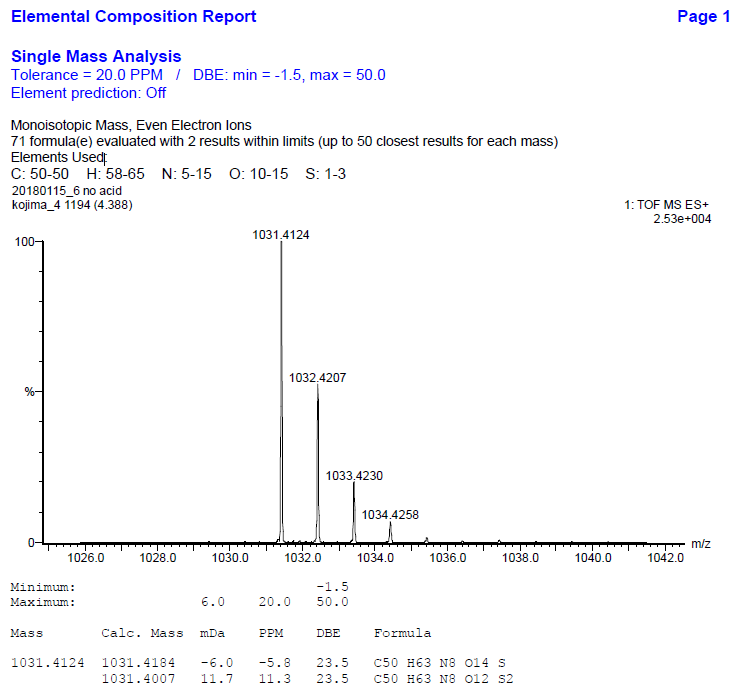


Compound **14**


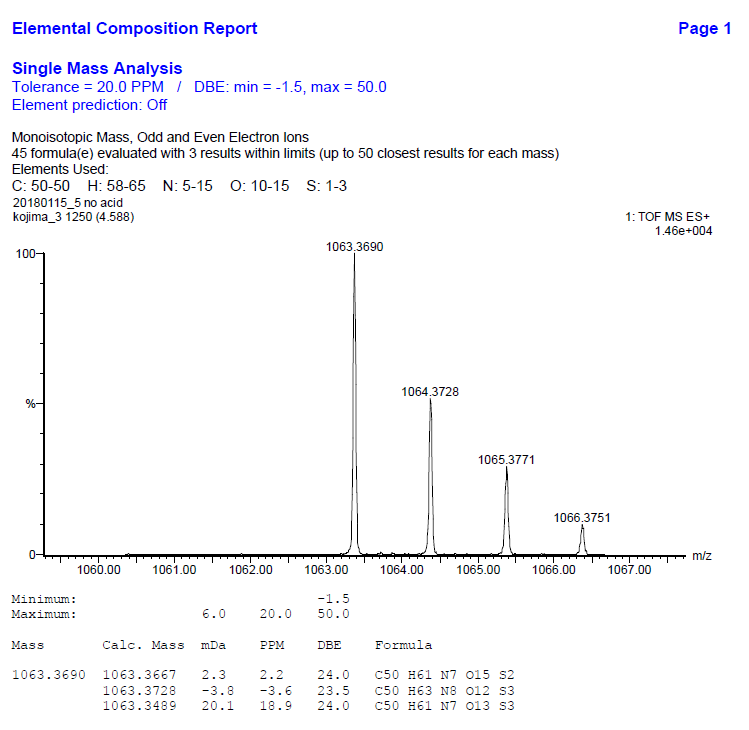


Compound **15**


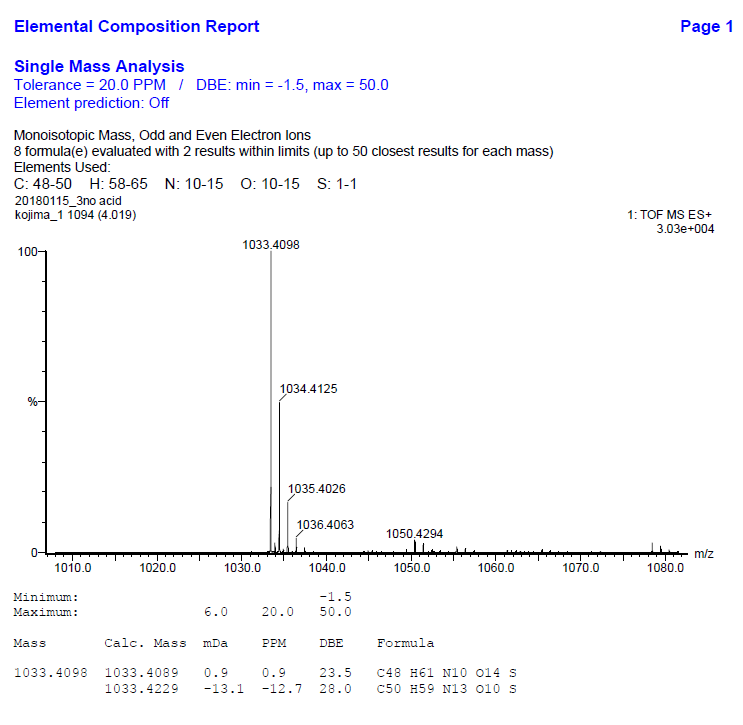


Compound **16**


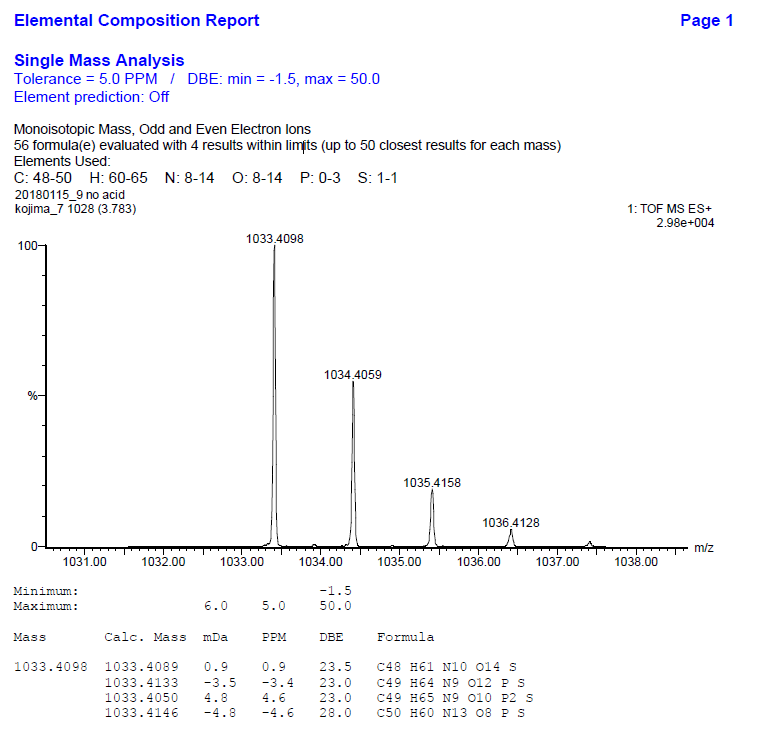


Compound **17**


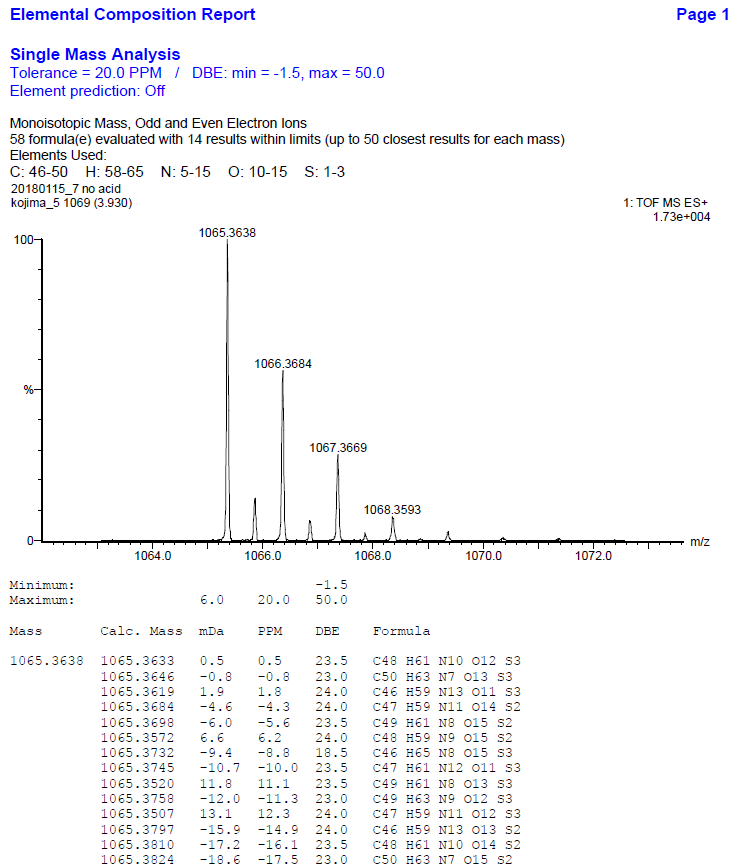


Compound **18**


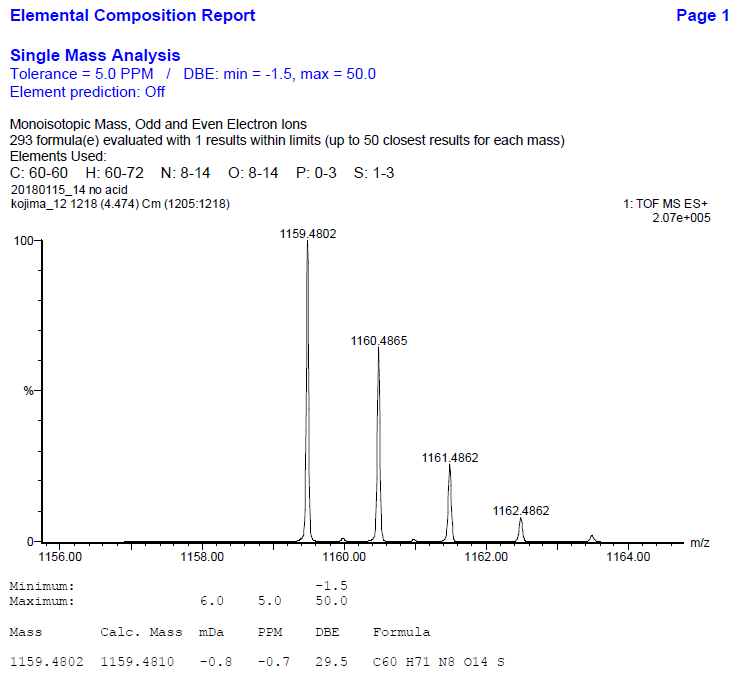


Compound **19**


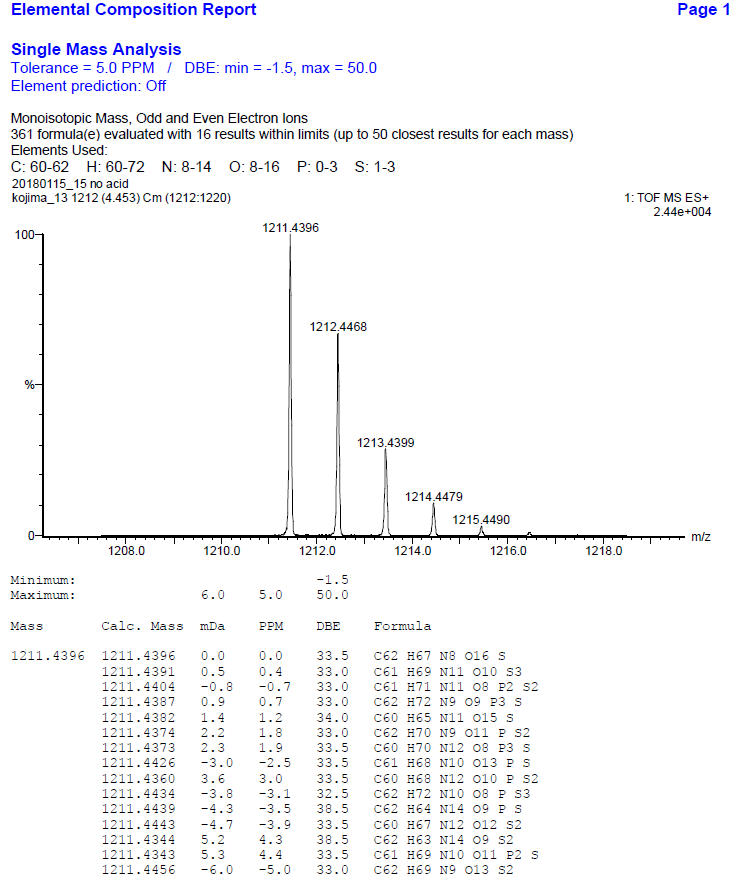


Compound **25**

**
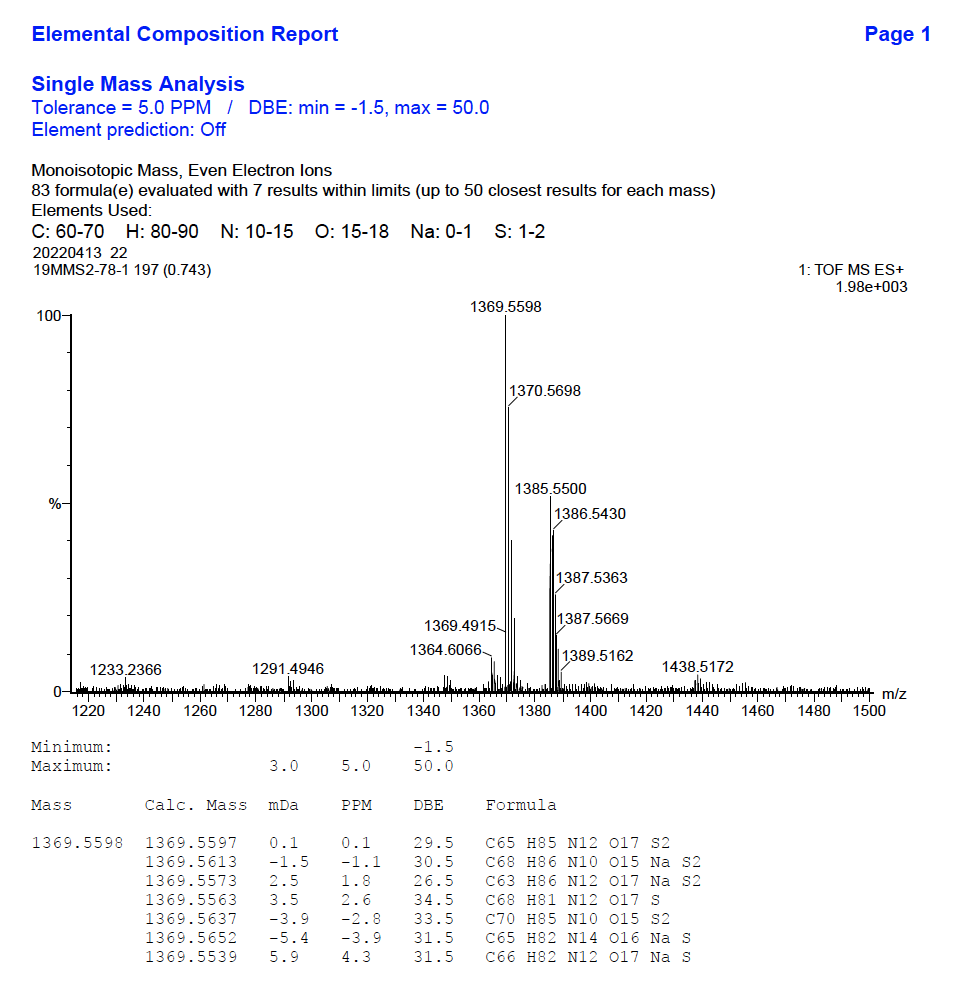
**

Compound **27**


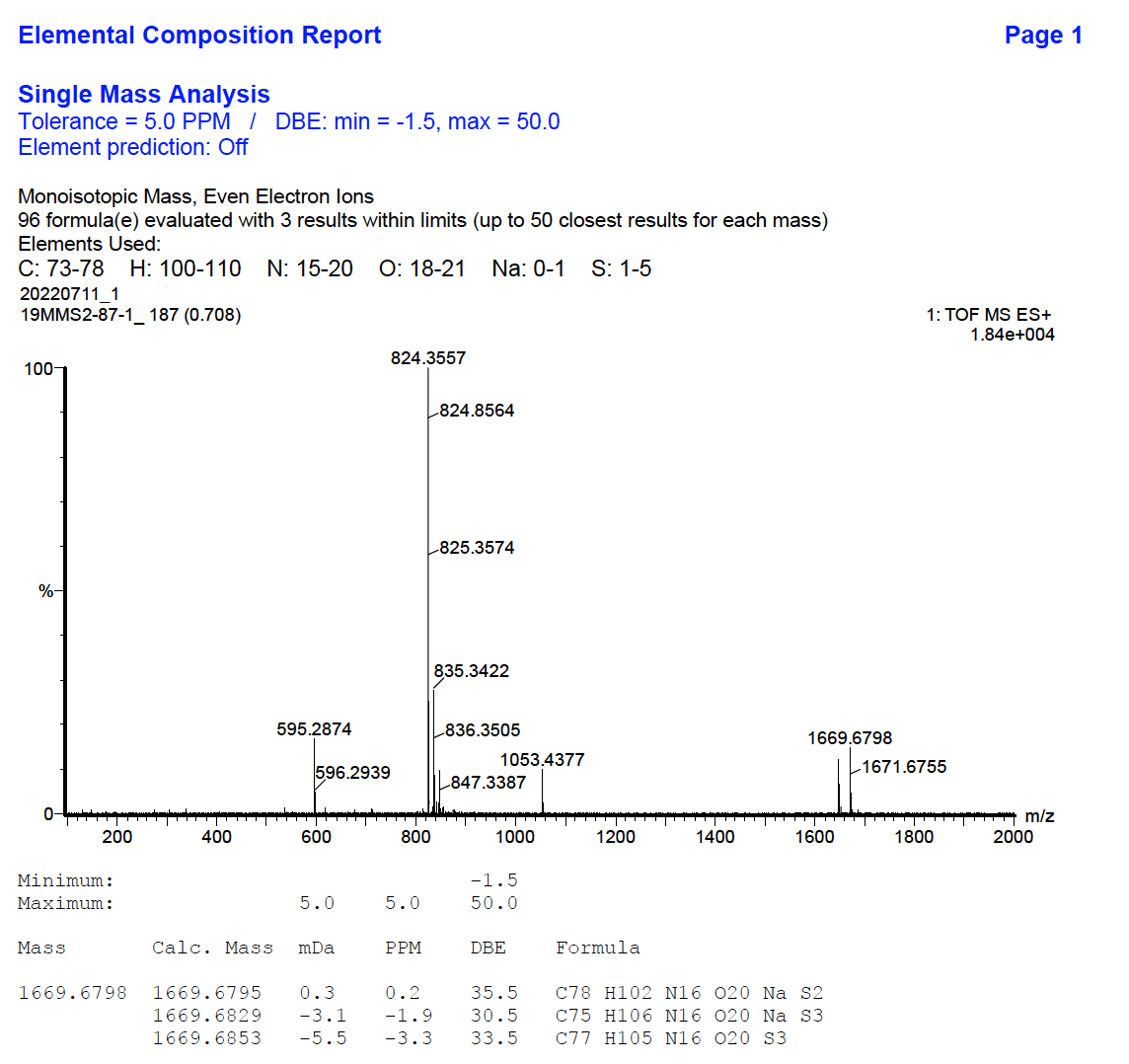


Compound **29**


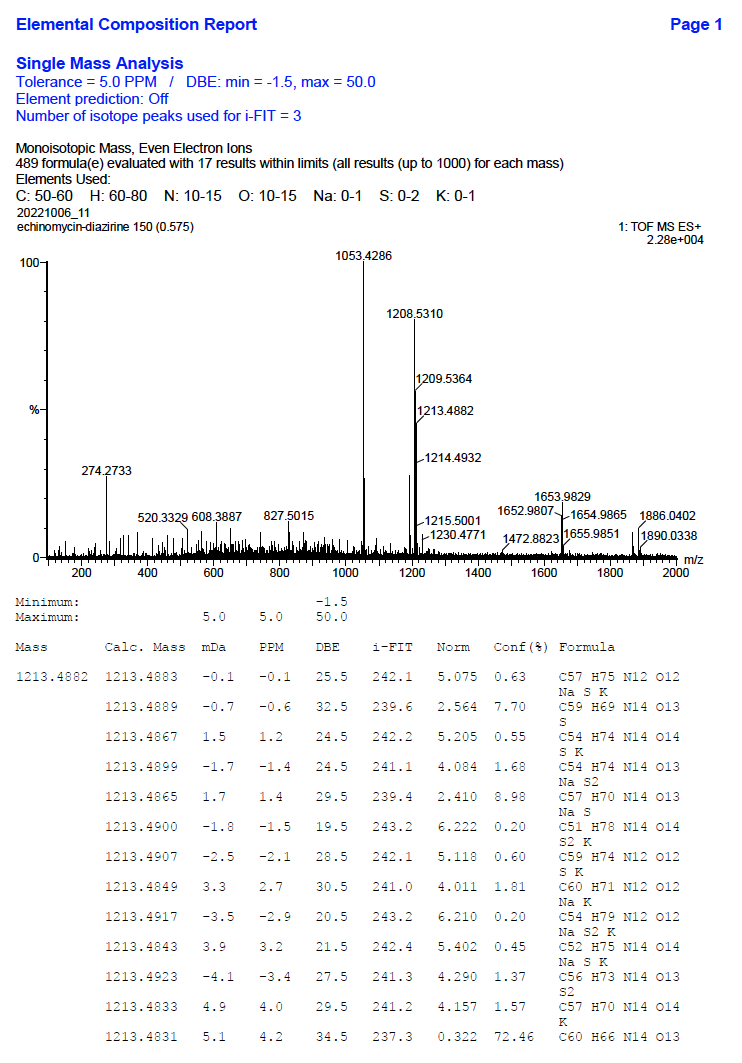


1. **Investigation of maximum tolerated doses**

| 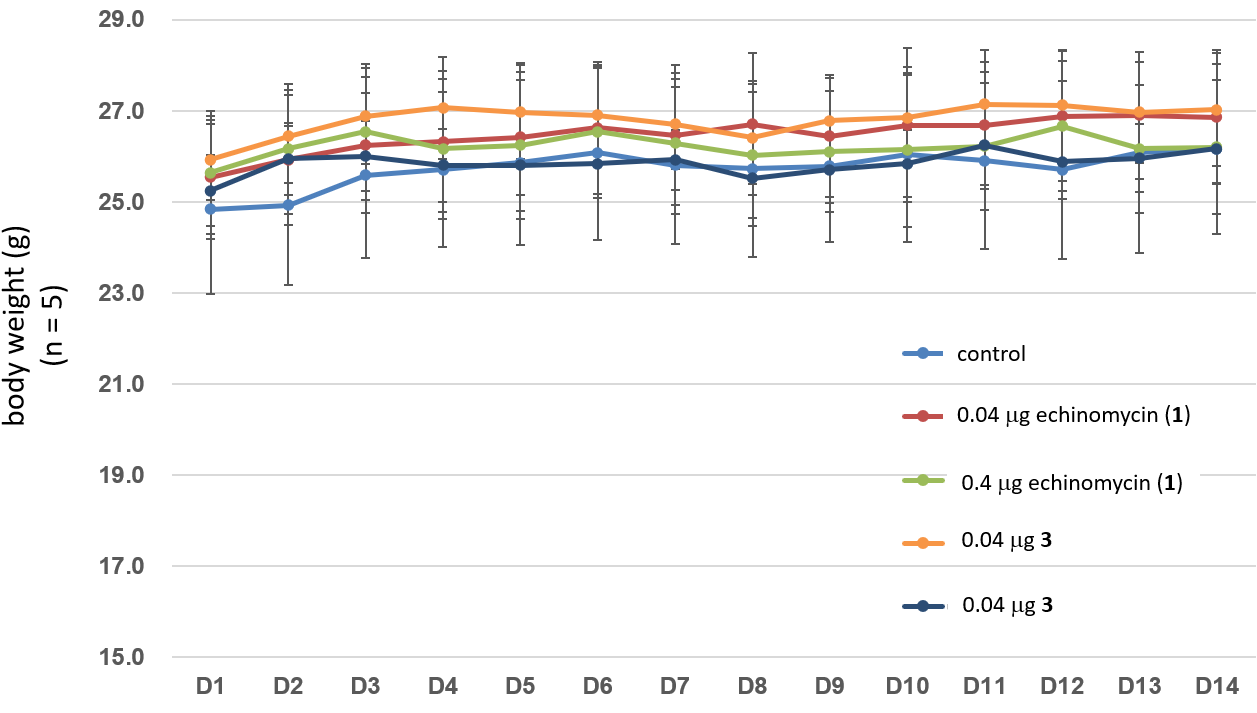 |
| --- |
| **Figure S1.** Effect of echinomycin (**1**) and **3** on the body weight of mice. Male BALB/C nude mice were administered by intraperitoneal injection with the vehicle, echinomycin and **3**, at dosages of 0.04 and 0.4 μg/mice, every day for 14 days. The error bars show the s.d. (n = 5) |

1. **Blood hematological examination of mice treated with 1 and 3**

| **** | | |
| --- | --- | --- |
| **Figure S2.Blood hematological examination of mice treated with 1 and 3** |  |  |

1. **Full-length gels and blots for Figure 4**

The membranes were cut based on molecular weight before antibody hybridization.

**cleaved caspase-3**

**
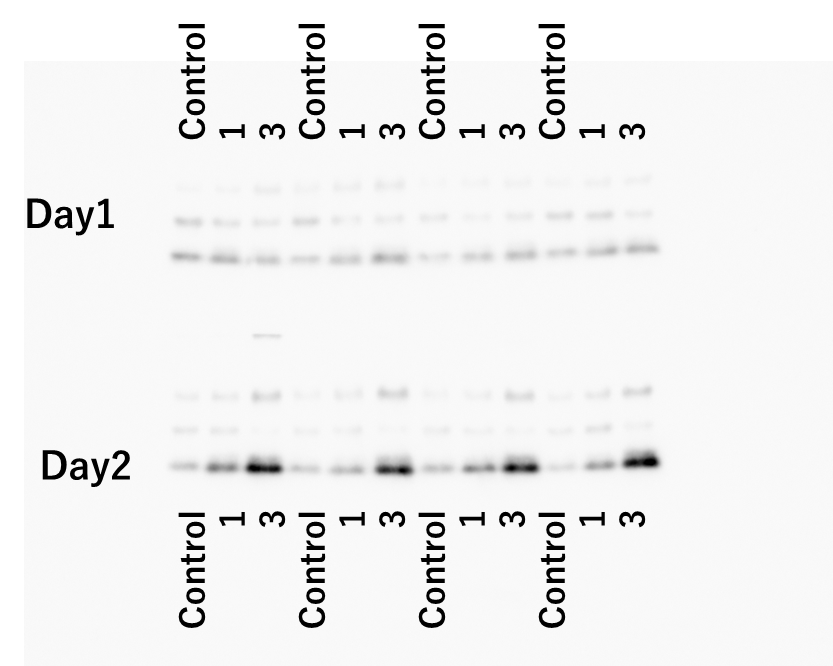
**

**
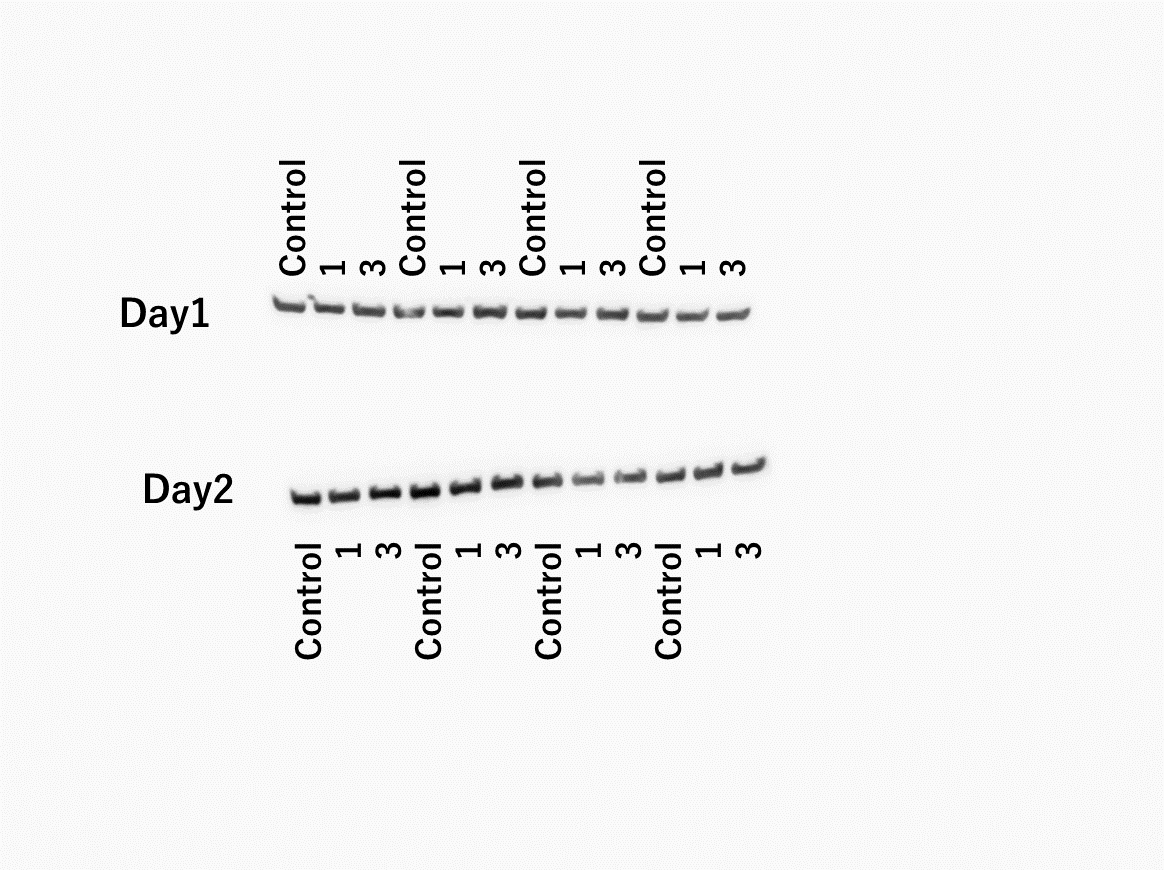
**

**actin**

1. **Concentration-response curves of *in vitro* cytotoxic activity assay**
2. **SW620**

| **** | **** |
| --- | --- |
| **** | **** |
| **** | **** |

1. **MIA-PaCa2**

| **** | **** |
| --- | --- |
|  |  |
|  |  |
