# Supplementary material for: Synthesis and biological evaluation of echinomycin analogues as potential colon cancer agent
Source: Sci Rep. 2024 Apr 1;14:7628. doi: 10.1038/s41598-024-58196-3 (PMC10985088; doi:10.1038/s41598-024-58196-3)
Supplement: Supplementary file 3 — Supplementary Information 3. [file 41598_2024_58196_MOESM3_ESM.docx]

**Supplementary Information**

The Supplementary Information, which includes preparation of compounds, ^1^H, ^13^C NMR spectrum of compounds, MS spectrum of compounds, and chromatograms of analysis, is available free of charge.
